# Multiomics of parkinsonism cynomolgus monkeys highlights significance of metabolites in interaction between host and microbiota

**DOI:** 10.1038/s41522-024-00535-3

**Published:** 2024-07-26

**Authors:** Jiang-Mei Gao, Shou-Yue Xia, Geoff Hide, Bi-Hai Li, Yi-Yan Liu, Zhi-Yuan Wei, Xiao-Ji Zhuang, Qing Yan, Yun Wang, Wei Yang, Jian-Huan Chen, Jun-Hua Rao

**Affiliations:** 1 Guangdong Key Laboratory of Animal Conservation and Resource Utilization, Guangdong Public Laboratory of Wild Animal Conservation and Utilization, Institute of Zoology, Guangdong Academy of Sciences, Guangzhou, Guangdong, China; 2 Laboratory of Genomic and Precision Medicine, Wuxi School of Medicine, Jiangnan University, Wuxi, Jiangsu China; 3 Joint Primate Research Center for Chronic Diseases, Jiangnan University and Institute of Zoology, Guangdong Academy of Sciences, Guangzhou, Guangdong China; 4 Biomedical Research Centre and Ecosystems and Environment Research Centre, School of Science, Engineering and Environment, University of Salford, Salford, M5 4WT UK; 5 Zhuzhou Central Hospital, Zhuzhou, Hunan China; 6 Longhua Innovation Institute for Biotechnology, Shenzhen University, Shenzhen, Guangdong China; 7 Guangzhou Bay Area Institute of Biomedicine, Guangdong Lewwin Pharmaceutical Research Institute Co., Ltd., Guangdong Provincial Key Laboratory of Drug Non-Clinical Evaluation and Research, Guangzhou, Guangdong China

**Keywords:** Clinical microbiology, Sequencing

## Abstract

The gut microbiota has been demonstrated to play a significant role in the pathogenesis of Parkinson’s disease (PD). However, conflicting findings regarding specific microbial species have been reported, possibly due to confounding factors within human populations. Herein, our current study investigated the interaction between the gut microbiota and host in a non-human primate (NHP) PD model induced by 1-methyl-4-phenyl-1,2,3,6-tetrahydropyridine (MPTP) using a multi-omic approach and a self-controlled design. Our transcriptomic sequencing of peripheral blood leukocytes (PBL) identified key genes involved in pro-inflammatory cytokine dysregulation, mitochondrial function regulation, neuroprotection activation, and neurogenesis associated with PD, such as *IL1B*, *ATP1A3*, and *SLC5A3*. The metabolomic profiles in serum and feces consistently exhibited significant alterations, particularly those closely associated with inflammation, mitochondrial dysfunctions and neurodegeneration in PD, such as TUDCA, ethylmalonic acid, and L-homophenylalanine. Furthermore, fecal metagenome analysis revealed gut dysbiosis associated with PD, characterized by a significant decrease in alpha diversity and altered commensals, particularly species such as *Streptococcus*, *Butyrivibrio*, and *Clostridium*. Additionally, significant correlations were observed between PD-associated microbes and metabolites, such as sphingomyelin and phospholipids. Importantly, PDPC significantly reduced in both PD monkey feces and serum, exhibiting strong correlation with PD-associated genes and microbes, such as *SLC5A3* and *Butyrivibrio* species. Moreover, such multi-omic differential biomarkers were linked to the clinical rating scales of PD monkeys. Our findings provided novel insights into understanding the potential role of key metabolites in the host-microbiota interaction involved in PD pathogenesis.

## Introduction

Parkinson’s disease (PD) is a chronic, progressive, and irreversible neurodegenerative disorder. PD affects approximately 1% of individuals aged over 60 years and around 3% of those older than 80 years^1,2^. In China, 4.94 million of the population are predicted to be affected by PD by 2030, accounting for half of the global PD patients^3^. Notably, clinical data indicate that men have twice the risk of developing PD compared to women^4^.

PD involves various pathological processes, such as neuroinflammation, mitochondrial dysfunction, oxidative stress, and impaired protein degradation^5–8^. Recent research has demonstrated that alterations related to PD can also be identified in peripheral blood, such as metabolic dysregulation. Furthermore, some of these changes may even manifest prior to the onset of PD’s motor dysfunction symptoms^9^. Therefore, comprehensive multi-omic investigations of PD are needed to identify novel biomarkers for diagnosis and effective intervention and provide new insight into the underlying disease mechanisms.

Furthermore, motor symptoms in PD patients are usually accompanied by gastrointestinal dysfunction^10,11^. Additionally, gut microbiota dysbiosis has been reported in PD, emphasizing the crucial role of gut microbiota in chronic inflammation of intestinal epithelia and even neuroinflammation through the gut microbiota-brain axis^12,13^. Moreover, disrupted metabolites associated with altered gut microbiota, such as neuroprotective kynurenine and neurotoxic polyamines, may contribute to PD-related pathway dysregulation^14,15^. However, how metabolites participate in host-microbiota interaction in PD remains to be elucidated.

The MPTP-induced animal models, particularly the non-human primate (NHP) models of PD, are essential tools for investigating the interaction between the gut-microbiota and host in both basic and translational research. Compared to MPTP-induced rodent models, PD NHP models offer a higher similarity to human patients in terms of their brain structure and gut microbiota composition^16^. However, there are few PD NHP model studies on biomarkers and their roles in the gut-brain axis and the disease.

**Fig. 1:**
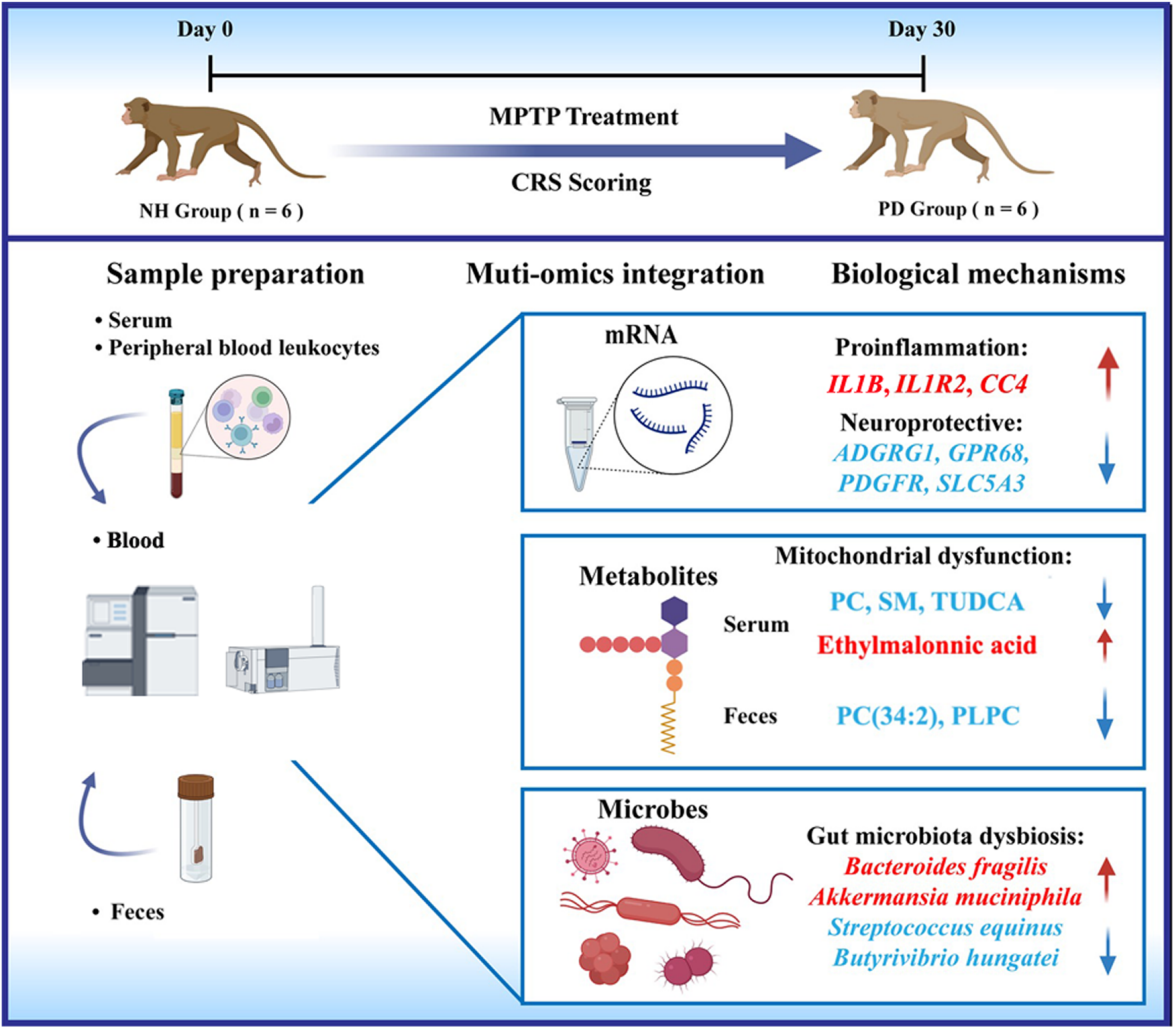
Study schematic design. A non-human primate (NHP) PD model induced by MPTP is analyzed for alterations in gut microbiota, peripheral blood leukocytes (PBL) transcriptome, and serum and fecal metabolome to identify potential biomarkers in the gut-microbiota-brain axis using a multi-omic approach and a self-controlled design. The scheme is visualized by Figdraw (https://www.figdraw.com/).

Recent studies reported contradictory results regarding either microbial diversity or relative abundance associated with PD, which could be attributed to confounding factors in human populations, such as diet and medications, and sequencing methodological inconsistencies^17,18^. Therefore, our current study employed a multi-omic approach along with a self-controlled design (Fig. 1) to establish an MPTP-induced PD model in cynomolgus monkeys (*Macaca fascicularis*) to explore the metabolite-meditated microbe-host interaction in PD pathogenesis and minimize the effects of possible confounding factors.

## Results

### Manifestation of motor dysfunction in PD monkeys

The CRS scores for the six MPTP-treated monkeys (PD-1 to PD-6) were determined through double-blind evaluations. One month after MPTP treatment, all six monkeys exhibited motor dysfunction, including persistent bradykinesia, rigidity, posture abnormalities, gait imbalances, moderate to severe tremors, and significant impairments in gross motor skills (Table 1). Such findings confirmed the successful establishment of the PD monkey model as these symptoms closely resemble those observed in PD patients.

**Table 1:** Clinical rating scale scores of PD monkeys treated with MPTP

Parkinsonian motor signs	PD-1	PD-2	PD-3	PD-4	PD-5	PD-6
*Before MPTP treatment*						
*Total CRS scales*	0	0	0	0	0	0
*After MPTP treatment*						
Tremor (0–3)	1.5	2	3	3	2	2.5
Bradykinesia (0–3)	2	2.5	2	2	2	2.5
Rigidity (0–3)	1.5	3	2	2	1.5	2
Posture (0–3)	1.5	2	2	2	1.5	2
Balance (0–3)	1.5	2	2	2	1.5	2
Gross motor skills (0–3)	1.5	1.5	1.5	1.5	1.5	1.5
*Total CRS scores*	9.5	13	12.5	12.5	10	12.5

### PBL transcriptomics unveiled alterations in PD-related pathways

**Fig. 2:**
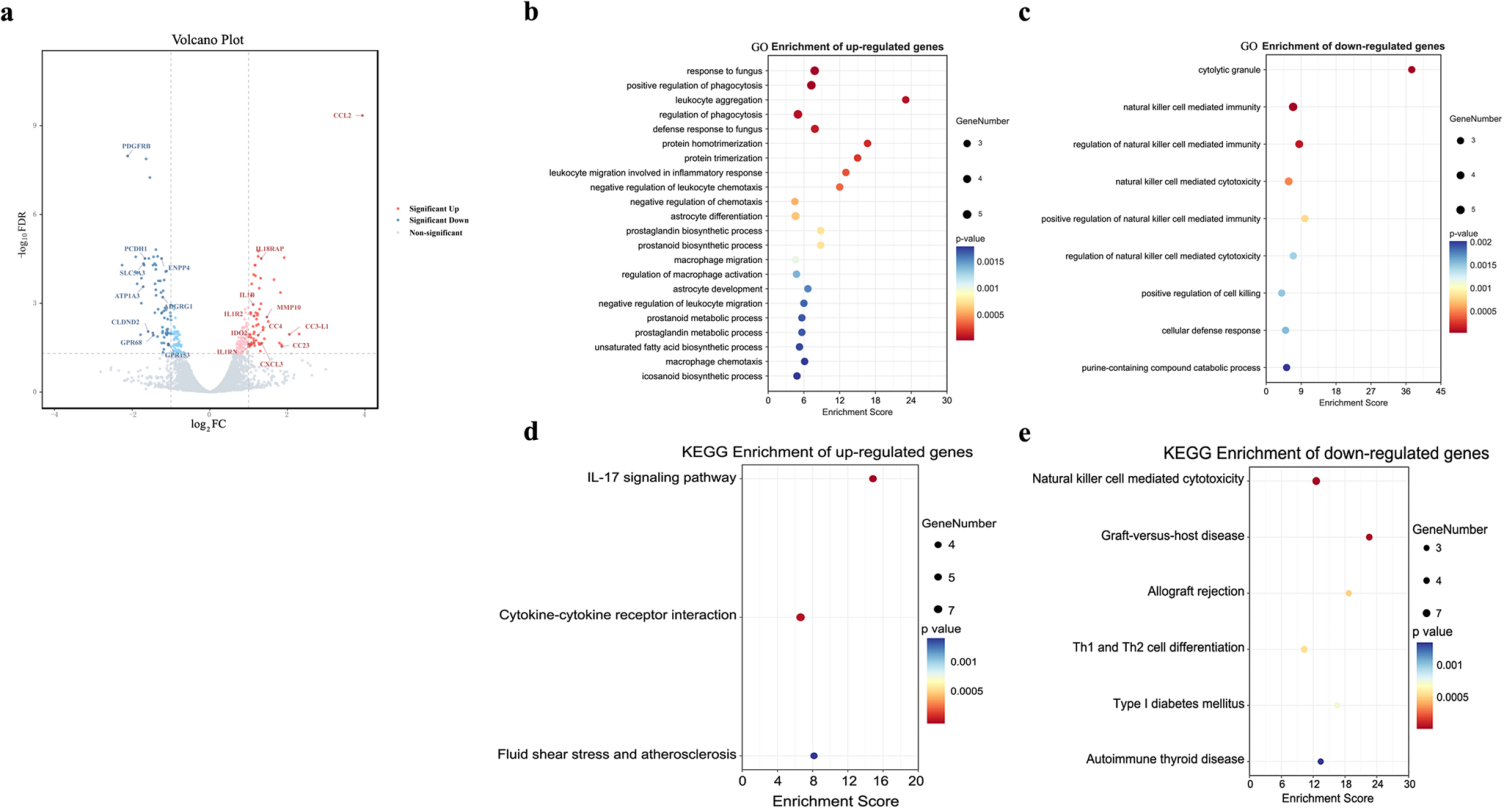
DEGs and GO analysis in PBL from NH and PD monkeys (*n* = 6). **a** Volcano plot of mRNA expression in PBL from PD monkeys before and after MPTP treatment. Genes with edgeR FDR < 0.05 and |log_2_FC| > 1 are in blue if down-regulated and red if up-regulated. The Y axis shows empirical edgeR *P*-values. **b**, **c** GO biological processes enriched by up-regulated and down-regulated DEGs in PD monkeys. The top 20 terms with FDR < 0.05 ranked by *p*-values are shown. **d**, **e** KEGG pathways enriched by up-regulated and down-regulated DEGs in PD monkeys (FDR < 0.05). NH neurologically healthy, PD Parkinson’s disease, GO gene ontology, KEGG Kyoto Encyclopedia of Genes and Genomes, PBL peripheral blood leukocytes, DEGs differentially expressed genes, defined as those with FDR < 0.05 and |log_2_FC| > 1.

Previous studies have revealed that mitochondrial dysfunction, oxidative stress, neuroinflammatory processes, and loss of midbrain dopaminergic neurons play vital roles in the pathogenesis of PD^5,6^. Therefore, RNA-Seq analysis (Supplementary Table 1a) of PBL from the PD monkeys was performed before and after MPTP treatment to compare the expression profiles. 154 DEGs were identified as having significant changes with FDR < 0.05 and |log_2_FC| > 1, including 78 down-regulated and 76 up-regulated (Fig. 2a, Supplementary Table 1b).

To better understand the biological functions of these DEGs, GO analysis was performed. GO analysis showed that up-regulated DEGs in PD monkeys were mainly enriched in the biological processes (BPs) related to immunity and inflammatory response such as “leukocyte aggregation and migration involved in the inflammatory response,” “defense and response to fungus,” and “astrocyte differentiation” (Fig. 2b). In contrast, down-regulated genes were the most enriched in BPs related to natural killer cell-mediated immunity (Fig. 2c). KEGG analysis showed that pathways enriched in up-regulated genes included “IL-17 signaling pathway”, “Cytokine-cytokine receptor interaction”, and “Fluid shear stress and atherosclerosis” (Fig. 2d). Pathways enriched in down-regulated genes included “Natural killer cell mediated cytotoxicity”, “Th1 and Th2 cell differentiation”, “Type I diabetes mellitus”, “Allograft rejection”, “Autoimmune thyroid disease”, and “Graft-versus-host disease” (Fig. 2e).

**Fig. 3:**
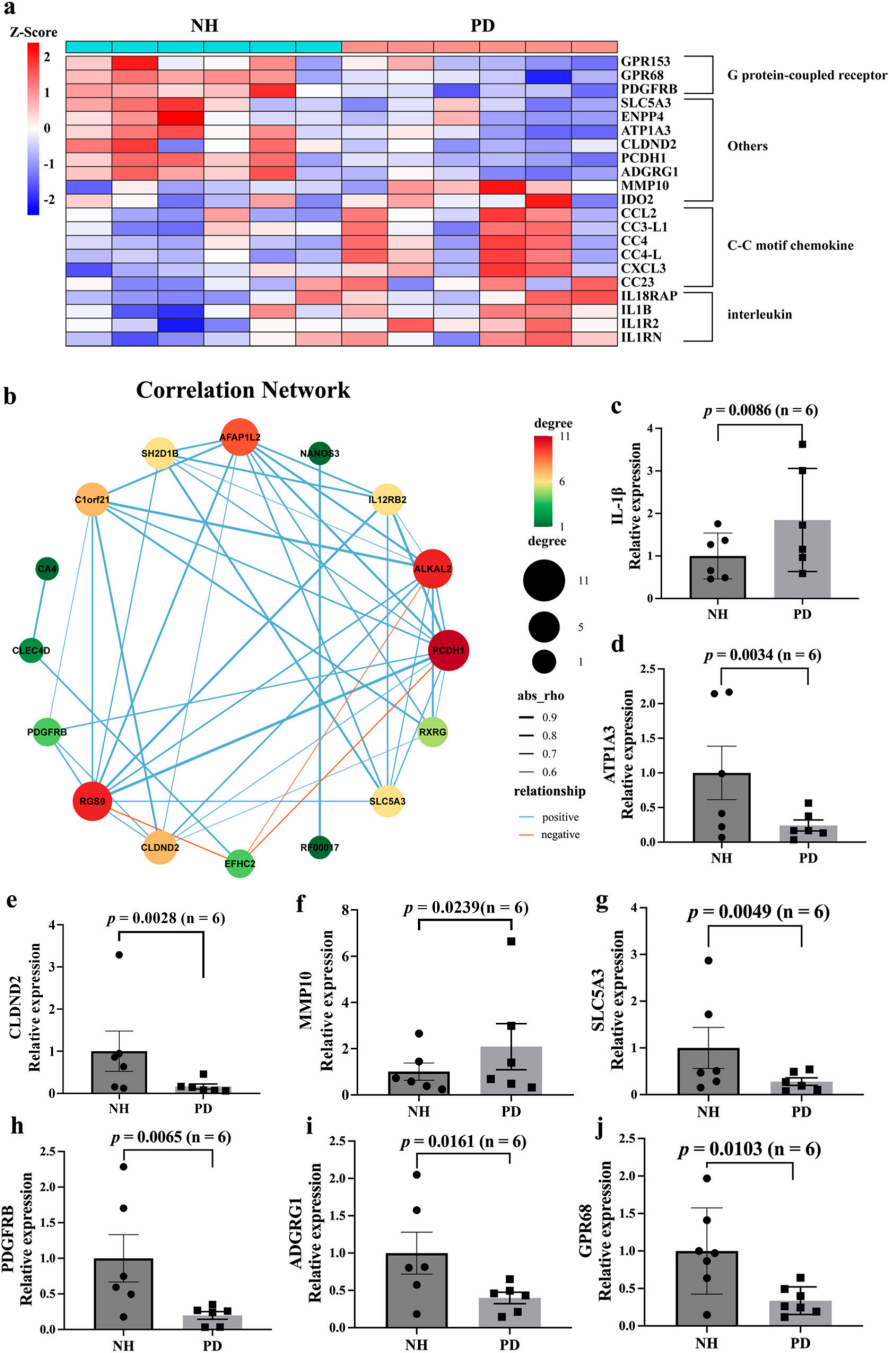
Representative DEGs and interactive gene network associated with PD. **a** Heatmap showing selected DEGs of pro-inflammatory cytokines and related genes comparing NH and PD monkeys. The color indicates the Z-score of the normalized expression level (TPM). **b** An interactive gene network constructed using DEGs with edgeR FDR < 0.05 and |log_2_FC| > 1.5. The node color and size indicate the number of connected edges. Edge thickness represents the correlation coefficient (*r*) value. Red and blue lines represent negative and positive correlations, respectively. **c**–**j** qPCR validation of selected candidate DEGs. Relative expression levels are presented as mean ± standard error of the mean (SEM). Wilcoxon signed-rank tests are used to analyze the qPCR validation data. *p < 0.05; **p < 0.01. NH neurologically healthy, PD Parkinson’s disease, TPM transcripts per million reads, DEGs differentially expressed genes.

It was noted that these DEGs contained several pro-inflammatory cytokines, such as interleukin 18 receptor accessory protein (IL18RAP), interleukin 1 beta (*IL1B*), interleukin 1 receptor type 2 (*IL1R2*), and C-C motif chemokine 4 (*CC4*), that were significantly up-regulated in PD, accompanied by a significantly elevated matrix metallopeptidase 10 (*MMP10*) (Fig. 3a).

Significantly down-regulated DEGs in PD monkeys mainly encoded neuroprotective or neurogenesis proteins and could be involved in inflammation, neuronal damage, mitochondrial function impairment, and blood-brain/gut barriers, such as claudin domain containing 2 (*CLDND2*) encoding a tight junction protein component of blood-brain/gut barriers, adhesion G protein-coupled receptor G1 (*ADGRG1*), G protein-coupled receptor 68 (*GPR68*), protocadherin-1 (*PCDH1*), platelet-derived growth factor receptor beta (*PDGFRB*) and solute carrier family 5 member 3 (*SLC5A3*) (Fig. 3a). Furthermore, the gene encoding α subunit of the Na^+^/K^+^ ATPase (*ATP1A3*), a known disease-causing gene for PD that expresses almost exclusively in neurons, was also significantly downregulated in PD monkeys (Fig. 3a).

Furthermore, as shown in Fig. 3b, gene-gene interactive networks identified DEGs as hub genes in the network, such as *PCDH1*, *CLDND2*, and *SLC5A3*, which were selected and validated using real-time qPCR (Fig. 3c–j). Thus, these DEGs were identified as candidate PD biomarkers and included in the subsequent analysis.

### Gastrointestinal (GI) dysfunction and gut microbiota dysbiosis in PD monkeys

**Fig. 4:**
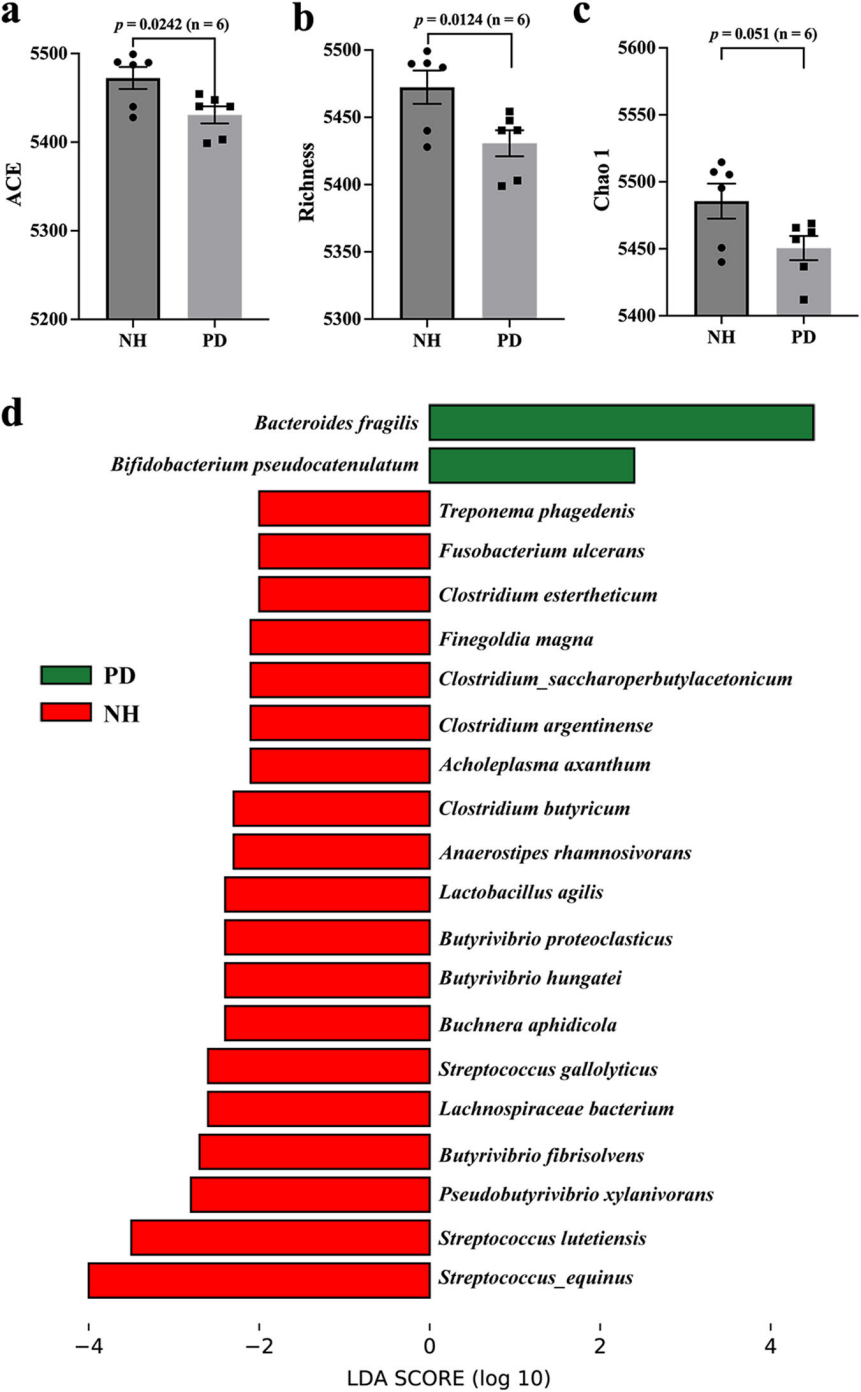
Alteration in the gut microbiota after MPTP treatment. **a**–**c** Changes in ACE, richness, and Chao1 indices after MPTP treatment. Alpha-diversity indices are shown as mean ± SEM and compared between groups using the Wilcoxon signed-rank test. **d** Substantial changes in the relative abundance of the gut microbiota species after MPTP treatment. Results are based on LEfSe analysis (*p* < 0.05 and LDA > 2.0). Representative species are shown, and the full list of differential gut microbes is included in Supplementary Fig. 2. ACE, Abundance-Based Coverage Estimates. LDA log-linear discriminant analysis. *p < 0.05; **p < 0.01.

Most of the PD monkeys developed constipation before manifestation of motor impairment. We performed metagenomic sequencing to analyze the gut microbiota composition and function (Supplementary Table 2). Both NH and PD monkeys shared the most abundant phyla in Firmicutes, Bacteroidetes, Spirochaetes, Proteobacteria, and Actinobacteria, accounting for more than 96% of all reads (Supplementary Fig. 1). Importantly, PD monkeys showed reduced richness and diversities of the gut microbiota than NH (Fig. 4a–c, ACE, *p* = 0.0242; Richness, *p* = 0.0124; Chao1, *p* = 0.051, Wilcoxon signed-rank test). In addition, no significant difference in beta diversity was observed between NH and PD monkeys (*p* = 0.7896, PERMANOVA).

LEfSe analysis showed significant differences at the phylum, genus, and species level (Supplementary Fig. 2a, b). LEfSe analysis identified four phyla, including two bacterial phyla (Tenericutes and Fusobacteria) and 51 genera, including two archaeal genera (*Methanobacterium* and *Methanosarcina*), and 49 bacterial genera (such as *Streptococcus, Clostridium, Mycoplasma*, and *Butyrivibrio*), all of which were enriched in NH (Supplementary Fig. 2a). LEfSe also identified 73 species associated with PD (Supplementary Fig. 2b), with the representative species shown in Fig. 4d. Among these PD-associated species, only two species, including *Bacteroides fragilis* and *Bifdobacterium pseudocatenulatun*, were enriched in PD monkeys. In contrast, all the other differential species were enriched in NH monkeys. Especially multiple *Streptococcus* species (such as *Streptococcus lutetiensis, Streptococcus equinus*, and *Streptococcus gallolyticus*), *Butyrivibrio*/*Pseudobutyrivibrio* species (such as *Butyrivibrio hungatei*, *Butyrivibrio fibrisolvens*, *Butyrivibrio proteoclasticus* and *Pseudobutyrivibrio xylanivorans*), and *Clostridium* species showed decreased relative abundance in PD monkeys.

### Profiles and interaction of serum and fecal metabolome in PD monkeys

**Fig. 5:**
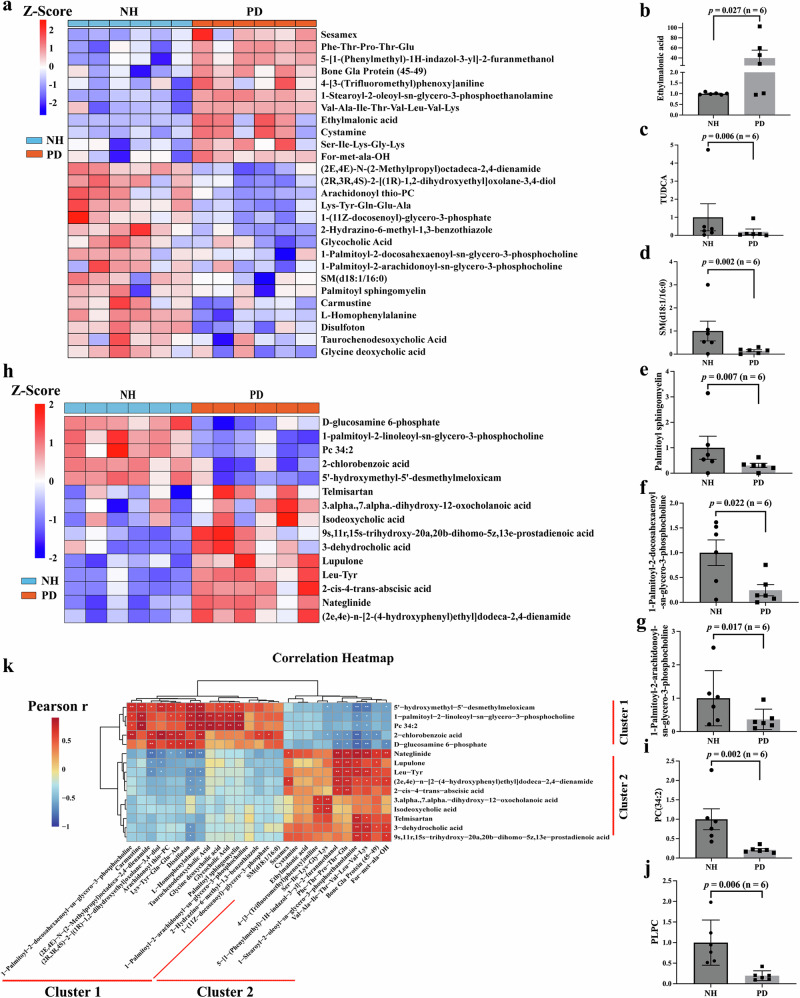
Differential serum and fecal metabolites in the PD monkeys. **a** The heatmap illustrating 27 differential metabolites. Blue and red indicate decreased and increased metabolites, respectively. **b**–**g** Levels of selected serum metabolites that significantly changed after MPTP treatment, shown as mean ± SEM. **h** Heatmap of 15 differential metabolites between NH and PD. Blue and red indicate decreased and increased metabolites, respectively. Correlation coefficients and *P*-values are calculated from Pearson correlation analysis. **i**, **j** Changes in select fecal metabolites in PD monkeys, shown as mean ± SEM. PC (34:2), phosphocholine (34:2); PLPC, 1-palmitoyl-2-linoleoyl-sn-glycero-3- phosphocholine. **k** Heatmap of Pearson correlation between serum and fecal metabolites in the PD monkeys. Differential metabolites are defined as those with empirical paired Student’s *t* test *p* < 0.05 and FDR < 0.2. NH neurologically healthy, PD Parkinson’s disease.

Metabolic changes have previously been reported in PD patients^9^. Untargeted serum metabolomic analysis identified a total of significantly altered 27 metabolites in PD monkeys with pair Student’s *t* test empirical *p* < 0.05, FDR < 0.2, and | log_2_FC| > 1 (Fig. 5a and Supplementary Fig. 3).

Noteworthy, among the metabolites with elevated serum levels in PD monkeys, ethylmalonic acid, which had been reported to disturb mitochondrial functions and induce oxidative stress^19^, dramatically increased by about 40-fold in PD monkeys (*p* = 0.027) (Fig. 5b). In contrast, serum tauro ursodeoxycholic acid (TUDCA) that had been previously reported to be potentially neuroprotective^20^ decreased significantly (*p* = 0.006) in PD monkeys (Fig. 5c). Most of the differential serum bioactive phospholipids in PD monkeys were down-regulated, including SM (d18:1/16:0) (*p* = 0.002, Fig. 5d), palmitoyl sphingomyelin (*p* = 0.007, Fig. 5e), 1-palmitoyl-2-docosahexaenoyl-sn-glycero-3- phosphocholine (PDPC) (*p* = 0.022, Fig. 5f), and 1-palmitoyl-2-arachidonoyl-sn-glycero-3-phosphocholine (PAPC) (*p* = 0.017, Fig. 5g).

Given the association between microbiota metabolism and GI function, untargeted metabolomic analyses of feces from PD monkeys were then analyzed. 15 fecal metabolites showed significant changes (empirical *p* < 0.05, FDR < 0.2, and |log_2_FC | > 1) in PD monkeys, with ten increased and five decreased significantly (Fig. 5h and Supplementary Fig. 4). Notably, several PC metabolites decreased in PD monkeys, such as PC (34:2) (Fig. 5i), 1-palmitoyl-2-linoleoyl-sn-glycero-3-phosphocholine (PLPC) (Fig. 5j).

Additionally, our correlation analysis results revealed distinct clusters in both serum and fecal metabolites (Supplementary Table 3), indicating a robust association between the two metabolomes. Most of the metabolites in serum Cluster 1 and fecal Cluster 1 showed significantly positive correlations with each other. Similarly, most of the metabolites in serum Cluster 2 and fecal Cluster 2 showed significantly positive correlations with each other. In contrast, only a limited number of metabolites in serum Cluster 1 and fecal Cluster 2 showed significantly negative correlations with each other and vice versa. (Fig. 5k). For example, fecal PLPC positively correlated with most metabolites in serum Cluster 1, such as PDPC and Palmitoyl sphingomyelin, whereas it negatively correlated with serum SOPE (1-stearoyl-2-oleoyl-sn-glycero-3-phosphoethanolanmine).

### The gut microbiota exhibited significant correlations with metabolites in both serum and feces

**Fig. 6:**
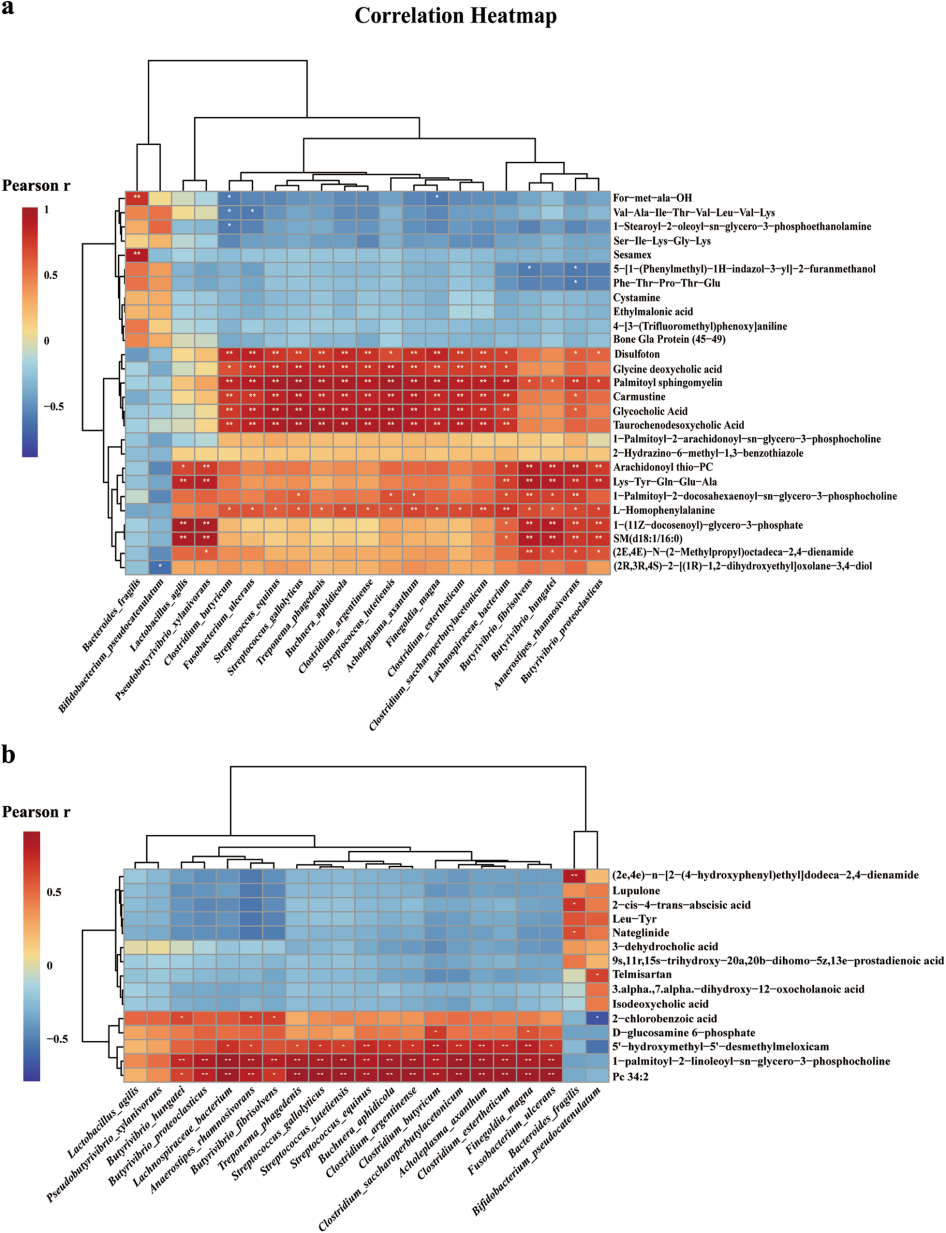
Heatmap of Pearson correlation between serum/fecal metabolites and species in gut microbiota in PD monkeys. Results are shown for serum metabolites (**a**)/fecal metabolites (**b**) and species Correlation coefficients and *P*-values are calculated from Pearson correlation analysis. *p < 0.05; **p < 0.01.

To better understand the role of the correlations between the gut microbiota and metabolites both in serum and feces, a Pearson correlation analysis was employed to identify representative differential gut microbiota shown in Fig. 6 and Supplementary Fig. 5, that correlated with differential serum (Supplementary Table 4) and fecal metabolites (Supplementary Table 5) in monkeys. At the genus level, the serum SM (d18:1/16:0) level was positively correlated with the relative abundance of *Butyrivibrio* (*r* = 0.915, *p* = 3.036 × 10^−5^) and *Pseudobutyrivibrio* (*r* = 0.926, *p* = 1.577 × 10^−5^) (Supplementary Fig. 5a). Both two genera belong to the Lachnospiraceae family of the Firmicutes phylum, also decreased in PD monkeys, pointing to the role of *Butyrivibrio* and *Pseudobutyrivibrio* in PD pathogenesis by regulating phospholipid metabolism. At the species level, the serum SM (d18:1/16:0) level was significantly positively correlated with *Butyrivibrio hungatei* (*r* = 0.882, *p* = 0.0001), *Butyrivibrio fibrisolvens* (*r* = 0.886, *p* = 0.0001), *Butyrivibrio proteoclasticus* (*r* = 0.772, *p* = 0.003) and *Pseudobutyrivibrio xylanivorans* (*r* = 0.926, *p* = 1.586 × 10^−5^) (Fig. 6a).

Furthermore, significant positive Pearson correlations between differential fecal metabolites and gut microbiota (Supplementary Table 5a, b). Glycerophosphocholines, including PC (34:2) and PLPC, were positively correlated with *Streptococcus* (PC (34:2), *r* = 0.915, *p* = 3.046E−05; PLPC, *r* = 0.874, *p* = 0.0002) and *Staphylococcus* (PC (34:2), *r* = 0.858, *p* = 0.0004; PLPC, *r* = 0.788, *p* = 0.002) (Supplementary Fig. 5b). In addition, at the species level, differential gut microbiota was positively correlated with these glycerophosphocholines (Fig. 6b), such as *Butyrivibrio fibrisolvens* (PC (34:2), *r* = 0.690, *p* = 0.013; PLPC, *r* = 0.782, *p* = 0.002), *Butyrivibrio proteoclasticus* (PC (34:2), *r* = 0.767, *p* = 0.004; PLPC, *r* = 0.830, *p* = 0.001) and several *Streptococcus* species.

### Multi-omic network highlighted the role of metabolites in host-microbiota interaction

**Fig. 7:**
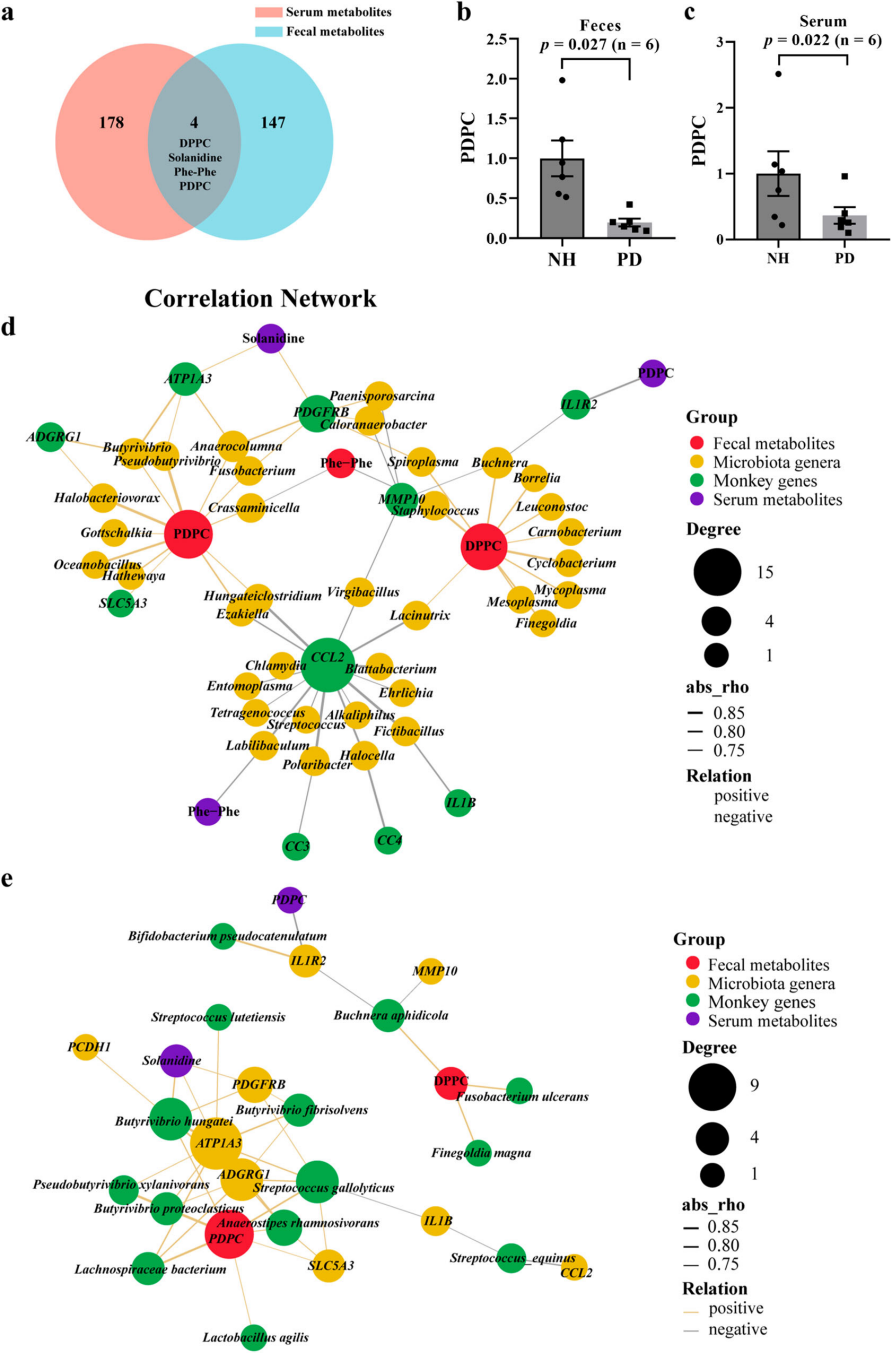
Correlation among PD-associated metabolites, gut microbiota, and host gene expression. **a** Venn diagram showing the overlap between serum and fecal differential metabolites (empirical *p* < 0.05, Pearson). Changes in fecal (**b**) and serum (**c**) PDPC between NH and PD, levels of PDPC shown as mean ± SEM. The multi-omic network of shared metabolites between fecal and serum, DEGs, and differential gut genera (**d**)/species (**e**) determin**e**d by LEfSe analysis. The node sizes represent the abundance of connected nodes. The yellow and gray lines represented positive and negative correlations, respectively (*p* < 0.05, |*r*| > 0.7). The node color represents the type of differential biomarkers. *P*-values are calculated from Pearson correlation analysis.

To further identify potential biomarkers of PD, differential metabolites common to serum and feces between PD and NH monkeys were analyzed using a multi-omic approach. Four of the differential metabolites with empirical *p* < 0.05 were shared between monkey serum and feces (Fig. 7a), including PC (16:0/16:0) (DPPC), Solanidine, Phe-Phe, and 1-palmitoyl-2-docosahexaenoyl-sn-glycero-3- phosphocholine (PDPC), respectively. Among the four metabolites, PDPC was the most significantly decreased in PD monkeys (fold change = 5.1 in feces and 2.7 in serum, respectively; Fig. 7b, c).

The Pearson correlation among the four metabolites, PD-associated DEGs (Fig. 3a), and representative differential gut microbiota (Fig. 4), was analyzed. The multi-omic interactive networks (Fig. 7d, e) identified PDPC as a hub metabolite correlated with PD-associated genes and gut microbes. Key genes in neuroprotection or neurogenesis, such as *ATP1A3, PDGFRB*, and especially *SLC5A3*, were positively correlated with fecal and serum PDPC levels (Feces: *r* = 0.8, *p* = 0.002; Serum: *r* = 0.78, *p* = 0.003; Supplementary Fig. 6a, b, Supplementary Table 3). Notably, PDPC also positively correlated with genus *Butyrivibrio* (Feces: *r* = 0.71, *p* = 0.010; Serum: *r* = 0.66, *p* = 0.019), and its species *Butyrivibrio hungatei* (Feces: *r* = 0.75, *p* = 0.005; Serum: *r* = 0.70, *p* = 0.012) and *Butyrivibrio fibrisolvens* (Feces: *r* = 0.77, *p* = 0.004; Serum: *r* = 0.73, *p* = 0.007), all significantly reduced in PD monkeys.

### PD-associated multi-omic biomarkers correlated with CRS scores of monkeys

**Fig. 8:**
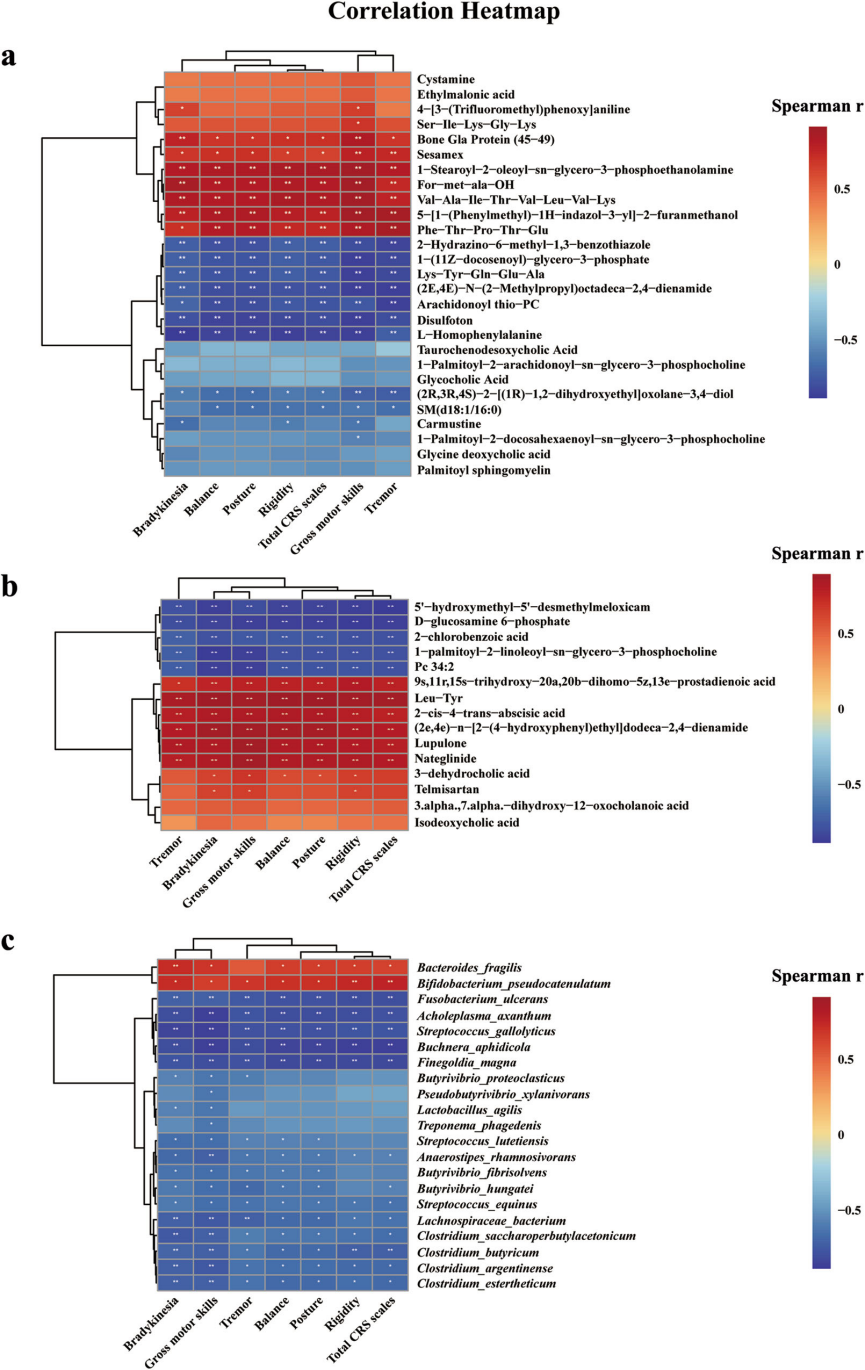
Spearman correlation between CRS scores and metabolites. Correlations in serum (**a**) and feces (**b**), as well as gut microbiota species (**c**). *P*-values are calculated from Spearman correlation analysis. *p < 0.05; **p < 0.01.

Furthermore, our results showed Spearman correlations between these PD-associated multi-omic biomarkers and the CRS scores of monkeys (Fig. 8a–c, Supplementary Figs. 7 and 8). In general, these biomarkers including genes, metabolites, and gut microbes that were significantly increased in PD monkeys exhibited a positive correlation with the CRS scores, especially gross motor skills, and vice versa. Importantly, the serum L-Homophenylalanine level was negatively correlated with CRS scores, whereas the serum PDPC level exhibited a more specific negative correlation with gross motor skill scores. The fecal D−glucosamine 6−phosphate level also negatively correlated with individual indices and the total score of CRS. *Bacteroides fragilis*, the most enriched species in PD monkeys, positively correlated with gross motor skills, whereas the most decreased in PD monkeys, especially several species of *Streptococcus*, *Butyrivibrio*, and *Clostridium* genera, negatively correlated with gross motor skills. Similarly, DEGs showed the most correlations with gross motor skills.

## Discussion

Gut microbiota plays a crucial role in the development of PD^21,22^. Therefore, understanding the interactions between gut microbes and the host is essential for comprehending the PD pathogenesis. By integrating multi-omic data from a monkey model that closely mimics clinical symptoms of Parkinson’s disease, our current study has highlighted the role of metabolite-mediated microbiota-host interaction and systematically identified potential biomarkers crucial for PD diagnosis and intervention.

The alpha diversity in PD patients was previously reported to be decreased in some previous studies but could be confounded by certain factors in the human populations, such as diet, antibiotics use, age, and gender^23^. Therefore, the alpha diversity changes in PD remains inconclusive before our study. It would be essential to control such confounding factors. Therefore, our NHP PD model could provide valuable insights into the role of gut microbiota in PD by effectively controlling potential confounding factors. Our results of the NHP PD model confirmed the observed decrease of alpha diversity among PD patients.

**Fig. 9:**
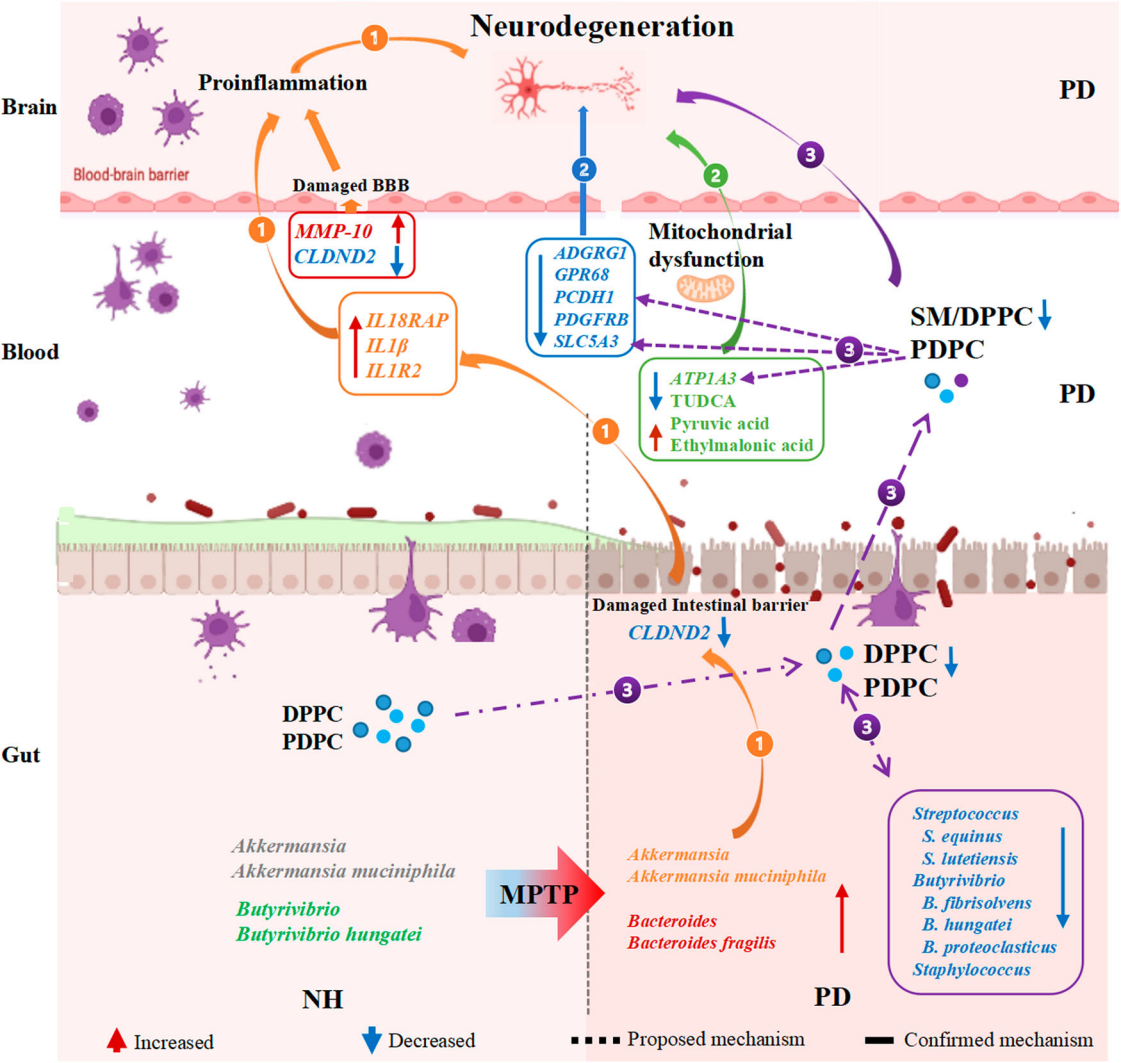
Proposed mechanisms of the multi-omic biomarkers and related pathways in PD pathogenesis based on existing literature and the current study. Pathway ① (orange solid line): Gut microbiota dysbiosis in the PD monkey model after MPTP treatment, which might result in a compromised intestinal barrier (indicated by down-regulated *CLDND2*) and is accompanied by inflammation (indicated by upregulated *IL18RAP*, *1IL1B*, *IL1R2*, and *MMP-10* in PBL). This might be involved in the BBB damage and eventually trigger neurodegeneration in PD. Pathway ② (solid blue line and solid green line): In the PBL of PD the monkeys, down-regulated DEGs such as *ADGRG1*, *GPR68*, *PCDH1*, *PDGFRB*, *SLC5A3*, and *ATP1A3*, could be linked to compromised neuroprotection, neurogenesis and promote mitochondrial dysfunction; abnormality of PD-associated metabolites such as TUDCA and ethylmalonic acid might also contribute to mitochondrial dysfunction and neurodegeneration. Pathway ③ (purple dashed line): In the PD monkey model, decreased commensals such as *Streptococcus*, *Butyrivibrio*, and *Staphylococcus*, positively correlated with reduced DPPC and PDPC levels, could be linked with inflammation, damaged lipid raft domains, mitochondrial dysfunction, and neurodegeneration through Pathways ① and ②. Solid lines indicate known regulatory mechanisms based on existing literature, whereas dashed lines indicate possible mechanisms proposed in our current study. The figure of proposed mechanisms is visualized by Figdraw.

Previous studies have shown that neuroinflammation is one of the key components in PD pathogenesis^24,25^. Proinflammatory cytokines and chemokines, such as *IL1*, were reported to be elevated in PD patients^24,25^. In line with this, proinflammatory factors, such as *CC4*, *IL18RAP*, and *IL1B*, in the PBL transcriptome were also elevated in PD monkeys (Fig. 9, pathway ①). Furthermore, the present study revealed downregulation of *CLDND2*, an intestinal barrier component, suggesting disruption of the blood-brain barrier (BBB) and neuroinflammation in PD monkeys. The BBB can protect neurons and is required for proper synaptic and neuronal functioning^26,27^. BBB disruption could be induced by neuroinflammation during PD development, which allows an influx of neurotoxic blood-derived debris, cells, and microbial pathogens into the brain, and causes inflammatory and immune responses in PD patients^26^. In addition, matrix metalloproteinase MMP-3 contributes to dopaminergic neurodegeneration, BBB damage, and neuroinflammation in PD development^28^. Our findings suggested that *MMP10*, a paralogue of the gene, may also have a similar role in patients with PD (Fig. 9, pathway ①). Furthermore, PD is characterized by the loss of dopaminergic neurons in the pars compacta of the substantia nigra^29^. Our transcriptomic data showed that several genes encoding neuroprotective proteins (GPCR, PDGFs/PDGFRs) highly expressed in dopaminergic neurons were down regulated in PD monkeys (Fig. 9, pathway ①). *ADGRG1* and *GPR68* are members of the aGPCR (adhesion G protein-coupled receptors) family and play an essential role in the brain’s normal development^30^. In addition, PDGFRB, an essential endogenous growth factor expressed in neuronal progenitors, neurons, astrocytes, and oligodendrocytes, could effectively promote neurogenesis^31,32^. The decreased blood expression of genes encoding neuroprotective proteins might correspond to impaired dopaminergic neuronal functions in PD (Fig. 9, pathway ①).

Mitochondrial dysfunction is a well-recognized cause of dopaminergic neuronal degeneration involved in PD development and progression^33^. Neurons are susceptible to mitochondrial challenges due to their high-level energy requirement with ATP as the primary energy source^34^. The a3-Na^+^/K^+^-ATPase, encoded by *ATP1A3*, is predominantly expressed in the central nervous system (CNS). *ATP1A3* mutations were associated with PD with acute and idiopathic dystonia or bradykinesia^35^. In line with its essential role, *ATP1A3* expression in PBL was found downregulated in PD monkeys in our current study (Fig. 9, pathway ②), suggesting that its PBL expression could be a potential biomarker for non-invasive PD diagnosis. Furthermore, serum level of TUDCA, a neuroprotective metabolite^20^, was found to be decreased in PD monkeys (Fig. 9, pathway ②), indicating mitochondrial dysfunction. TUDCA has been shown to possess the ability to prevent the loss of dopamine transporters, which are effective inhibitors of apoptosis through the mitochondrial pathway^20,36^.

Consistent with the changes in serum SM and other PCs, fecal glycerophosphocholines were significantly decreased in PD monkeys in our current study (Fig. 9, pathway ③), underlining a substantial of phospholipids in the disease. Phospholipids are essential components of the cellular plasma membrane and are crucial for cell function, brain development, and cognitive ability^37^. Changes in phospholipids might induce lipid raft rearrangement with the consequent development of neurological diseases^37^. In addition, PDPC, a component of the lipid raft domain in the cell plasma^38^, was significantly reduced in both serum and feces of PD monkeys (Fig. 9, pathway ③). Interestingly, it was demonstrated that both PDPC and SM positively correlated with gut microbes, including the genus *Butyrivibrio* and its species *B. hungatei* and *B. fibrisolvens*. In addition, PDPC positively correlated with *ATP1A3* and *SLC5A3* expression. Notably, DHA, the precursor of PDPC, was reported to exert neuroprotective effects in a rat PD model. Moreover, such differential metabolites, such as those reduced in both serum and feces, could be further transported from the gut into the blood and thereby mediate the microbe-host interactions. Therefore, our results suggested that these differential gut microbes, especially the commensals reduced in PD monkeys, could be involved in inflammation, damaged lipid raft domains, mitochondrial dysfunction, and neurodegeneration of Pathways ① and ② through their interplay with these biologically important metabolites.

Additionally, in our multi-omic analysis, differential metabolomic, transcriptomic, and metagenomic biomarkers in PD monkeys also showed strong correlations with the CRS scores in monkeys. CRS is a widely used tool for evaluating the severity of symptoms in individuals diagnosed with PD, with motor symptoms serving as fundamental phenotypes of PD. Interestingly, we observed a more evident correlation between these biomarkers and gross motor skills, further emphasizing their potential importance in PD.

In conclusion, our findings provided novel insights into understanding the potential role played by key metabolites in the host-microbiota interaction involved in PD pathogenesis. The multi-omic findings in the current study thus warranted further functional investigation and validation in subsequent studies.

## Methods

### Animals

The protocol for this study was approved by the Institutional Animal Care and Use Committee (IACUC) of Institute of Zoology, Guangdong Academy of Sciences (Approved No.: GIZ20210510). Six adults male cynomolgus monkeys (~12 years old) were sourced from Guangzhou Xiangguan Biotechnology company (Guangzhou, China), adhering to ARRIVE reporting guidelines. The health status of the monkeys was verified through comprehensive health records and a veterinary examination prior to experimentation. The monkeys were housed in a well-controlled and comfortable environment maintained at temperatures ranging between 16 and 26 °C, and relative humidity levels maintained between 30 and 70%, and subjected to a daily light-dark cycle consisting of 12 h each. Animals had access to food (formula feed and fresh vegetables/fruits) and water *ad libitum*.

### PD monkey model induction and clinical rating scale (CRS) scoring

After anesthetized through intramuscular injection of Zoletil 50 (0.1 ml/kg), monkeys were subcutaneously injected with MPTP (Sigma-Aldrich) at a dose of 0.1 mg/kg per day for four weeks using implanted ALZET osmotic pumps (DURECT Corporation, Cupertino, CA, USA). The implants were removed once overt PD symptoms (such as tremors, bradykinesia, and impaired balance) were observed persistently.

Double-blind analysis was used to evaluate the CRS scores based on PD symptoms in monkeys once weekly throughout the testing period before and after MPTP treatment^39^. The following indices were assessed and scored in the monkeys: tremor (absent, 0; mild/not persistently present, 1; moderate, 2; or severe, 3), bradykinesia (absent, 0; mild/not persistently present, 1; moderate, 2; or severe, 3), rigidity (absent, 0; mild/not persistently present, 1; moderate, 2; or severe, 3), posture (normal, 0; impaired, 1; frequent falling, 2; or no movement, 3), balance (absent, 0; mild/not always present, 1; moderate, 2; or severe, 3), and gross motor skills (normal, 0; slightly reduced, 1; reduced, 2; or no walking, 3). A total CRS score was calculated as the sum of the CRS indices for each monkey.

All the monkeys were determined as neurologically healthy (NH) with all CRS score values equal to 0 before MPTP treatment and confirmed as PD monkeys with total CRS scores no less than 8.

### Blood transcriptome sequencing and data analysis

Approximately 3 mL of fasting venous blood was collected from each monkey before (at Day 0, D0) and after MPTP treatment (at Day 30, D30) and immediately transferred into a PAXgene tube (BD Biosciences, San Jose) to stabilize RNA and avoid degradation. Total RNA was extracted using the TIANGEN RNA Simple Total RNA kit (DP419) and quantified with Qubit 3.0 Fluorometer (Thermo Fisher Scientific, Waltham), and RNA quality was evaluated with a 2100 BioAnalyzer (Agilent Technologies, Santa Clara). Samples with RNA integrity number (RIN) scores ≥ 7.0 were subjected to library construction using the polyA method and sequenced using the Illumina NovaSeq6000 platform (San Diego). FastQC was used for quality control of sequencing data^40^. After quality control, STAR (version 2.7.0e) was used to map sequencing reads against the reference genome (NCBI macFas 5.0)^41^. Pseudo counts were calculated using the featureCounts package^42^ and analyzed for differential gene expression using edgeR version 4.1 with a paired study design by incorporating the samples pairing information as a variable into the generalized linear model^43^. The transcripts per kilobase million (TPM) values were calculated to quantify gene expression levels. Differentially expressed genes (DEGs) were defined using a threshold of false discovery rate (FDR) < 0.05 and log of fold change (|log_2_FC|) > 1. Gene enrichment analysis, including Gene ontology (GO) and Kyoto Encyclopedia of Genes and Genomes (KEGG), was conducted, and enrichment scores for pathways were calculated using the ClueGO plugin version 2.5.10^44^ of Cytoscape version 3.9.1^45^, and visualized using the OECloud web tools (https://cloud.oebiotech.com).

### Reverse transcription and quantitative real-time PCR

Reverse transcription of RNA was conducted with HiScript III RT SuperMix plus gDNA wiper (Vazyme, Nanjing, China), according to the manufacturer’s instructions. The cDNA samples were then stored at −80 °C until quantitative real-time PCR (qPCR) was performed to quantify the relative mRNA expression levels of selected DEGs using the primers listed in Supplementary Table 6 and the ChamQ Universal SYBR qPCR Master Mix (Vazyme, Nanjing, China) on a Roche LightCycler 480 II (Roche, USA). Paired Wilcoxon signed-rank tests were used to analyze the qPCR validation data of selected genes.

### Metabolomic sample preparation and analysis

Fasting venous blood samples were collected from each monkey before (at D0) and after MPTP induction (at D30). Serum samples were transferred into sterile tubes after centrifugation at 3000 rpm for 5 min and stored at −80 °C until LC-MS analysis.

Fecal samples from each monkey were freshly collected before (at D0) and after MPTP treatment (at D30) into a 2 mL centrifuge tube. Samples were added with 600 µL MeOH (stored at −20 °C), vortexed for 30 s, homogenized, and centrifuged for 10 min (12,000 rpm, 4 °C). The supernatant was filtered using 0.22 μm membrane filters and stored at −80 °C before LC-MS analysis. On analysis, the supernatant was processed and subjected to LC-MS analysis using a Vanquish UHPLC System (ThermoFisher Scientific, Waltham). Mass spectrometric detection of metabolites was performed using Thermo Orbitrap Exploris 120 (ThermoFisher Scientific, Waltham) following instructions provided by the manufacturer (ThermoFisher Scientific, Waltham). The metabolomic raw data were converted to mzXML format by MSConvert in ProteoWizard software (v3.0.8789)^46^ and followed by processing using the XCMS version 3.12.0 of R for feature detection^47^, retention time correction and alignment. The processed metabolomic data was then corrected using the area normalization method, and its log_10_ transformed values were used in the paired Student’s *t* test for differential metabolite analysis.

### Metagenomic sequencing and data analysis

Rectal swab samples were freshly collected from each monkey before (at D0) and after MPTP treatment (at D30) and stored at −80 °C immediately until DNA extraction. Microbial DNA was extracted using the TIANamp Stool DNA kit (Catalog No. DP328-02, Tiangen, Beijing, China) according to the manufacturer’s instructions. All extracted DNA samples were stored at −80 °C for subsequent experiments.

Each fecal DNA sample was used to construct libraries with an average inserted fragment size of 300 bp and sequenced using a paired-end (2 ×150 bp) configuration on the Illumina NovaSeq6000 (Illumina, California) platform. The resulting data were filtered against the host genomic sequence (NCBI assembly MacFas 5.0) using KneadData (https://github.com/biobakery/kneaddata, version v0.7.4), with the clean data amount per sample ≥6 G. Kraken2 was then used for the taxonomic analysis of metagenome data^48^. The relative abundance of microbes was then determined by normalizing the raw counts of individual microbes with the total counts of taxa calculated by Kraken2. The Linear discriminant analysis Effect Size (LEfSe) method with a log-linear discriminant analysis (LDA) score cutoff of 2 was used to identify differentially abundant taxa enriched in groups^49^. Considering that the LEfSe method did not account for sample pairing, a paired likelihood ratio test with the generalized linear model (GLM) method implemented by edgeR was conducted to confirm differential gut microbes detected by the LEfSe analysis. Alpha-diversity indices were calculated using the picante and vegan packages of R, visualized using GraphPad Prism v.9.0 (Boston, Massachusetts), and compared between groups using the Wilcoxon signed-rank test. Permutational multivariate analysis of variance (PERMANOVA) based on Bray–Curtis distances was performed for beta-diversity using the vegan package.

### Statistical analysis

Statistical analysis was performed using GraphPad Prism and the R statistical language. The Benjamini-Hochberg FDR correction for multiple testing was applied to analyze the significance of differentially expressed genes, metabolites, gut microbes, and gene function enrichment, except for LEfSe. Pearson correlation was used to analyze the correlation among gut microbes, metabolites, and genes, the Spearman method was employed to analyze the correlation between CRS scores and omics data, given that the CRS scores of monkeys were ranked data that deviated from a normal distribution. Correlation coefficients (*r*) and *P*-values were calculated accordingly. The interactive networks between biomarkers with |*r*| > 0.6 were constructed and visualized using the OECloud web tools (https://cloud.oebiotech.com). The Venn and volcano plots were generated using the ggplot2 package of R, and heatmap plots were generated using the OECloud web tools, in which Z-scores were applied to transform the relative abundance of microbes and metabolites and TPM values of gene expression.

### Reporting summary

Further information on research design is available in the Nature Research Reporting Summary linked to this article.

### Supplementary information

SUPPLEMENTAL MATERIAL

Reporting summary

## Data Availability

The raw data of transcriptome and metagenome sequencing in the current study have been deposited in the Genome Sequence Archive at the National Genomics Data Center, Beijing Institute of Genomics, Chinese Academy of Sciences/China National Center for Bioinformation (GSA: CRA010066 and CRA010067) and are publicly accessible at https://ngdc.cncb.ac.cn/gsa. Metabolomics data have been deposited in MetaboLights and are publicly accessible at https://www.ebi.ac.uk/metabolights/MTBLS10496.
