# Supplementary material for: Multiomics of parkinsonism cynomolgus monkeys highlights significance of metabolites in interaction between host and microbiota
Source: NPJ Biofilms Microbiomes. 2024 Jul 26;10:61. doi: 10.1038/s41522-024-00535-3 (PMC11282307; doi:10.1038/s41522-024-00535-3)
Supplement: Supplementary file 1 — SUPPLEMENTAL MATERIAL [file 41522_2024_535_MOESM1_ESM.pdf]

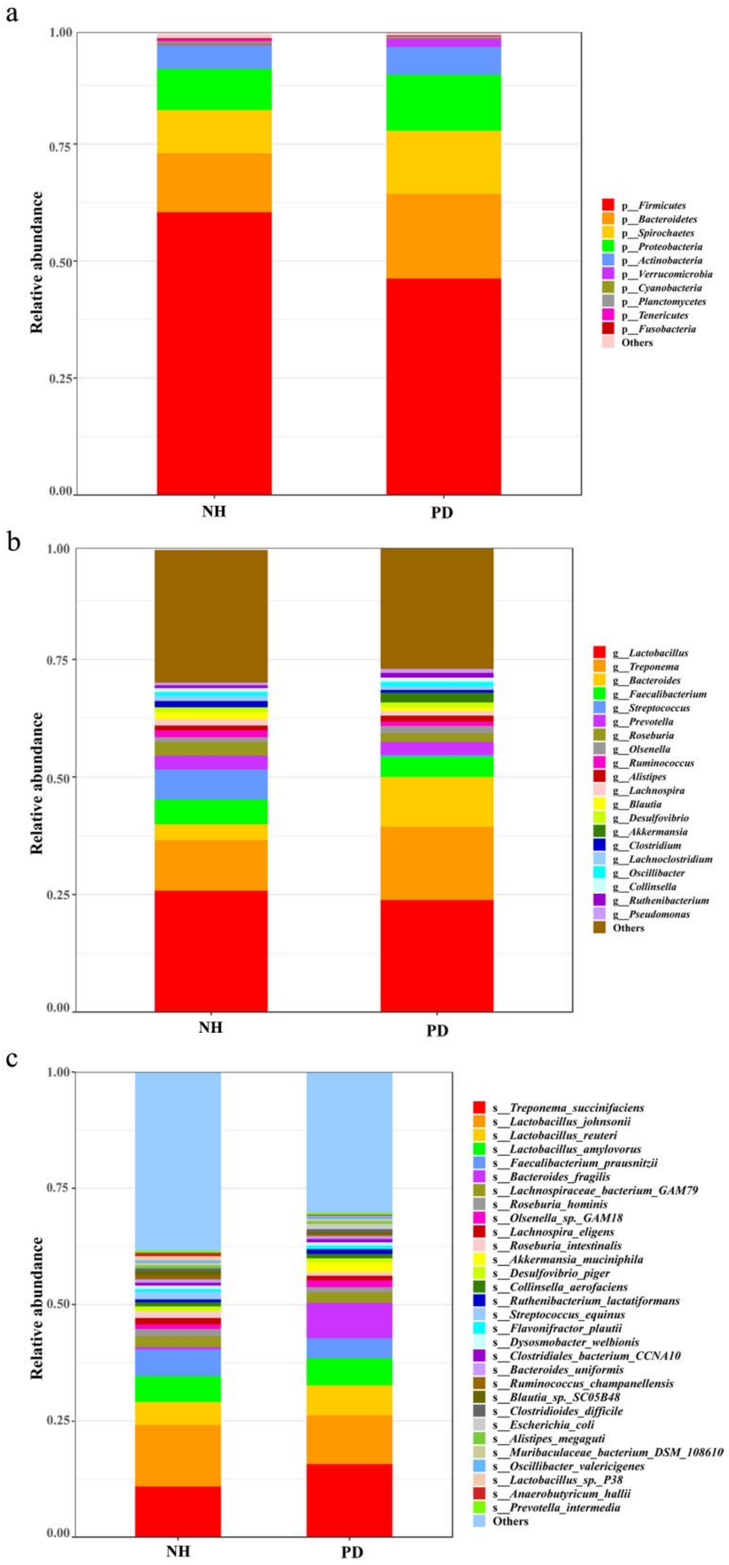

Supplementary Figure 1. The most abundant phyla, genera, and species in the gut microbiota in NH and PD monkeys. The top 10 phyla (a), top 20 genera (b), and top 30 species (c) observed in the gut microbiota in NH and PD monkeys. NH, neurologically healthy; PD, Parkinson's disease.

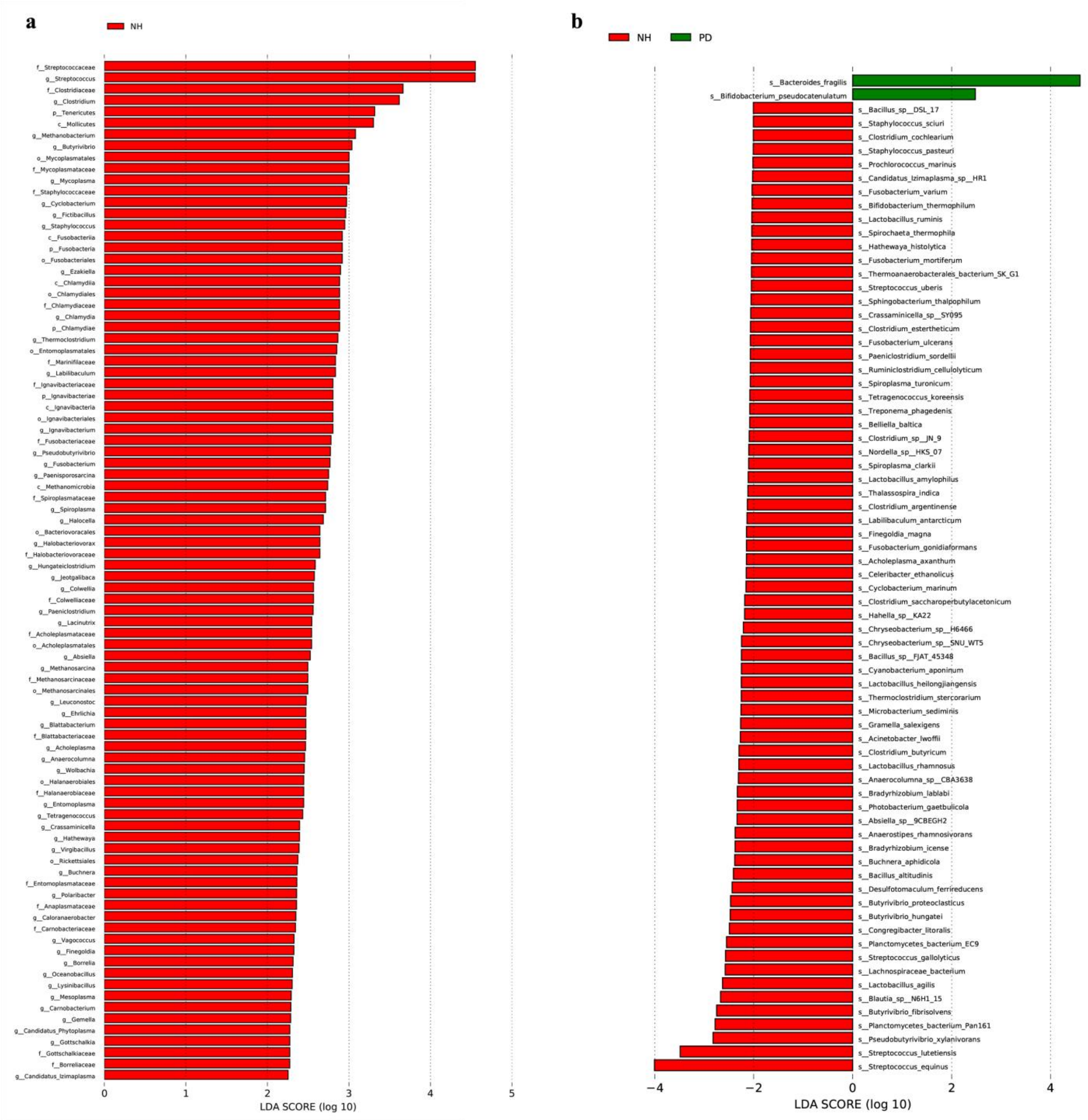

Supplementary Figure 2. LEfSe results showing dramatic changes in the gut microbiota in PD monkeys. Results are shown for the full list of differential gut microbiota from the phylum to genus levels (**a**) and species level (**b**). NH, neurologically healthy; PD, Parkinson's disease. Statistical significance is determined by LEfSe analysis with $p < 0.05$ and LDA > 2.0.

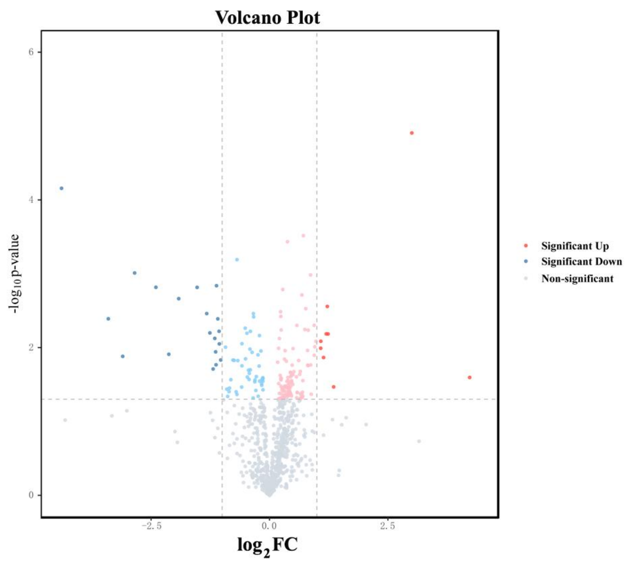

Supplementary Figure 3. Volcano plot illustrating changes in serum metabolites in monkeys after MPTP treatment. Blue and red dots represent decreased and increased metabolites with $p < 0.05$, respectively. Grey dots denote metabolites with no significant alterations. *P-values* are calculated from paired Student's t-test.

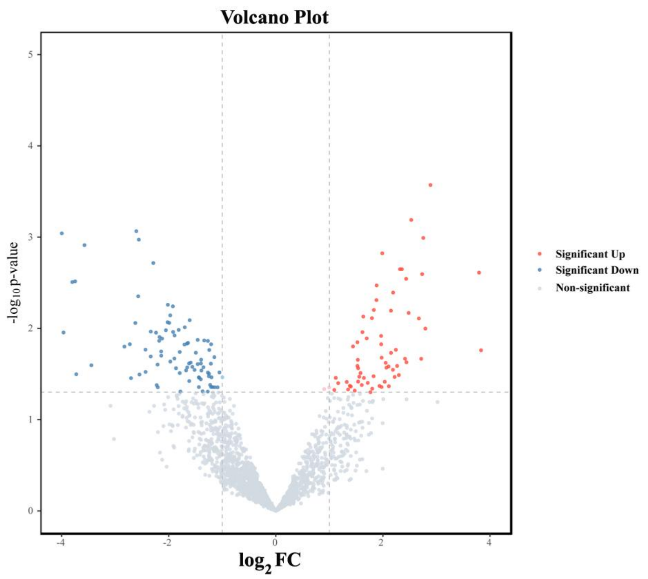

Supplementary Figure 4. Volcano plot illustrating changes in fecal metabolites in monkeys after MPTP treatment. Blue and red dots represent decreased and increased metabolites with $p < 0.05$, respectively. Grey dots denote metabolites with non-significant alterations. *P-values* are calculated from paired Student's *t*-test.

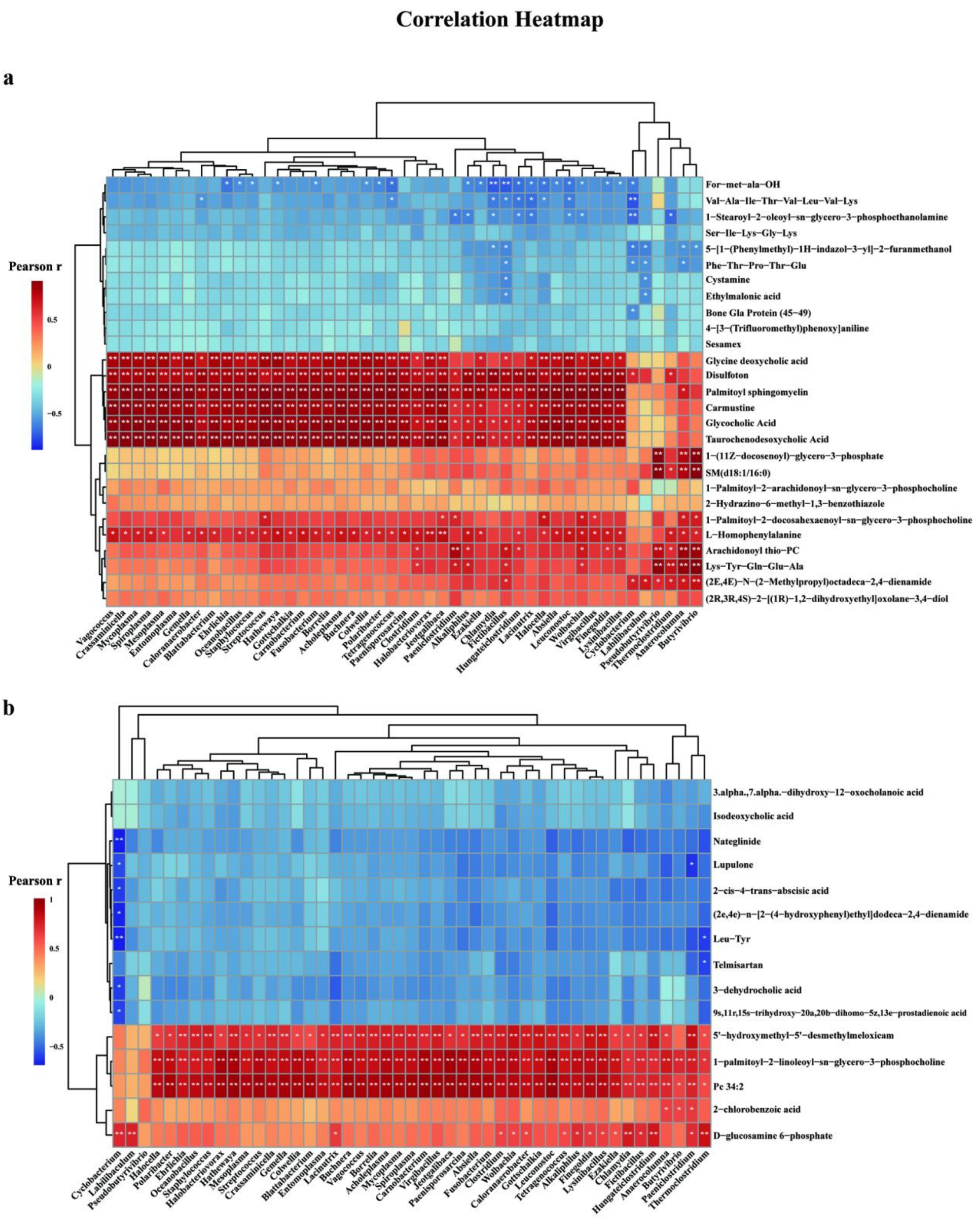

Supplementary Figure 5. Heatmap of Pearson correlation analysis between metabolites and gut microbiota genera in monkeys. a. *Pearson* correlation between serum metabolites and gut microbiota genera. b. *Pearson* correlation between fecal metabolites and gut microbiota genera. *, $p < 0.05$; **, $p < 0.01$.

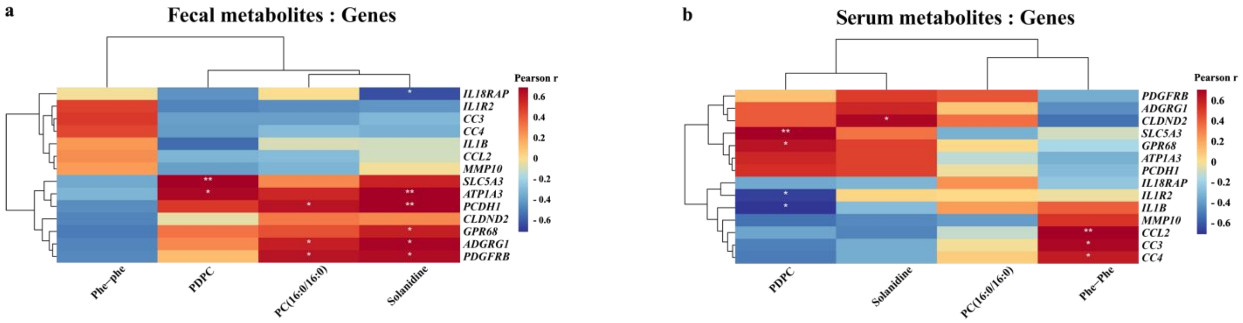

Supplementary Figure 6. Heatmap illustrating Pearson correlation among DEGs and shared differential metabolites both in serum and feces. (a) Feces, and (b) Serum. *, $p < 0.05$; **, $p < 0.01$. DEGs, differentially expressed genes.

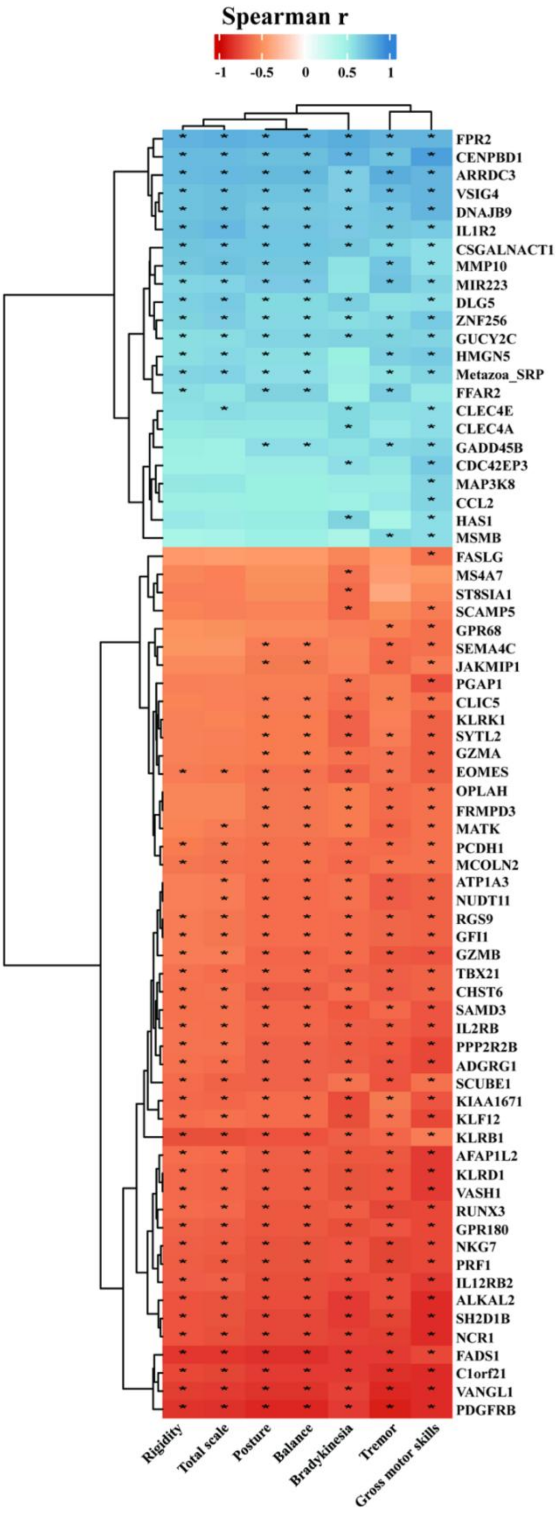

Supplementary Figure 7. Heatmap illustrating Spearman correlations between DEGs and CRS scores of monkeys. *, $p < 0.05$; **, $p < 0.01$. DEGs, differentially expressed genes; CRS, clinical rating scale.

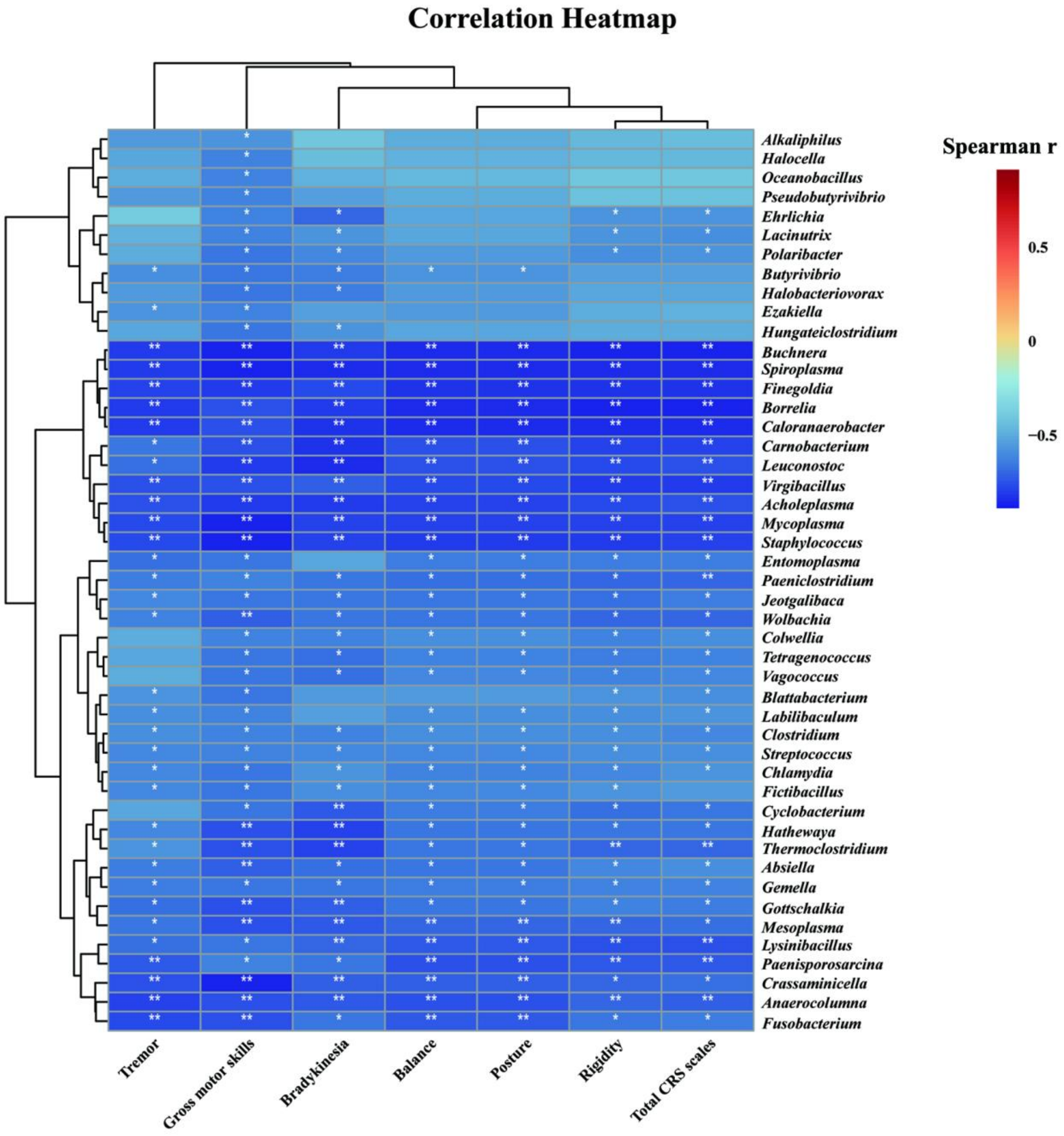

Supplementary Figure 8. Heatmap showing Spearman correlation between differential gut microbiota genera and CRS scores of monkeys. *, $p < 0.05$; **, $p < 0.01$. DEGs, differentially expressed genes; CRS, clinical rating scale.

Supplementary Table 1a No. of reads of RNA-Seq analysis of PBL								
Samples	raw_reads	raw_bases	clean_reads	clean_bases	error_rate	Q20	Q30	GC_pct
NH1	48188114	7.23G	45526182	6.83G	0.03	97.62	93.38	50.7
NH2	46347550	6.95G	43586034	6.54G	0.03	97.39	92.92	51.12
NH3	39226242	5.88G	39183286	5.88G	0.03	97.74	93.59	45.83
NH4	45732162	6.86G	42446030	6.37G	0.03	97.46	92.99	51.25
NH5	45486746	6.82G	41974150	6.3G	0.03	97.77	93.77	53.09
NH6	36032376	5.4G	35982594	5.4G	0.03	97.63	93.4	49.84
PD1	45620804	6.84G	41722432	6.26G	0.03	97.61	93.4	52.07
PD2	42999192	6.45G	39918322	5.99G	0.03	97.63	93.36	49.45
PD3	47564178	7.13G	45318310	6.8G	0.03	97.57	93.15	47.16
PD4	45947142	6.89G	44333206	6.65G	0.03	97.5	93.01	49.09
PD5	47331626	7.1G	44419858	6.66G	0.03	97.64	93.44	47.69
PD6	50535190	7.58G	47616692	7.14G	0.03	97.52	93.1	51.57

Supplementary Table 1b Differentially expressed genes					
Gene ID	Gene name	logFC	logCPM	*P* Value	FDR
ENSMFAG00000043935	CCL2	3.935090467	2.419499722	3.30E-14	4.55E-10
ENSMFAG00000009219	RF00017	2.303138177	-0.949406318	0.0001037	0.010883846
ENSMFAG00000047023		2.16268961	-0.792782225	0.001004611	0.047447211
ENSMFAG00000045255		2.052607972	6.783391114	0.000110587	0.011255641
ENSMFAG00000031319		1.972371309	-0.390028413	0.000380581	0.024641301
ENSMFAG00000032685	CA4	1.918181951	3.096215579	2.28E-08	2.86E-05
ENSMFAG00000003696		1.85660435	0.009994812	0.000497161	0.028808188
ENSMFAG00000040009	NANOS3	1.846973207	-0.709549673	0.000399122	0.02513375
ENSMFAG00000041454	CLEC4D	1.822348715	4.820929192	1.39E-06	0.000434917
ENSMFAG00000034337	MSMB	1.7954951	-0.111281463	0.000293239	0.02196947
ENSMFAG00000044821		1.756568132	-0.923720772	0.001032693	0.047952409
ENSMFAG00000032719		1.655350842	6.872083307	3.70E-07	0.000159593
ENSMFAG00000043720		1.578344022	1.003251732	0.000354986	0.024275262
ENSMFAG00000032590	EFHC2	1.505296122	3.133311465	2.53E-05	0.004110981
ENSMFAG00000031795	MMP10	1.471824438	2.18566687	1.61E-05	0.002921237
ENSMFAG00000033846		1.456586514	4.168270031	0.000101967	0.01081716
ENSMFAG00000002284	DNAJB9	1.40561853	4.704407729	0.000185219	0.01576762
ENSMFAG00000043760		1.392627559	1.12099889	0.000714492	0.037393139
ENSMFAG00000036514		1.373549046	8.333147342	4.53E-05	0.006445588
ENSMFAG00000040863	PTX3	1.372683629	4.855335988	5.97E-05	0.007921062
ENSMFAG00000030788		1.370515086	0.44578041	0.000238391	0.019226038
ENSMFAG00000044905	CXCL3	1.340777217	3.191877999	0.00030485	0.022207699
ENSMFAG00000043038	IL18RAP	1.325421118	4.182285206	3.15E-08	3.07E-05
ENSMFAG00000044509	HAS1	1.319499397	4.979112888	3.85E-06	0.001040439
ENSMFAG00000031450	ANPEP	1.310180152	3.63517496	3.20E-07	0.000142464
ENSMFAG00000003333		1.306285623	1.04043414	0.000355565	0.024275262
ENSMFAG00000005127	CXCL3	1.303378758	3.126439013	0.000810397	0.041240546
ENSMFAG00000044330	CLEC4E	1.283401568	6.43570386	6.65E-06	0.001580361
ENSMFAG00000035180	PDK4	1.278457485	5.602761612	0.000377148	0.024641301
ENSMFAG00000029757	CCM2L	1.27456338	2.686125231	8.99E-07	0.000309793
ENSMFAG00000043155	HMGN5	1.263779103	4.102716793	0.000297897	0.02196947
ENSMFAG00000014850	CDC42EP3	1.250820822	6.297029329	7.52E-09	1.73E-05
ENSMFAG00000043042	INHBB	1.249768546	3.18403297	3.28E-05	0.005136547
ENSMFAG00000028229		1.248700403	6.690748471	0.000125725	0.011982387
ENSMFAG00000030727	FPR2	1.247717271	4.831098844	1.51E-08	2.60E-05
ENSMFAG00000031762	ARRDC3	1.233462431	7.292644158	1.16E-05	0.002221424
ENSMFAG00000022604	FFAR2	1.22501331	4.031115307	1.35E-05	0.00255536
ENSMFAG00000035497	PFKFB3	1.205486634	8.985050555	2.01E-05	0.003425572
ENSMFAG00000002320	GUCY2C	1.204085939	2.885633333	3.64E-05	0.005578838
ENSMFAG00000032420	HES1	1.194559569	3.702651052	0.000216874	0.017909668
ENSMFAG00000042254	GADD45B	1.185049543	6.207069803	1.03E-05	0.002084854
ENSMFAG00000032130	CENPBD1	1.177002533	3.939109338	2.48E-07	0.000117927
ENSMFAG00000032038	DLG5	1.173721682	4.134188972	7.13E-08	5.15E-05
ENSMFAG00000045019	CSGALNACT	1.161837847	5.135607161	8.60E-08	5.15E-05
ENSMFAG00000001866	VSIG4	1.154065691	2.229855521	0.000450922	0.026920606
ENSMFAG00000039366	FAM89A	1.147813598	4.376902366	7.58E-05	0.009256081

ENSMFAG00000046196	IL1R2	1.145196719	6.968437993	1.45E-05	0.002695603
ENSMFAG00000031381	IL1RN	1.14137513	6.602235053	0.000301072	0.022085584
ENSMFAG00000000319	MAFF	1.141271041	3.891498982	0.000230823	0.018835998
ENSMFAG00000045130	S100A9	1.134773006	11.31503373	9.73E-05	0.010649281
ENSMFAG00000039568	S100A8	1.12911488	9.939741706	2.17E-07	0.000107036
ENSMFAG00000040572		1.126310417	6.461726211	0.000181209	0.015667498
ENSMFAG00000043064	IL1B	1.119174173	6.64908832	4.17E-06	0.001106447
ENSMFAG00000034188	ITLN1	1.115058474	2.66008066	4.06E-05	0.006079204
ENSMFAG00000010580	MIR223	1.080284535	5.138276697	5.91E-07	0.000220313
ENSMFAG00000025652	BTG2	1.076352975	10.37228643	0.000383406	0.024708177
ENSMFAG00000033126		1.074726367	6.096115119	0.000282179	0.021500134
ENSMFAG00000000313		1.070252058	1.974301729	0.000983677	0.046618199
ENSMFAG00000025573		1.069607141	4.567439356	1.81E-06	0.000542573
ENSMFAG00000033109	MGST1	1.069510349	1.669174391	0.000340033	0.024040315
ENSMFAG00000037310	SGK1	1.063771374	7.43158947	0.000161999	0.01423009
ENSMFAG00000046631		1.062863826	3.205312976	0.000257152	0.020265034
ENSMFAG00000039828		1.058405985	4.187439848	3.03E-06	0.00087151
ENSMFAG00000031850	TUB	1.056814304	5.566301121	1.14E-05	0.002209943
ENSMFAG00000033738	N4BP3	1.054758116	5.854905229	9.84E-06	0.002055888
ENSMFAG00000035171		1.045219474	3.680232927	0.000114769	0.011469377
ENSMFAG00000041299		1.045036099	8.67851884	0.000412842	0.025646415
ENSMFAG00000024958		1.035102467	3.459956395	0.000526668	0.02967195
ENSMFAG00000039015	PDE6H	1.030766002	4.454332618	0.00049267	0.028668387
ENSMFAG00000028216		1.021901086	4.343570724	0.000506965	0.028993835
ENSMFAG00000036426	CLEC4A	1.017406225	6.694378993	0.000111619	0.011255641
ENSMFAG00000041471	IDO2	1.008659231	4.222905627	0.000333267	0.023813931
ENSMFAG00000032589	ZNF256	1.008209087	3.185034794	0.00014745	0.013647564
ENSMFAG00000026375		1.008003305	1.535111726	0.000484318	0.028317709
ENSMFAG00000031229	SLC26A8	1.005190884	2.993307529	0.000419509	0.025827882
ENSMFAG00000029021		1.001515633	4.296431353	2.35E-05	0.003850393
ENSMFAG00000030792		-1.001311532	3.194261063	2.20E-05	0.003655489
ENSMFAG00000002012	EOMES	-1.004149882	4.08984119	4.42E-06	0.001149946
ENSMFAG00000034187	FRMPD3	-1.004780939	2.126036749	9.94E-05	0.010713815
ENSMFAG00000031725	RUNX3	-1.00692944	7.558257217	1.47E-05	0.002711271
ENSMFAG00000041776	PDE4D	-1.012411728	4.117849259	1.04E-05	0.002084854
ENSMFAG00000024087		-1.060744069	5.621953117	0.0003889	0.024787814
ENSMFAG00000030470	MATK	-1.061947997	4.860800984	0.000389765	0.024787814
ENSMFAG00000038771	C1QC	-1.068196493	3.817625025	9.13E-05	0.010273575
ENSMFAG00000038267	GPR153	-1.069011679	5.461784149	0.000324808	0.023381732
ENSMFAG00000043992	GPR180	-1.06913698	0.941666908	0.000428823	0.026167678
ENSMFAG00000042905	MS4A7	-1.082470508	4.962037207	5.43E-05	0.007410091
ENSMFAG00000002684	KLF12	-1.084456798	2.91445446	2.01E-05	0.003425572
ENSMFAG00000045666	SEMA4C	-1.09489633	1.711539484	7.65E-05	0.009257224
ENSMFAG00000042675	ST8SIA1	-1.105413285	1.873981512	0.00011819	0.011559946
ENSMFAG00000002016	OPLAH	-1.108212666	4.451024989	5.85E-05	0.007837046
ENSMFAG00000032887		-1.113815938	4.987935771	1.49E-07	8.20E-05
ENSMFAG00000037025	FADS1	-1.117787463	1.107537392	0.000111814	0.011255641
ENSMFAG00000039452	MYRF	-1.126235331	5.340968763	7.49E-06	0.001666423

ENSMFAG00000018638	HSPH1	-1.129664021	5.474533164	8.75E-05	0.010059439
ENSMFAG00000032496	KLRK1	-1.136577882	4.362874748	1.60E-07	8.48E-05
ENSMFAG00000031312	MCOLN2	-1.151224959	3.250102366	5.92E-06	0.001431125
ENSMFAG00000036205	JAKMIP1	-1.169317526	3.425949498	1.76E-05	0.003112699
ENSMFAG00000005042	CLIC5	-1.174487686	4.021833146	3.92E-07	0.000163832
ENSMFAG00000002554	HEG1	-1.175391384	4.412675194	0.000154024	0.014160937
ENSMFAG00000033087	GPT2	-1.179811643	2.598294172	0.000295219	0.02196947
ENSMFAG00000038071	SAMD3	-1.18232055	2.613793764	8.28E-06	0.001813209
ENSMFAG00000001630	GALNT9	-1.183630139	1.582166117	0.00067938	0.035897819
ENSMFAG00000045801	GFI1	-1.200417914	3.609272893	1.15E-06	0.000378144
ENSMFAG00000033230	SCAMP5	-1.20370993	1.226186165	0.000955375	0.045590253
ENSMFAG00000002723	CMA1	-1.212209906	2.848404693	6.77E-05	0.00880783
ENSMFAG00000002223	ADGRG1	-1.212862795	5.313024347	2.12E-06	0.000622476
ENSMFAG00000039338	FASLG	-1.218809014	2.274888466	8.60E-05	0.010037838
ENSMFAG00000035997	SCUBE3	-1.219654003	2.472826897	4.47E-05	0.006445588
ENSMFAG00000016132		-1.22509666	3.0334394	4.98E-06	0.001247599
ENSMFAG00000037849		-1.232643225	1.794048006	9.72E-06	0.002055888
ENSMFAG00000029122	PGAP1	-1.238100811	1.257707084	0.000295411	0.02196947
ENSMFAG00000044542	ENPP4	-1.241792781	4.16418474	2.88E-08	3.07E-05
ENSMFAG00000044139	KIAA1671	-1.2428069	2.662196226	1.10E-05	0.002170337
ENSMFAG00000011873	CHST6	-1.257195755	2.337781848	1.36E-06	0.000434917
ENSMFAG00000042504	TBX21	-1.274894963	5.273283877	4.44E-07	0.000175094
ENSMFAG00000039925	KLRB1	-1.322660839	3.988903226	4.34E-07	0.000175094
ENSMFAG00000039153	GZMA	-1.328934095	3.589978592	6.81E-06	0.001591131
ENSMFAG00000010284	RF01874	-1.342059425	1.154712861	7.36E-05	0.009088411
ENSMFAG00000001286	KLRD1	-1.344101807	5.094450767	1.50E-08	2.60E-05
ENSMFAG00000032260	VASH1	-1.346381263	2.310680644	0.00014291	0.013316729
ENSMFAG00000044391		-1.381152439	2.876348075	3.10E-06	0.00087151
ENSMFAG00000035342	NKG7	-1.382787103	6.578026976	1.76E-06	0.000539625
ENSMFAG00000033010	PRF1	-1.386652407	7.059212754	8.13E-08	5.15E-05
ENSMFAG00000000860	VANGL1	-1.38722965	3.340575687	5.59E-09	1.54E-05
ENSMFAG00000004079	GNLY	-1.388034444	6.918206709	1.25E-07	7.16E-05
ENSMFAG00000002611	PPP2R2B	-1.390872148	1.856717574	1.04E-06	0.000350758
ENSMFAG00000044133	NCR1	-1.410084364	2.953371516	6.08E-07	0.000220747
ENSMFAG00000039087	NUDT11	-1.415283829	1.605487791	1.02E-05	0.002084854
ENSMFAG00000002576	IL2RB	-1.417383004	7.075022139	5.49E-08	4.56E-05
ENSMFAG00000037875		-1.448206932	2.883410888	1.78E-08	2.70E-05
ENSMFAG00000004536	GPR68	-1.45292215	2.957809541	0.000125984	0.011982387
ENSMFAG00000043077	GZMB	-1.462040725	8.308267517	8.56E-08	5.15E-05
ENSMFAG00000026094	SCUBE1	-1.46657261	4.557832017	8.66E-05	0.010037838
ENSMFAG00000009737		-1.473911146	2.484982714	2.88E-05	0.004605157
ENSMFAG00000033157		-1.509605484	-0.003479917	0.000457232	0.027179681
ENSMFAG00000035462	RGS9	-1.543482708	4.924016511	1.64E-11	5.65E-08
ENSMFAG00000038653		-1.567805934	-0.251388464	0.00094481	0.045242622
ENSMFAG00000044595	IL12RB2	-1.568967068	2.787861454	3.34E-08	3.07E-05
ENSMFAG00000039792	CLDND2	-1.587922751	1.788666483	7.00E-05	0.009026702
ENSMFAG00000002810	AFAP1L2	-1.641353576	4.277102259	2.90E-12	1.33E-08
ENSMFAG00000035695	PCDH1	-1.669956911	4.291200289	2.89E-08	3.07E-05

ENSMFAG00000044787		-1.691367337	4.160047585	7.92E-08	5.15E-05
ENSMFAG00000019833	SLC5A3	-1.707326988	2.119273719	5.62E-08	4.56E-05
ENSMFAG00000000209		-1.716342995	2.652684607	7.75E-07	0.000274088
ENSMFAG00000030701		-1.762190666	2.279474505	3.12E-07	0.000142464
ENSMFAG00000002126	RXRG	-1.764924668	1.056426886	3.61E-06	0.000996045
ENSMFAG00000044846		-1.783947329	0.252620143	0.000117035	0.011532496
ENSMFAG00000002098		-1.868537326	2.614524552	1.79E-07	9.13E-05
ENSMFAG00000001789	ALKAL2	-1.871226701	0.78708294	5.87E-07	0.000220313
ENSMFAG00000040797	SH2D1B	-1.908242801	2.020497407	1.96E-08	2.70E-05
ENSMFAG00000001633	PDGFRB	-2.115986252	4.86772618	1.53E-12	1.06E-08
ENSMFAG00000024353		-2.157618444	0.530988102	1.86E-05	0.003252167
ENSMFAG00000045835	C1orf21	-2.260512038	0.056877998	7.54E-08	5.15E-05

Supplementary Table 2 Summary of metagenomic sequencing reads							
Samples	InsertSize(bp)	RawData	CleanData	Clean_Q20	Clean_Q30	Clean_GC(%)	Effective(%)
NH1	350	12,843.38	12,829.43	97.59	93.25	46.83	99.891
NH2	350	12,524.02	12,513.54	97.66	93.36	45.16	99.916
NH3	350	12,568.35	12,559.93	97.72	93.45	41.70	99.933
NH4	350	13,192.04	13,183.22	97.69	93.47	44.21	99.933
NH5	350	12,247.77	12,232.04	97.64	93.48	45.94	99.872
NH6	350	12,881.24	12,846.27	97.69	93.64	47.97	99.729
PD1	350	12,505.35	12,480.96	97.66	93.47	46.39	99.805
PD2	350	12,863.03	12,804.00	97.59	93.44	49.52	99.541
PD3	350	13,034.91	13,010.31	97.26	92.54	48.62	99.811
PD4	350	12,833.13	12,785.84	97.78	93.75	46.28	99.632
PD5	350	12,559.69	12,541.73	97.66	93.58	48.50	99.857
PD6	350	12,447.05	12,392.36	97.72	93.69	47.69	99.561

Supplementary Table 3 Pearson correlation between serum metabolites and fecal metabolites in cynomolgus monkeys			
fecal metabolites	serum metabolites	*r*	*p*
PC 34:2	Carmustine	0.89	0.0001
PC 34:2	Palmitoyl sphingomyelin	0.88	0.0002
1-palmitoyl-2-linoleoyl-sn-glycero-3-phosphocholine	L-Homophenylalanine	0.88	0.0002
PC 34:2	Taurochenodesoxycholic Acid	0.87	0.0002
2-chlorobenzoic acid	(2R,3R,4S)-2-[(1R)-1,2-dihydroxyethyl]oxolane-3,4-diol	0.87	0.0003
Nateglinide	1-Stearoyl-2-oleoyl-sn-glycero-3-phosphoethanolamine	0.86	0.0004
PC 34:2	Glycocholic Acid	0.85	0.0005
1-palmitoyl-2-linoleoyl-sn-glycero-3-phosphocholine	Carmustine	0.84	0.0006
PC 34:2	L-Homophenylalanine	0.84	0.0007
Nateglinide	5-[1-(Phenylmethyl)-1H-indazol-3-yl]-2-furanmethanol	0.83	0.0008
1-palmitoyl-2-linoleoyl-sn-glycero-3-phosphocholine	Palmitoyl sphingomyelin	0.83	0.0009
5'-hydroxymethyl-5'-desmethylmeloxicam	L-Homophenylalanine	0.82	0.0012
D-glucosamine 6-phosphate	1-Stearoyl-2-oleoyl-sn-glycero-3-phosphoethanolamine	-0.82	0.0012
PC 34:2	Glycine deoxycholic acid	0.82	0.0012
PC 34:2	Disulfoton	0.82	0.0012
Nateglinide	Val-Ala-Ile-Thr-Val-Leu-Val-Lys	0.82	0.0012
2-chlorobenzoic acid	1-Stearoyl-2-oleoyl-sn-glycero-3-phosphoethanolamine	-0.81	0.0014
5'-hydroxymethyl-5'-desmethylmeloxicam	Disulfoton	0.80	0.0017
2-chlorobenzoic acid	1-Palmitoyl-2-docosahexaenoyl-sn-glycero-3-phosphocholine	0.80	0.0019
1-palmitoyl-2-linoleoyl-sn-glycero-3-phosphocholine	Disulfoton	0.80	0.0020
Nateglinide	Phe-Thr-Pro-Thr-Glu	0.79	0.0024
1-palmitoyl-2-linoleoyl-sn-glycero-3-phosphocholine	Taurochenodesoxycholic Acid	0.79	0.0024
Nateglinide	(2E,4E)-N-(2-Methylpropyl)octadeca-2,4-dienamide	-0.78	0.0027
2-chlorobenzoic acid	L-Homophenylalanine	0.78	0.0028
2-chlorobenzoic acid	Lys-Tyr-Gln-Glu-Ala	0.78	0.0030
1-palmitoyl-2-linoleoyl-sn-glycero-3-phosphocholine	Glycocholic Acid	0.77	0.0032

5'-hydroxymethyl-5'-desmethylmeloxicam	1-Stearoyl-2-oleoyl-sn-glycero-3-phosphoethanolamine	-0.77	0.0034
2-chlorobenzoic acid	Arachidonoyl thio-PC	0.77	0.0035
5'-hydroxymethyl-5'-desmethylmeloxicam	Val-Ala-Ile-Thr-Val-Leu-Val-Lys	-0.77	0.0036
Nateglinide	Disulfoton	-0.77	0.0037
3-dehydrocholic acid	1-Stearoyl-2-oleoyl-sn-glycero-3-phosphoethanolamine	0.76	0.0038
Leu-Tyr	1-Stearoyl-2-oleoyl-sn-glycero-3-phosphoethanolamine	0.76	0.0038
D-glucosamine 6-phosphate	Val-Ala-Ile-Thr-Val-Leu-Val-Lys	-0.76	0.0044
1-palmitoyl-2-linoleoyl-sn-glycero-3-phosphocholine	Glycine deoxycholic acid	0.76	0.0044
Telmisartan	1-Stearoyl-2-oleoyl-sn-glycero-3-phosphoethanolamine	0.78	0.0045
(2e,4e)-n-[2-(4-hydroxyphenyl)ethyl]dodeca-2,4-dienamide	5-[1-(Phenylmethyl)-1H-indazol-3-yl]-2-furanmethanol	0.75	0.0047
Leu-Tyr	5-[1-(Phenylmethyl)-1H-indazol-3-yl]-2-furanmethanol	0.75	0.0049
Lupulone	Phe-Thr-Pro-Thr-Glu	0.75	0.0049
3-dehydrocholic acid	Val-Ala-Ile-Thr-Val-Leu-Val-Lys	0.75	0.0052
5'-hydroxymethyl-5'-desmethylmeloxicam	(2R,3R,4S)-2-[(1R)-1,2-dihydroxyethyl]oxolane-3,4-diol	0.74	0.0054
D-glucosamine 6-phosphate	Disulfoton	0.74	0.0055
5'-hydroxymethyl-5'-desmethylmeloxicam	Carmustine	0.74	0.0056
3.alpha.,7.alpha.-dihydroxy-12-oxocholanoic acid	Ser-Ile-Lys-Gly-Lys	0.74	0.0059
Isodeoxycholic acid	Ser-Ile-Lys-Gly-Lys	0.74	0.0060
Lupulone	5-[1-(Phenylmethyl)-1H-indazol-3-yl]-2-furanmethanol	0.74	0.0061
(2e,4e)-n-[2-(4-hydroxyphenyl)ethyl]dodeca-2,4-dienamide	Phe-Thr-Pro-Thr-Glu	0.74	0.0061
Leu-Tyr	Phe-Thr-Pro-Thr-Glu	0.73	0.0065
2-chlorobenzoic acid	(2E,4E)-N-(2-Methylpropyl)octadeca-2,4-dienamide	0.73	0.0065
9s,11r,15s-trihydroxy-20a,20b-dihomo-5z,13e-prostadienoic acid	1-Stearoyl-2-oleoyl-sn-glycero-3-phosphoethanolamine	0.73	0.0066
2-cis-4-trans-abscisic acid	Phe-Thr-Pro-Thr-Glu	0.73	0.0070
Nateglinide	L-Homophenylalanine	-0.73	0.0071
D-glucosamine 6-phosphate	(2E,4E)-N-(2-Methylpropyl)octadeca-2,4-dienamide	0.73	0.0074
Nateglinide	For-met-ala-OH	0.72	0.0086
5'-hydroxymethyl-5'-desmethylmeloxicam	1-Palmitoyl-2-docosahexaenoyl-sn-glycero-3-phosphocholine	0.71	0.0091

Leu-Tyr	Val-Ala-Ile-Thr-Val-Leu-Val-Lys	0.71	0.0099
(2e,4e)-n-[2-(4-hydroxyphenyl)ethyl]dodeca-2,4-dienamide	Sesamex	0.71	0.0101
3.alpha.,7.alpha.-dihydroxy-12-oxocholanoic acid	4-[3-(Trifluoromethyl)phenoxy]aniline	0.71	0.0104
2-cis-4-trans-abscisic acid	5-[1-(Phenylmethyl)-1H-indazol-3-yl]-2-furanmethanol	0.70	0.0106
D-glucosamine 6-phosphate	Lys-Tyr-Gln-Glu-Ala	0.70	0.0111
Lupulone	1-Stearoyl-2-oleoyl-sn-glycero-3-phosphoethanolamine	0.70	0.0113
Nateglinide	(2R,3R,4S)-2-[(1R)-1,2-dihydroxyethyl]oxolane-3,4-diol	-0.70	0.0117
5'-hydroxymethyl-5'-desmethylmeloxicam	Arachidonoyl thio-PC	0.69	0.0127
D-glucosamine 6-phosphate	Arachidonoyl thio-PC	0.69	0.0135
9s,11r,15s-trihydroxy-20a,20b-dihomo-5z,13e-prostadienoic acid	For-met-ala-OH	0.69	0.0136
D-glucosamine 6-phosphate	For-met-ala-OH	-0.68	0.0143
D-glucosamine 6-phosphate	5-[1-(Phenylmethyl)-1H-indazol-3-yl]-2-furanmethanol	-0.68	0.0152
9s,11r,15s-trihydroxy-20a,20b-dihomo-5z,13e-prostadienoic acid	Val-Ala-Ile-Thr-Val-Leu-Val-Lys	0.68	0.0152
2-chlorobenzoic acid	1-(11Z-docosenoyl)-glycero-3-phosphate	0.68	0.0152
Isodeoxycholic acid	4-[3-(Trifluoromethyl)phenoxy]aniline	0.68	0.0152
Nateglinide	Bone Gla Protein (45-49)	0.68	0.0155
Leu-Tyr	Disulfoton	-0.67	0.0164
2-chlorobenzoic acid	Val-Ala-Ile-Thr-Val-Leu-Val-Lys	-0.67	0.0169
Leu-Tyr	(2E,4E)-N-(2-Methylpropyl)octadeca-2,4-dienamide	-0.67	0.0172
1-palmitoyl-2-linoleoyl-sn-glycero-3-phosphocholine	1-Palmitoyl-2-docosahexaenoyl-sn-glycero-3-phosphocholine	0.67	0.0173
Leu-Tyr	L-Homophenylalanine	-0.67	0.0181
1-palmitoyl-2-linoleoyl-sn-glycero-3-phosphocholine	1-Stearoyl-2-oleoyl-sn-glycero-3-phosphoethanolamine	-0.66	0.0190
D-glucosamine 6-phosphate	Phe-Thr-Pro-Thr-Glu	-0.66	0.0197
2-chlorobenzoic acid	Bone Gla Protein (45-49)	-0.66	0.0199
3-dehydrocholic acid	For-met-ala-OH	0.66	0.0199
(2e,4e)-n-[2-(4-hydroxyphenyl)ethyl]dodeca-2,4-dienamide	For-met-ala-OH	0.65	0.0214
5'-hydroxymethyl-5'-desmethylmeloxicam	Lys-Tyr-Gln-Glu-Ala	0.64	0.0237
9s,11r,15s-trihydroxy-20a,20b-dihomo-5z,13e-prostadienoic acid	Disulfoton	-0.64	0.0241

2-chlorobenzoic acid	Phe-Thr-Pro-Thr-Glu	-0.64	0.0243
2-chlorobenzoic acid	2-Hydrazino-6-methyl-1,3-benzothiazole	0.64	0.0244
3-dehydrocholic acid	Disulfoton	-0.64	0.0248
Lupulone	(2R,3R,4S)-2-[(1R)-1,2-dihydroxyethyl]oxolane-3,4-diol	-0.64	0.0254
2-chlorobenzoic acid	5-[1-(Phenylmethyl)-1H-indazol-3-yl]-2-furanmethanol	-0.63	0.0277
Lupulone	(2E,4E)-N-(2-Methylpropyl)octadeca-2,4-dienamide	-0.63	0.0281
Nateglinide	Lys-Tyr-Gln-Glu-Ala	-0.63	0.0292
Lupulone	Bone Gla Protein (45-49)	0.63	0.0294
5'-hydroxymethyl-5'-desmethylmeloxicam	Palmitoyl sphingomyelin	0.62	0.0316
5'-hydroxymethyl-5'-desmethylmeloxicam	Glycocholic Acid	0.62	0.0318
1-palmitoyl-2-linoleoyl-sn-glycero-3-phosphocholine	Lys-Tyr-Gln-Glu-Ala	0.62	0.0320
(2e,4e)-n-[2-(4-hydroxyphenyl)ethyl]dodeca-2,4-dienamide	Disulfoton	-0.62	0.0326
PC 34:2	1-Palmitoyl-2-docosahexaenoyl-sn-glycero-3-phosphocholine	0.62	0.0329
2-cis-4-trans-abscisic acid	Disulfoton	-0.62	0.0333
Nateglinide	Sesamex	0.61	0.0335
Telmisartan	Val-Ala-Ile-Thr-Val-Leu-Val-Lys	0.64	0.0345
(2e,4e)-n-[2-(4-hydroxyphenyl)ethyl]dodeca-2,4-dienamide	Bone Gla Protein (45-49)	0.61	0.0346
5'-hydroxymethyl-5'-desmethylmeloxicam	Glycine deoxycholic acid	0.61	0.0357
1-palmitoyl-2-linoleoyl-sn-glycero-3-phosphocholine	Val-Ala-Ile-Thr-Val-Leu-Val-Lys	-0.60	0.0373
1-palmitoyl-2-linoleoyl-sn-glycero-3-phosphocholine	Arachidonoyl thio-PC	0.60	0.0390
PC 34:2	1-Stearoyl-2-oleoyl-sn-glycero-3-phosphoethanolamine	-0.60	0.0402
Nateglinide	Arachidonoyl thio-PC	-0.60	0.0407
(2e,4e)-n-[2-(4-hydroxyphenyl)ethyl]dodeca-2,4-dienamide	Val-Ala-Ile-Thr-Val-Leu-Val-Lys	0.60	0.0411
5'-hydroxymethyl-5'-desmethylmeloxicam	For-met-ala-OH	-0.59	0.0414
(2e,4e)-n-[2-(4-hydroxyphenyl)ethyl]dodeca-2,4-dienamide	1-Stearoyl-2-oleoyl-sn-glycero-3-phosphoethanolamine	0.59	0.0429
PC 34:2	Val-Ala-Ile-Thr-Val-Leu-Val-Lys	-0.59	0.0431
3-dehydrocholic acid	Bone Gla Protein (45-49)	0.59	0.0434
2-cis-4-trans-abscisic acid	L-Homophenylalanine	-0.59	0.0453

Leu-Tyr	Bone Gla Protein (45-49)	0.58	0.0461
Leu-Tyr	(2R,3R,4S)-2-[(1R)-1,2-dihydroxyethyl]oxolane-3,4-diol	-0.58	0.0462
5'-hydroxymethyl-5'-desmethylmeloxicam	Ser-Ile-Lys-Gly-Lys	-0.58	0.0465
5'-hydroxymethyl-5'-desmethylmeloxicam	Phe-Thr-Pro-Thr-Glu	-0.58	0.0466
(2e,4e)-n-[2-(4-hydroxyphenyl)ethyl]dodeca-2,4-dienamide	L-Homophenylalanine	-0.58	0.0467
5'-hydroxymethyl-5'-desmethylmeloxicam	(2E,4E)-N-(2-Methylpropyl)octadeca-2,4-dienamide	0.58	0.0477
2-chlorobenzoic acid	Ser-Ile-Lys-Gly-Lys	-0.58	0.0498

Supplementary Table 4a Pearson correlation between serum metabolites and gut microbiota (genus) in cynomolgus monkeys			
**Serum metabolites**	**Genus**	***r***	***p***
**Palmitoyl sphingomyelin**	***Streptococcus***	**0.98**	**3.28E-08**
**Palmitoyl sphingomyelin**	***Colwellia***	**0.96**	**7.16E-07**
**1-(11Z-docosenoyl)-glycero-3-phosphate**	***Pseudobutyrivibrio***	**0.96**	**1.10E-06**
**Glycocholic Acid**	***Mesoplasma***	**0.95**	**2.16E-06**
**Palmitoyl sphingomyelin**	***Hathewaya***	**0.94**	**4.83E-06**
**Palmitoyl sphingomyelin**	***Polaribacter***	**0.94**	**5.08E-06**
**Palmitoyl sphingomyelin**	***Virgibacillus***	**0.94**	**6.26E-06**
**Taurochenodesoxycholic Acid**	***Polaribacter***	**0.93**	**1.05E-05**
**Taurochenodesoxycholic Acid**	***Streptococcus***	**0.93**	**1.49E-05**
**SM(d18:1/16:0)**	***Pseudobutyrivibrio***	**0.93**	**1.58E-05**
**Taurochenodesoxycholic Acid**	***Crassaminicella***	**0.92**	**1.71E-05**
**Taurochenodesoxycholic Acid**	***Gemella***	**0.92**	**1.81E-05**
**Taurochenodesoxycholic Acid**	***Mesoplasma***	**0.92**	**1.90E-05**
**Taurochenodesoxycholic Acid**	***Hathewaya***	**0.92**	**1.90E-05**
**Glycocholic Acid**	***Crassaminicella***	**0.92**	**1.97E-05**
**Taurochenodesoxycholic Acid**	***Colwellia***	**0.92**	**2.02E-05**
**Taurochenodesoxycholic Acid**	***Blattabacterium***	**0.92**	**2.06E-05**
**Palmitoyl sphingomyelin**	***Jeotgalibaca***	**0.92**	**2.62E-05**
**Palmitoyl sphingomyelin**	***Acholeplasma***	**0.92**	**2.73E-05**
**Taurochenodesoxycholic Acid**	***Mycoplasma***	**0.92**	**2.89E-05**
**SM(d18:1/16:0)**	***Butyrivibrio***	**0.91**	**3.04E-05**
**Taurochenodesoxycholic Acid**	***Buchnera***	**0.91**	**3.17E-05**
**Palmitoyl sphingomyelin**	***Buchnera***	**0.91**	**3.21E-05**
**Lys-Tyr-Gln-Glu-Ala**	***Butyrivibrio***	**0.91**	**3.29E-05**
**Taurochenodesoxycholic Acid**	***Acholeplasma***	**0.91**	**3.46E-05**
**Taurochenodesoxycholic Acid**	***Spiroplasma***	**0.91**	**4.10E-05**
**Palmitoyl sphingomyelin**	***Crassaminicella***	**0.91**	**4.12E-05**
**Palmitoyl sphingomyelin**	***Borrelia***	**0.91**	**4.28E-05**
**Taurochenodesoxycholic Acid**	***Vagococcus***	**0.91**	**4.32E-05**
**Palmitoyl sphingomyelin**	***Leuconostoc***	**0.91**	**4.43E-05**
**Palmitoyl sphingomyelin**	***Absiella***	**0.91**	**4.54E-05**
**Palmitoyl sphingomyelin**	***Halobacteriovorax***	**0.91**	**5.09E-05**
**Palmitoyl sphingomyelin**	***Carnobacterium***	**0.90**	**5.35E-05**
**Palmitoyl sphingomyelin**	***Halocella***	**0.90**	**5.37E-05**
**Glycocholic Acid**	***Streptococcus***	**0.90**	**5.42E-05**
**Carmustine**	***Vagococcus***	**0.90**	**5.53E-05**
**1-(11Z-docosenoyl)-glycero-3-phosphate**	***Butyrivibrio***	**0.90**	**5.59E-05**
**Palmitoyl sphingomyelin**	***Lysinibacillus***	**0.90**	**5.69E-05**

**Palmitoyl sphingomyelin**	***Tetragenococcus***	**0.90**	**5.95E-05**
**Palmitoyl sphingomyelin**	***Wolbachia***	**0.90**	**5.99E-05**
**Glycocholic Acid**	***Mycoplasma***	**0.90**	**6.16E-05**
**Palmitoyl sphingomyelin**	***Mycoplasma***	**0.90**	**6.25E-05**
**Palmitoyl sphingomyelin**	***Gemella***	**0.90**	**6.29E-05**
**Palmitoyl sphingomyelin**	***Mesoplasma***	**0.90**	**6.45E-05**
**Palmitoyl sphingomyelin**	***Fusobacterium***	**0.90**	**6.98E-05**
**Palmitoyl sphingomyelin**	***Spiroplasma***	**0.90**	**7.05E-05**
**Taurochenodesoxycholic Acid**	***Carnobacterium***	**0.90**	**8.38E-05**
**Glycocholic Acid**	***Gemella***	**0.89**	**8.43E-05**
**Carmustine**	***Crassaminicella***	**0.89**	**8.71E-05**
**Glycocholic Acid**	***Colwellia***	**0.89**	**9.02E-05**
**Taurochenodesoxycholic Acid**	***Borrelia***	**0.89**	**9.19E-05**
**Palmitoyl sphingomyelin**	***Finegoldia***	**0.89**	**1.07E-04**
**Palmitoyl sphingomyelin**	***Entomoplasma***	**0.89**	**1.25E-04**
**Carmustine**	***Acholeplasma***	**0.89**	**1.27E-04**
**Glycocholic Acid**	***Spiroplasma***	**0.89**	**1.29E-04**
**Palmitoyl sphingomyelin**	***Gottschalkia***	**0.88**	**1.30E-04**
**Carmustine**	***Hathewaya***	**0.88**	**1.32E-04**
**Glycocholic Acid**	***Borrelia***	**0.88**	**1.32E-04**
**Taurochenodesoxycholic Acid**	***Entomoplasma***	**0.88**	**1.37E-04**
**Taurochenodesoxycholic Acid**	***Virgibacillus***	**0.88**	**1.51E-04**
**Carmustine**	***Mesoplasma***	**0.88**	**1.52E-04**
**Glycocholic Acid**	***Acholeplasma***	**0.88**	**1.54E-04**
**Carmustine**	***Mycoplasma***	**0.88**	**1.57E-04**
**Taurochenodesoxycholic Acid**	***Fusobacterium***	**0.88**	**1.77E-04**
**Carmustine**	***Streptococcus***	**0.87**	**1.97E-04**
**Palmitoyl sphingomyelin**	***Blattabacterium***	**0.87**	**1.98E-04**
**Glycocholic Acid**	***Vagococcus***	**0.87**	**2.02E-04**
**Glycocholic Acid**	***Hathewaya***	**0.87**	**2.13E-04**
**Glycocholic Acid**	***Fusobacterium***	**0.87**	**2.21E-04**
**Taurochenodesoxycholic Acid**	***Ehrlichia***	**0.87**	**2.37E-04**
**Disulfoton**	***Chlamydia***	**0.87**	**2.38E-04**
**Glycocholic Acid**	***Entomoplasma***	**0.87**	**2.43E-04**
**Glycocholic Acid**	***Blattabacterium***	**0.87**	**2.46E-04**
**Glycocholic Acid**	***Leuconostoc***	**0.87**	**2.50E-04**
**Glycocholic Acid**	***Buchnera***	**0.87**	**2.61E-04**
**Lys-Tyr-Gln-Glu-Ala**	***Pseudobutyrivibrio***	**0.87**	**2.62E-04**
**Glycocholic Acid**	***Absiella***	**0.87**	**2.62E-04**
**Disulfoton**	***Leuconostoc***	**0.87**	**2.63E-04**

**Palmitoyl sphingomyelin**	***Staphylococcus***	**0.87**	**2.64E-04**
**Taurochenodesoxycholic Acid**	***Oceanobacillus***	**0.87**	**2.68E-04**
**Palmitoyl sphingomyelin**	***Oceanobacillus***	**0.87**	**2.79E-04**
**Glycocholic Acid**	***Polaribacter***	**0.86**	**2.85E-04**
**Taurochenodesoxycholic Acid**	***Leuconostoc***	**0.86**	**2.87E-04**
**Taurochenodesoxycholic Acid**	***Tetragenococcus***	**0.86**	**3.03E-04**
**Taurochenodesoxycholic Acid**	***Jeotgalibaca***	**0.86**	**3.14E-04**
**Palmitoyl sphingomyelin**	***Ehrlichia***	**0.86**	**3.30E-04**
**Taurochenodesoxycholic Acid**	***Staphylococcus***	**0.86**	**3.33E-04**
**Taurochenodesoxycholic Acid**	***Absiella***	**0.86**	**3.39E-04**
**Glycocholic Acid**	***Halocella***	**0.86**	**3.64E-04**
**Taurochenodesoxycholic Acid**	***Halobacteriovorax***	**0.86**	**3.76E-04**
**Carmustine**	***Gemella***	**0.86**	**3.84E-04**
**Glycocholic Acid**	***Carnobacterium***	**0.86**	**3.92E-04**
**Lys-Tyr-Gln-Glu-Ala**	***Anaerocolumna***	**0.85**	**3.95E-04**
**Palmitoyl sphingomyelin**	***Paenisporosarcina***	**0.85**	**4.01E-04**
**Carmustine**	***Spiroplasma***	**0.85**	**4.13E-04**
**Disulfoton**	***Tetragenococcus***	**0.85**	**4.18E-04**
**Palmitoyl sphingomyelin**	***Vagococcus***	**0.85**	**4.23E-04**
**Carmustine**	***Polaribacter***	**0.85**	**4.92E-04**
**Glycocholic Acid**	***Virgibacillus***	**0.85**	**4.99E-04**
**Taurochenodesoxycholic Acid**	***Gottschalkia***	**0.85**	**5.15E-04**
**Disulfoton**	***Finegoldia***	**0.84**	**5.53E-04**
**Arachidonoyl thio-PC**	***Anaerocolumna***	**0.84**	**5.56E-04**
**Carmustine**	***Buchnera***	**0.84**	**5.74E-04**
**Arachidonoyl thio-PC**	***Butyrivibrio***	**0.84**	**5.84E-04**
**Palmitoyl sphingomyelin**	***Lacinutrix***	**0.84**	**6.28E-04**
**Carmustine**	***Carnobacterium***	**0.84**	**6.43E-04**
**Carmustine**	***Leuconostoc***	**0.84**	**6.47E-04**
**Palmitoyl sphingomyelin**	***Ezakiella***	**0.84**	**6.97E-04**
**Glycocholic Acid**	***Gottschalkia***	**0.84**	**7.08E-04**
**Disulfoton**	***Lacinutrix***	**0.83**	**7.28E-04**
**Disulfoton**	***Caloranaerobacter***	**0.83**	**7.50E-04**
**Carmustine**	***Colwellia***	**0.83**	**7.59E-04**
**Carmustine**	***Absiella***	**0.83**	**8.09E-04**
**Glycine deoxycholic acid**	***Hathewaya***	**0.83**	**8.11E-04**
**Glycocholic Acid**	***Tetragenococcus***	**0.83**	**8.13E-04**
**Disulfoton**	***Buchnera***	**0.83**	**8.33E-04**
**Carmustine**	***Virgibacillus***	**0.83**	**8.52E-04**
**Carmustine**	***Tetragenococcus***	**0.83**	**8.63E-04**

**Disulfoton**	***Alkaliphilus***	**0.83**	**8.87E-04**
**Taurochenodesoxycholic Acid**	***Paenisporosarcina***	**0.83**	**8.92E-04**
**Glycine deoxycholic acid**	***Mesoplasma***	**0.83**	**9.02E-04**
**Carmustine**	***Jeotgalibaca***	**0.83**	**9.35E-04**
**Carmustine**	***Ehrlichia***	**0.82**	**9.63E-04**
**Disulfoton**	***Hungateiclostridium***	**0.82**	**9.70E-04**
**Carmustine**	***Fusobacterium***	**0.82**	**9.78E-04**
**Glycocholic Acid**	***Oceanobacillus***	**0.82**	**0.0010**
**Disulfoton**	***Virgibacillus***	**0.82**	**0.0010**
**Carmustine**	***Borrelia***	**0.82**	**0.0010**
**Glycine deoxycholic acid**	***Streptococcus***	**0.82**	**0.0011**
**Taurochenodesoxycholic Acid**	***Halocella***	**0.82**	**0.0011**
**Carmustine**	***Oceanobacillus***	**0.82**	**0.0011**
**Taurochenodesoxycholic Acid**	***Finegoldia***	**0.82**	**0.0011**
**Disulfoton**	***Staphylococcus***	**0.82**	**0.0011**
**Disulfoton**	***Lysinibacillus***	**0.82**	**0.0011**
**Taurochenodesoxycholic Acid**	***Lysinibacillus***	**0.82**	**0.0011**
**Palmitoyl sphingomyelin**	***Clostridium***	**0.82**	**0.0012**
**Disulfoton**	***Borrelia***	**0.82**	**0.0012**
**Carmustine**	***Gottschalkia***	**0.82**	**0.0012**
**Glycocholic Acid**	***Staphylococcus***	**0.82**	**0.0012**
**Glycocholic Acid**	***Jeotgalibaca***	**0.82**	**0.0012**
**Disulfoton**	***Vagococcus***	**0.82**	**0.0012**
**Disulfoton**	***Spiroplasma***	**0.82**	**0.0012**
**Disulfoton**	***Fusobacterium***	**0.81**	**0.0013**
**Glycine deoxycholic acid**	***Colwellia***	**0.81**	**0.0013**
**Glycine deoxycholic acid**	***Crassaminicella***	**0.81**	**0.0013**
**Disulfoton**	***Absiella***	**0.81**	**0.0013**
**Palmitoyl sphingomyelin**	***Caloranaerobacter***	**0.81**	**0.0013**
**Carmustine**	***Entomoplasma***	**0.81**	**0.0013**
**Carmustine**	***Staphylococcus***	**0.81**	**0.0013**
**Disulfoton**	***Polaribacter***	**0.81**	**0.0014**
**Taurochenodesoxycholic Acid**	***Wolbachia***	**0.81**	**0.0014**
**Glycocholic Acid**	***Paenisporosarcina***	**0.81**	**0.0014**
**Palmitoyl sphingomyelin**	***Alkaliphilus***	**0.81**	**0.0014**
**Carmustine**	***Wolbachia***	**0.81**	**0.0014**
**Glycine deoxycholic acid**	***Polaribacter***	**0.81**	**0.0015**
**Carmustine**	***Blattabacterium***	**0.81**	**0.0016**
**Glycine deoxycholic acid**	***Borrelia***	**0.80**	**0.0016**
**Disulfoton**	***Colwellia***	**0.80**	**0.0016**

**Glycine deoxycholic acid**	***Blattabacterium***	**0.80**	**0.0016**
**Glycine deoxycholic acid**	***Vagococcus***	**0.80**	**0.0017**
**Disulfoton**	***Mycoplasma***	**0.80**	**0.0018**
**Carmustine**	***Finegoldia***	**0.80**	**0.0018**
**Glycocholic Acid**	***Ehrlichia***	**0.80**	**0.0018**
**Taurochenodesoxycholic Acid**	***Caloranaerobacter***	**0.80**	**0.0018**
**Glycine deoxycholic acid**	***Mycoplasma***	**0.80**	**0.0018**
**Disulfoton**	***Ezakiella***	**0.80**	**0.0019**
**Glycocholic Acid**	***Wolbachia***	**0.79**	**0.0020**
**Glycine deoxycholic acid**	***Gemella***	**0.79**	**0.0021**
**Disulfoton**	***Carnobacterium***	**0.79**	**0.0021**
**Carmustine**	***Halobacteriovorax***	**0.79**	**0.0021**
**Glycine deoxycholic acid**	***Spiroplasma***	**0.79**	**0.0021**
**Disulfoton**	***Ehrlichia***	**0.79**	**0.0022**
**Glycocholic Acid**	***Finegoldia***	**0.79**	**0.0022**
**Carmustine**	***Lysinibacillus***	**0.79**	**0.0022**
**Disulfoton**	***Oceanobacillus***	**0.79**	**0.0022**
**Disulfoton**	***Paenisporosarcina***	**0.79**	**0.0022**
**Disulfoton**	***Wolbachia***	**0.79**	**0.0022**
**Glycocholic Acid**	***Lysinibacillus***	**0.79**	**0.0022**
**Glycine deoxycholic acid**	***Buchnera***	**0.79**	**0.0023**
**Disulfoton**	***Acholeplasma***	**0.79**	**0.0023**
**Glycine deoxycholic acid**	***Acholeplasma***	**0.79**	**0.0023**
**Palmitoyl sphingomyelin**	***Fictibacillus***	**0.79**	**0.0023**
**Disulfoton**	***Jeotgalibaca***	**0.79**	**0.0024**
**Disulfoton**	***Hathewaya***	**0.79**	**0.0024**
**Glycine deoxycholic acid**	***Leuconostoc***	**0.78**	**0.0025**
**Disulfoton**	***Fictibacillus***	**0.78**	**0.0025**
**Arachidonoyl thio-PC**	***Paeniclostridium***	**0.78**	**0.0027**
**Glycine deoxycholic acid**	***Ehrlichia***	**0.78**	**0.0027**
**Disulfoton**	***Blattabacterium***	**0.78**	**0.0028**
**SM(d18:1/16:0)**	***Anaerocolumna***	**0.78**	**0.0028**
**Taurochenodesoxycholic Acid**	***Lacinutrix***	**0.78**	**0.0030**
**Disulfoton**	***Gottschalkia***	**0.78**	**0.0030**
**Disulfoton**	***Crassaminicella***	**0.78**	**0.0031**
**Carmustine**	***Halocella***	**0.77**	**0.0034**
**Glycine deoxycholic acid**	***Carnobacterium***	**0.77**	**0.0035**
**1-(11Z-docosenoyl)-glycero-3-phosphate**	***Anaerocolumna***	**0.77**	**0.0035**
**Glycocholic Acid**	***Lacinutrix***	**0.76**	**0.0037**
**Carmustine**	***Paenisporosarcina***	**0.76**	**0.0041**

**Disulfoton**	***Entomoplasma***	**0.76**	**0.0041**
**Glycine deoxycholic acid**	***Virgibacillus***	**0.76**	**0.0041**
**Disulfoton**	***Halocella***	**0.76**	**0.0042**
**Val-Ala-Ile-Thr-Val-Leu-Val-Lys**	***Cyclobacterium***	**-0.76**	**0.0042**
**Disulfoton**	***Mesoplasma***	**0.76**	**0.0043**
**Disulfoton**	***Gemella***	**0.75**	**0.0045**
**For-met-ala-OH**	***Chlamydia***	**-0.75**	**0.0046**
**Taurochenodesoxycholic Acid**	***Clostridium***	**0.75**	**0.0049**
**Glycine deoxycholic acid**	***Halobacteriovorax***	**0.75**	**0.0050**
**Taurochenodesoxycholic Acid**	***Ezakiella***	**0.75**	**0.0050**
**Glycocholic Acid**	***Halobacteriovorax***	**0.75**	**0.0051**
**Glycine deoxycholic acid**	***Paenisporosarcina***	**0.75**	**0.0051**
**Glycine deoxycholic acid**	***Tetragenococcus***	**0.75**	**0.0052**
**Palmitoyl sphingomyelin**	***Chlamydia***	**0.75**	**0.0054**
**Disulfoton**	***Halobacteriovorax***	**0.74**	**0.0055**
**Glycine deoxycholic acid**	***Halocella***	**0.74**	**0.0055**
**Palmitoyl sphingomyelin**	***Hungateiclostridium***	**0.74**	**0.0057**
**For-met-ala-OH**	***Fictibacillus***	**-0.74**	**0.0058**
**Glycocholic Acid**	***Caloranaerobacter***	**0.74**	**0.0058**
**Glycine deoxycholic acid**	***Fusobacterium***	**0.74**	**0.0060**
**Carmustine**	***Caloranaerobacter***	**0.74**	**0.0063**
**Glycine deoxycholic acid**	***Entomoplasma***	**0.74**	**0.0064**
**Palmitoyl sphingomyelin**	***Paeniclostridium***	**0.73**	**0.0066**
**Carmustine**	***Clostridium***	**0.73**	**0.0071**
**Arachidonoyl thio-PC**	***Pseudobutyrivibrio***	**0.73**	**0.0071**
**Glycine deoxycholic acid**	***Absiella***	**0.73**	**0.0072**
**Disulfoton**	***Clostridium***	**0.72**	**0.0077**
**Glycine deoxycholic acid**	***Gottschalkia***	**0.72**	**0.0078**
**L-Homophenylalanine**	***Jeotgalibaca***	**0.72**	**0.0079**
**Glycine deoxycholic acid**	***Oceanobacillus***	**0.72**	**0.0084**
**Glycine deoxycholic acid**	***Staphylococcus***	**0.72**	**0.0085**
**L-Homophenylalanine**	***Halobacteriovorax***	**0.72**	**0.0086**
**(2E,4E)-N-(2-Methylpropyl)octadeca-2,4-dienamide**	***Butyrivibrio***	**0.72**	**0.0088**
**Lys-Tyr-Gln-Glu-Ala**	***Thermoclostridium***	**0.72**	**0.0088**
**1-Stearoyl-2-oleoyl-sn-glycero-3-phosphoethanolamine**	***Cyclobacterium***	**-0.71**	**0.0092**
**Glycine deoxycholic acid**	***Jeotgalibaca***	**0.71**	**0.0094**
**Disulfoton**	***Streptococcus***	**0.71**	**0.0095**
**Carmustine**	***Lacinutrix***	**0.71**	**0.0102**
**L-Homophenylalanine**	***Hathewaya***	**0.71**	**0.0104**
**1-Stearoyl-2-oleoyl-sn-glycero-3-phosphoethanolamine**	***Thermoclostridium***	**-0.70**	**0.0109**

1-Palmitoyl-2-docosahexaenoyl-sn-glycero-3-phosphocholine	*Halocella*	0.70	0.0116
Glycine deoxycholic acid	*Lysinibacillus*	0.70	0.0117
For-met-ala-OH	*Hungateiclostridium*	-0.70	0.0118
L-Homophenylalanine	*Paenisporosarcina*	0.70	0.0121
L-Homophenylalanine	*Vagococcus*	0.69	0.0122
L-Homophenylalanine	*Acholeplasma*	0.69	0.0128
L-Homophenylalanine	*Alkaliphilus*	0.69	0.0129
Glycine deoxycholic acid	*Wolbachia*	0.69	0.0129
L-Homophenylalanine	*Carnobacterium*	0.69	0.0132
Val-Ala-Ile-Thr-Val-Leu-Val-Lys	*Lacinutrix*	-0.69	0.0132
Taurochenodesoxycholic Acid	*Alkaliphilus*	0.69	0.0132
Glycine deoxycholic acid	*Finegoldia*	0.69	0.0133
L-Homophenylalanine	*Wolbachia*	0.69	0.0133
Arachidonoyl thio-PC	*Fictibacillus*	0.69	0.0137
L-Homophenylalanine	*Leuconostoc*	0.69	0.0137
L-Homophenylalanine	*Clostridium*	0.69	0.0140
For-met-ala-OH	*Tetragenococcus*	-0.68	0.0142
Glycine deoxycholic acid	*Caloranaerobacter*	0.68	0.0144
Glycocholic Acid	*Clostridium*	0.68	0.0145
Glycocholic Acid	*Ezakiella*	0.68	0.0148
(2E,4E)-N-(2-Methylpropyl)octadeca-2,4-dienamide	*Anaerocolumna*	0.68	0.0153
L-Homophenylalanine	*Virgibacillus*	0.68	0.0155
For-met-ala-OH	*Leuconostoc*	-0.68	0.0158
L-Homophenylalanine	*Buchnera*	0.68	0.0159
L-Homophenylalanine	*Finegoldia*	0.67	0.0164
L-Homophenylalanine	*Absiella*	0.67	0.0165
For-met-ala-OH	*Ehrlichia*	-0.67	0.0168
Palmitoyl sphingomyelin	*Anaerocolumna*	0.67	0.0171
Glycine deoxycholic acid	*Lacinutrix*	0.67	0.0171
Carmustine	*Ezakiella*	0.67	0.0171
Arachidonoyl thio-PC	*Wolbachia*	0.67	0.0174
(2E,4E)-N-(2-Methylpropyl)octadeca-2,4-dienamide	*Labilibaculum*	0.66	0.0183
1-Palmitoyl-2-docosahexaenoyl-sn-glycero-3-phosphocholine	*Wolbachia*	0.66	0.0184
Val-Ala-Ile-Thr-Val-Leu-Val-Lys	*Hungateiclostridium*	-0.66	0.0185
For-met-ala-OH	*Halocella*	-0.66	0.0186
Disulfoton	*Paeniclostridium*	0.66	0.0189
1-Palmitoyl-2-docosahexaenoyl-sn-glycero-3-phosphocholine	*Butyrivibrio*	0.66	0.0189
5-[1-(Phenylmethyl)-1H-indazol-3-yl]-2-furanmethanol	*Cyclobacterium*	-0.66	0.0196
Val-Ala-Ile-Thr-Val-Leu-Val-Lys	*Chlamydia*	-0.66	0.0198
Lys-Tyr-Gln-Glu-Ala	*Paeniclostridium*	0.66	0.0201

**L-Homophenylalanine**	***Anaerocolumna***	**0.66**	**0.0202**
**1-Palmitoyl-2-docosahexaenoyl-sn-glycero-3-phosphocholine**	***Anaerocolumna***	**0.66**	**0.0204**
**1-Stearoyl-2-oleoyl-sn-glycero-3-phosphoethanolamine**	***Paeniclostridium***	**-0.66**	**0.0205**
**L-Homophenylalanine**	***Thermoclostridium***	**0.66**	**0.0205**
**L-Homophenylalanine**	***Spiroplasma***	**0.65**	**0.0208**
**For-met-ala-OH**	***Lacinutrix***	**-0.65**	**0.0210**
**For-met-ala-OH**	***Cyclobacterium***	**-0.65**	**0.0210**
**Lys-Tyr-Gln-Glu-Ala**	***Alkaliphilus***	**0.65**	**0.0212**
**L-Homophenylalanine**	***Fusobacterium***	**0.65**	**0.0215**
**L-Homophenylalanine**	***Lysinibacillus***	**0.65**	**0.0216**
**L-Homophenylalanine**	***Mycoplasma***	**0.65**	**0.0216**
**L-Homophenylalanine**	***Streptococcus***	**0.65**	**0.0222**
**Val-Ala-Ile-Thr-Val-Leu-Val-Lys**	***Leuconostoc***	**-0.65**	**0.0223**
**Taurochenodesoxycholic Acid**	***Hungateiclostridium***	**0.65**	**0.0229**
**1-Stearoyl-2-oleoyl-sn-glycero-3-phosphoethanolamine**	***Lacinutrix***	**-0.65**	**0.0231**
**1-Palmitoyl-2-docosahexaenoyl-sn-glycero-3-phosphocholine**	***Streptococcus***	**0.65**	**0.0231**
**Val-Ala-Ile-Thr-Val-Leu-Val-Lys**	***Fictibacillus***	**-0.64**	**0.0237**
**Carmustine**	***Alkaliphilus***	**0.64**	**0.0240**
**Disulfoton**	***Thermoclostridium***	**0.64**	**0.0245**
**L-Homophenylalanine**	***Crassaminicella***	**0.64**	**0.0247**
**Glycocholic Acid**	***Fictibacillus***	**0.64**	**0.0250**
**Taurochenodesoxycholic Acid**	***Fictibacillus***	**0.64**	**0.0252**
**L-Homophenylalanine**	***Gottschalkia***	**0.64**	**0.0252**
**Glycine deoxycholic acid**	***Ezakiella***	**0.64**	**0.0261**
**(2E,4E)-N-(2-Methylpropyl)octadeca-2,4-dienamide**	***Cyclobacterium***	**0.64**	**0.0262**
**Arachidonoyl thio-PC**	***Alkaliphilus***	**0.63**	**0.0273**
**Carmustine**	***Hungateiclostridium***	**0.63**	**0.0273**
**(2E,4E)-N-(2-Methylpropyl)octadeca-2,4-dienamide**	***Thermoclostridium***	**0.63**	**0.0275**
**5-[1-(Phenylmethyl)-1H-indazol-3-yl]-2-furanmethanol**	***Labilibaculum***	**-0.63**	**0.0276**
**L-Homophenylalanine**	***Ezakiella***	**0.63**	**0.0279**
**Glycocholic Acid**	***Alkaliphilus***	**0.63**	**0.0281**
**For-met-ala-OH**	***Ezakiella***	**-0.63**	**0.0281**
**L-Homophenylalanine**	***Polaribacter***	**0.63**	**0.0282**
**L-Homophenylalanine**	***Caloranaerobacter***	**0.63**	**0.0283**
**1-Stearoyl-2-oleoyl-sn-glycero-3-phosphoethanolamine**	***Alkaliphilus***	**-0.63**	**0.0287**
**Disulfoton**	***Cyclobacterium***	**0.63**	**0.0294**
**L-Homophenylalanine**	***Staphylococcus***	**0.63**	**0.0295**
**L-Homophenylalanine**	***Butyrivibrio***	**0.62**	**0.0298**
**Glycocholic Acid**	***Hungateiclostridium***	**0.62**	**0.0299**
**Arachidonoyl thio-PC**	***Lysinibacillus***	**0.62**	**0.0300**

For-met-ala-OH	*Finegoldia*	-0.62	0.0303
L-Homophenylalanine	*Gemella*	0.62	0.0310
For-met-ala-OH	*Absiella*	-0.62	0.0310
Taurochenodesoxycholic Acid	*Chlamydia*	0.62	0.0312
L-Homophenylalanine	*Blattabacterium*	0.62	0.0313
For-met-ala-OH	*Polaribacter*	-0.62	0.0320
5-[1-(Phenylmethyl)-1H-indazol-3-yl]-2-furanmethanol	*Fictibacillus*	-0.62	0.0328
Lys-Tyr-Gln-Glu-Ala	*Fictibacillus*	0.62	0.0328
1-Stearoyl-2-oleoyl-sn-glycero-3-phosphoethanolamine	*Hungateiclostridium*	-0.61	0.0335
L-Homophenylalanine	*Tetragenococcus*	0.61	0.0337
(2E,4E)-N-(2-Methylpropyl)octadeca-2,4-dienamide	*Fictibacillus*	0.61	0.0342
L-Homophenylalanine	*Oceanobacillus*	0.61	0.0342
L-Homophenylalanine	*Borrelia*	0.61	0.0342
Cystamine	*Fictibacillus*	-0.61	0.0343
1-Palmitoyl-2-docosahexaenoyl-sn-glycero-3-phosphocholine	*Paeniclostridium*	0.61	0.0351
L-Homophenylalanine	*Colwellia*	0.61	0.0355
For-met-ala-OH	*Alkaliphilus*	-0.61	0.0355
Glycocholic Acid	*Paeniclostridium*	0.61	0.0359
Phe-Thr-Pro-Thr-Glu	*Fictibacillus*	-0.61	0.0360
For-met-ala-OH	*Wolbachia*	-0.61	0.0369
Arachidonoyl thio-PC	*Clostridium*	0.61	0.0369
Cystamine	*Labilibaculum*	-0.61	0.0370
For-met-ala-OH	*Oceanobacillus*	-0.61	0.0370
Phe-Thr-Pro-Thr-Glu	*Cyclobacterium*	-0.60	0.0376
SM(d18:1/16:0)	*Thermoclostridium*	0.60	0.0378
L-Homophenylalanine	*Halocella*	0.60	0.0386
Carmustine	*Fictibacillus*	0.60	0.0387
Lys-Tyr-Gln-Glu-Ala	*Wolbachia*	0.60	0.0389
Phe-Thr-Pro-Thr-Glu	*Anaerocolumna*	-0.60	0.0390
1-Stearoyl-2-oleoyl-sn-glycero-3-phosphoethanolamine	*Wolbachia*	-0.60	0.0392
Bone Gla Protein (45-49)	*Cyclobacterium*	-0.60	0.0396
Val-Ala-Ile-Thr-Val-Leu-Val-Lys	*Halocella*	-0.60	0.0397
1-Stearoyl-2-oleoyl-sn-glycero-3-phosphoethanolamine	*Chlamydia*	-0.60	0.0401
Val-Ala-Ile-Thr-Val-Leu-Val-Lys	*Tetragenococcus*	-0.60	0.0407
(2E,4E)-N-(2-Methylpropyl)octadeca-2,4-dienamide	*Pseudobutyrivibrio*	0.60	0.0412
1-Stearoyl-2-oleoyl-sn-glycero-3-phosphoethanolamine	*Leuconostoc*	-0.59	0.0414
Ethylmalonic acid	*Labilibaculum*	-0.59	0.0415
Arachidonoyl thio-PC	*Hungateiclostridium*	0.59	0.0417
Ethylmalonic acid	*Fictibacillus*	-0.59	0.0418
Glycine deoxycholic acid	*Fictibacillus*	0.59	0.0418

**Glycine deoxycholic acid**	***Clostridium***	**0.59**	**0.0418**
**Glycocholic Acid**	***Chlamydia***	**0.59**	**0.0420**
**5-[1-(Phenylmethyl)-1H-indazol-3-yl]-2-furanmethanol**	***Chlamydia***	**-0.59**	**0.0439**
**For-met-ala-OH**	***Staphylococcus***	**-0.59**	**0.0440**
**For-met-ala-OH**	***Lysinibacillus***	**-0.59**	**0.0441**
**L-Homophenylalanine**	***Mesoplasma***	**0.59**	**0.0441**
**Val-Ala-Ile-Thr-Val-Leu-Val-Lys**	***Caloranaerobacter***	**-0.59**	**0.0443**
**Arachidonoyl thio-PC**	***Finegoldia***	**0.59**	**0.0444**
**5-[1-(Phenylmethyl)-1H-indazol-3-yl]-2-furanmethanol**	***Anaerocolumna***	**-0.59**	**0.0445**
**Carmustine**	***Paeniclostridium***	**0.59**	**0.0450**
**For-met-ala-OH**	***Hathewaya***	**-0.59**	**0.0456**
**Phe-Thr-Pro-Thr-Glu**	***Labilibaculum***	**-0.58**	**0.0459**
**5-[1-(Phenylmethyl)-1H-indazol-3-yl]-2-furanmethanol**	***Butyrivibrio***	**-0.58**	**0.0461**
**For-met-ala-OH**	***Colwellia***	**-0.58**	**0.0467**
**Lys-Tyr-Gln-Glu-Ala**	***Clostridium***	**0.58**	**0.0473**
**Arachidonoyl thio-PC**	***Thermoclostridium***	**0.58**	**0.0476**
**1-Palmitoyl-2-docosahexaenoyl-sn-glycero-3-phosphocholine**	***Jeotgalibaca***	**0.58**	**0.0480**
**L-Homophenylalanine**	***Hungateiclostridium***	**0.58**	**0.0481**
**1-Palmitoyl-2-docosahexaenoyl-sn-glycero-3-phosphocholine**	***Virgibacillus***	**0.58**	**0.0481**
**For-met-ala-OH**	***Fusobacterium***	**-0.58**	**0.0489**
**Taurochenodesoxycholic Acid**	***Paeniclostridium***	**0.58**	**0.0490**

Supplementary Table 4b Pearson correlation between serum metabolites and gut microbiota (species) in cynomolgus monkeys

Serum metabolites	Species	*r*	*p*
Palmitoyl sphingomyelin	*Streptococcus_lutetiensis*	0.97	8.98E-08
Palmitoyl sphingomyelin	*Streptococcus_gallolyticus*	0.97	1.40E-07
1-(11Z-docosenoyl)-glycero-3-phosphate	*Lactobacillus_agilis*	0.96	9.37E-07
1-(11Z-docosenoyl)-glycero-3-phosphate	*Pseudobutyrivibrio_xylanivorans*	0.96	1.19E-06
Taurochenodesoxycholic Acid	*Streptococcus_gallolyticus*	0.95	1.88E-06
Taurochenodesoxycholic Acid	*Treponema_phagedenis*	0.95	2.18E-06
Palmitoyl sphingomyelin	*Streptococcus_equinus*	0.94	5.35E-06
Palmitoyl sphingomyelin	*Acholeplasma_axanthum*	0.94	5.55E-06
Glycocholic Acid	*Streptococcus_gallolyticus*	0.93	8.86E-06
Taurochenodesoxycholic Acid	*Buchnera_aphidicola*	0.93	1.00E-05
Taurochenodesoxycholic Acid	*Streptococcus_lutetiensis*	0.93	1.05E-05
Palmitoyl sphingomyelin	*Treponema_phagedenis*	0.93	1.45E-05
Palmitoyl sphingomyelin	*Buchnera_aphidicola*	0.93	1.49E-05
SM(d18:1/16:0)	*Pseudobutyrivibrio_xylanivorans*	0.93	1.59E-05
Glycocholic Acid	*Treponema_phagedenis*	0.92	2.17E-05
Taurochenodesoxycholic Acid	*Clostridium_argentinense*	0.92	2.53E-05
Taurochenodesoxycholic Acid	*Streptococcus_equinus*	0.92	2.99E-05
Lys-Tyr-Gln-Glu-Ala	*Butyrivibrio_fibrisolvens*	0.91	3.22E-05
Palmitoyl sphingomyelin	*Finegoldia_magna*	0.91	3.36E-05

**Glycocholic Acid**	***Streptococcus_equinus***	**0.91**	**4.10E-05**
**Glycocholic Acid**	***Streptococcus_lutetiensis***	**0.91**	**4.32E-05**
**SM(d18:1/16:0)**	***Lactobacillus_agilis***	**0.91**	**4.69E-05**
**Taurochenodesoxycholic Acid**	***Acholeplasma_axanthum***	**0.90**	**5.77E-05**
**Palmitoyl sphingomyelin**	***Clostridium_argentinense***	**0.90**	**8.05E-05**
**Lys-Tyr-Gln-Glu-Ala**	***Butyrivibrio_hungatei***	**0.89**	**9.41E-05**
**Sesamex**	***Bacteroides_fragilis***	**0.89**	**9.46E-05**
**Palmitoyl sphingomyelin**	***Fusobacterium_ulcerans***	**0.89**	**0.0001**
**Glycocholic Acid**	***Clostridium_argentinense***	**0.89**	**0.0001**
**Carmustine**	***Streptococcus_gallolyticus***	**0.89**	**0.0001**
**Glycocholic Acid**	***Buchnera_aphidicola***	**0.89**	**0.0001**
**Carmustine**	***Acholeplasma_axanthum***	**0.89**	**0.0001**
**SM(d18:1/16:0)**	***Butyrivibrio_fibrisolvens***	**0.89**	**0.0001**
**1-(11Z-docosenoyl)-glycero-3-phosphate**	***Butyrivibrio_hungatei***	**0.89**	**0.0001**
**Glycocholic Acid**	***Acholeplasma_axanthum***	**0.89**	**0.0001**
**Palmitoyl sphingomyelin**	***Clostridium_saccharoperbutylacetonicum***	**0.88**	**0.0001**
**SM(d18:1/16:0)**	***Butyrivibrio_hungatei***	**0.88**	**0.0001**
**Palmitoyl sphingomyelin**	***Lachnospiraceae_bacterium***	**0.88**	**0.0001**
**Arachidonoyl thio-PC**	***Butyrivibrio_fibrisolvens***	**0.88**	**0.0002**
**Palmitoyl sphingomyelin**	***Clostridium_esterthеticum***	**0.88**	**0.0002**
**Taurochenodesoxycholic Acid**	***Clostridium_estertheticum***	**0.88**	**0.0002**
**Carmustine**	***Streptococcus_lutetiensis***	**0.87**	**0.0002**

**Lys-Tyr-Gln-Glu-Ala**	***Pseudobutyrivibrio_xylanivorans***	**0.87**	**0.0002**
**1-(11Z-docosenoyl)-glycero-3-phosphate**	***Butyrivibrio_fibrisolvens***	**0.87**	**0.0003**
**Taurochenodesoxycholic Acid**	***Fusobacterium_ulcerans***	**0.86**	**0.0003**
**Carmustine**	***Streptococcus_equinus***	**0.86**	**0.0003**
**Taurochenodesoxycholic Acid**	***Finegoldia_magna***	**0.86**	**0.0004**
**Carmustine**	***Buchnera_aphidicola***	**0.86**	**0.0004**
**Taurochenodesoxycholic Acid**	***Clostridium_saccharoperbutylacetonicum***	**0.85**	**0.0004**
**Arachidonoyl thio-PC**	***Anaerostipes_rhamnosivorans***	**0.85**	**0.0005**
**Disulfoton**	***Fusobacterium_ulcerans***	**0.85**	**0.0005**
**Glycine deoxycholic acid**	***Streptococcus_gallolyticus***	**0.84**	**0.0006**
**Palmitoyl sphingomyelin**	***Clostridium_butyricum***	**0.84**	**0.0006**
**Carmustine**	***Clostridium_estertheticum***	**0.84**	**0.0006**
**Arachidonoyl thio-PC**	***Butyrivibrio_hungatei***	**0.84**	**0.0006**
**Glycocholic Acid**	***Fusobacterium_ulcerans***	**0.84**	**0.0006**
**Glycocholic Acid**	***Clostridium_estertheticum***	**0.84**	**0.0007**
**Carmustine**	***Clostridium_argentinense***	**0.83**	**0.0008**
**Disulfoton**	***Finegoldia_magna***	**0.83**	**0.0008**
**Carmustine**	***Treponema_phagedenis***	**0.83**	**0.0008**
**Glycocholic Acid**	***Finegoldia_magna***	**0.83**	**0.0009**
**Lys-Tyr-Gln-Glu-Ala**	***Lactobacillus_agilis***	**0.83**	**0.0009**
**Glycine deoxycholic acid**	***Streptococcus_lutetiensis***	**0.83**	**0.0009**
**Carmustine**	***Clostridium_saccharoperbutylacetonicum***	**0.83**	**0.0009**

**Carmustine**	***Finegoldia_magna***	**0.83**	**0.0009**
**Lys-Tyr-Gln-Glu-Ala**	***Anaerostipes_rhamnosivorans***	**0.82**	**0.0010**
**Glycine deoxycholic acid**	***Treponema_phagedenis***	**0.82**	**0.0011**
**Lys-Tyr-Gln-Glu-Ala**	***Butyrivibrio_proteoclasticus***	**0.82**	**0.0011**
**Disulfoton**	***Buchnera_aphidicola***	**0.81**	**0.0014**
**Disulfoton**	***Clostridium_butyricum***	**0.81**	**0.0015**
**Glycine deoxycholic acid**	***Buchnera_aphidicola***	**0.81**	**0.0015**
**Glycine deoxycholic acid**	***Streptococcus_equinus***	**0.80**	**0.0016**
**Glycocholic Acid**	***Clostridium_saccharoperbutylacetonicum***	**0.79**	**0.0021**
**Glycine deoxycholic acid**	***Acholeplasma_axanthum***	**0.79**	**0.0023**
**Glycine deoxycholic acid**	***Clostridium_argentinense***	**0.79**	**0.0024**
**Carmustine**	***Lachnospiraceae_bacterium***	**0.78**	**0.0027**
**For-met-ala-OH**	***Bacteroides_fragilis***	**0.78**	**0.0028**
**Taurochenodesoxycholic Acid**	***Lachnospiraceae_bacterium***	**0.78**	**0.0029**
**Taurochenodesoxycholic Acid**	***Clostridium_butyricum***	**0.78**	**0.0029**
**Disulfoton**	***Acholeplasma_axanthum***	**0.78**	**0.0031**
**L-Homophenylalanine**	***Lachnospiraceae_bacterium***	**0.77**	**0.0031**
**SM(d18:1/16:0)**	***Butyrivibrio_proteoclasticus***	**0.77**	**0.0033**
**Disulfoton**	***Clostridium_argentinense***	**0.77**	**0.0034**
**Glycine deoxycholic acid**	***Fusobacterium_ulcerans***	**0.77**	**0.0036**
**SM(d18:1/16:0)**	***Anaerostipes_rhamnosivorans***	**0.77**	**0.0037**
**Disulfoton**	***Streptococcus_equinus***	**0.77**	**0.0037**

**Carmustine**	***Fusobacterium_ulcerans***	**0.76**	**0.0038**
**Palmitoyl sphingomyelin**	***Anaerostipes_rhamnosivorans***	**0.76**	**0.0045**
**1-(11Z-docosenoyl)-glycero-3-phosphate**	***Butyrivibrio_proteoclasticus***	**0.74**	**0.0057**
**Arachidonoyl thio-PC**	***Butyrivibrio_proteoclasticus***	**0.74**	**0.0058**
**Arachidonoyl thio-PC**	***Pseudobutyrivibrio_xylanivorans***	**0.74**	**0.0059**
**Glycocholic Acid**	***Clostridium_butyricum***	**0.74**	**0.0059**
**(2E,4E)-N-(2-Methylpropyl)octadeca-2,4-dienamide**	***Butyrivibrio_fibrisolvens***	**0.74**	**0.0061**
**Disulfoton**	***Clostridium_estertheticum***	**0.74**	**0.0065**
**Carmustine**	***Clostridium_butyricum***	**0.73**	**0.0065**
**1-(11Z-docosenoyl)-glycero-3-phosphate**	***Anaerostipes_rhamnosivorans***	**0.73**	**0.0068**
**Disulfoton**	***Treponema_phagedenis***	**0.73**	**0.0069**
**Glycocholic Acid**	***Lachnospiraceae_bacterium***	**0.73**	**0.0069**
**Glycine deoxycholic acid**	***Clostridium_estertheticum***	**0.73**	**0.0069**
**1-Palmitoyl-2-docosahexaenoyl-sn-glycero-3-phosphocholine**	***Butyrivibrio_fibrisolvens***	**0.73**	**0.0070**
**Glycine deoxycholic acid**	***Finegoldia_magna***	**0.73**	**0.0074**
**Disulfoton**	***Streptococcus_gallolyticus***	**0.72**	**0.0080**
**Disulfoton**	***Clostridium_saccharoperbutylacetonicum***	**0.72**	**0.0081**
**L-Homophenylalanine**	***Clostridium_saccharoperbutylacetonicum***	**0.72**	**0.0085**
**1-Palmitoyl-2-docosahexaenoyl-sn-glycero-3-phosphocholine**	***Anaerostipes_rhamnosivorans***	**0.72**	**0.0086**
**Glycine deoxycholic acid**	***Clostridium_saccharoperbutylacetonicum***	**0.72**	**0.0089**
**Lys-Tyr-Gln-Glu-Ala**	***Lachnospiraceae_bacterium***	**0.71**	**0.0092**
**L-Homophenylalanine**	***Acholeplasma_axanthum***	**0.71**	**0.0096**

**L-Homophenylalanine**	***Butyrivibrio_proteoclasticus***	**0.71**	**0.0100**
**Glycine deoxycholic acid**	***Lachnospiraceae_bacterium***	**0.71**	**0.0102**
**Arachidonoyl thio-PC**	***Lachnospiraceae_bacterium***	**0.71**	**0.0103**
**Disulfoton**	***Lachnospiraceae_bacterium***	**0.70**	**0.0107**
**1-Palmitoyl-2-docosahexaenoyl-sn-glycero-3-phosphocholine**	***Butyrivibrio_hungatei***	**0.70**	**0.0119**
**Palmitoyl sphingomyelin**	***Butyrivibrio_proteoclasticus***	**0.69**	**0.0123**
**L-Homophenylalanine**	***Anaerostipes_rhamnosivorans***	**0.68**	**0.0148**
**(2E,4E)-N-(2-Methylpropyl)octadeca-2,4-dienamide**	***Anaerostipes_rhamnosivorans***	**0.68**	**0.0153**
**Arachidonoyl thio-PC**	***Lactobacillus_agilis***	**0.68**	**0.0156**
**Disulfoton**	***Streptococcus_lutetiensis***	**0.67**	**0.0165**
**(2E,4E)-N-(2-Methylpropyl)octadeca-2,4-dienamide**	***Butyrivibrio_hungatei***	**0.67**	**0.0165**
**1-Palmitoyl-2-docosahexaenoyl-sn-glycero-3-phosphocholine**	***Lachnospiraceae_bacterium***	**0.67**	**0.0172**
**L-Homophenylalanine**	***Finegoldia_magna***	**0.67**	**0.0180**
**L-Homophenylalanine**	***Buchnera_aphidicola***	**0.66**	**0.0194**
**L-Homophenylalanine**	***Clostridium_estertheticum***	**0.66**	**0.0195**
**L-Homophenylalanine**	***Butyrivibrio_fibrisolvens***	**0.66**	**0.0195**
**(2R,3R,4S)-2-[(1R)-1,2-dihydroxyethyl]oxolane-3,4-diol**	***Bifidobacterium_pseudocatenulatum***	**-0.66**	**0.0197**
**Palmitoyl sphingomyelin**	***Butyrivibrio_fibrisolvens***	**0.65**	**0.0221**
**1-Palmitoyl-2-docosahexaenoyl-sn-glycero-3-phosphocholine**	***Streptococcus_lutetiensis***	**0.65**	**0.0226**
**L-Homophenylalanine**	***Streptococcus_lutetiensis***	**0.64**	**0.0240**
**L-Homophenylalanine**	***Clostridium_argentinense***	**0.64**	**0.0260**
**Glycine deoxycholic acid**	***Clostridium_butyricum***	**0.64**	**0.0262**

**(2E,4E)-N-(2-Methylpropyl)octadeca-2,4-dienamide**	***Butyrivibrio_proteoclasticus***	**0.63**	**0.0271**
**L-Homophenylalanine**	***Butyrivibrio_hungatei***	**0.63**	**0.0281**
**L-Homophenylalanine**	***Streptococcus_gallolyticus***	**0.63**	**0.0291**
**L-Homophenylalanine**	***Fusobacterium_ulcerans***	**0.63**	**0.0294**
**1-Palmitoyl-2-docosahexaenoyl-sn-glycero-3-phosphocholine**	***Acholeplasma_axanthum***	**0.63**	**0.0296**
**L-Homophenylalanine**	***Clostridium_butyricum***	**0.62**	**0.0303**
**5-[1-(Phenylmethyl)-1H-indazol-3-yl]-2-furanmethanol**	***Anaerostipes_rhamnosivorans***	**-0.61**	**0.0345**
**Val-Ala-Ile-Thr-Val-Leu-Val-Lys**	***Fusobacterium_ulcerans***	**-0.61**	**0.0349**
**Carmustine**	***Anaerostipes_rhamnosivorans***	**0.61**	**0.0353**
**For-met-ala-OH**	***Clostridium_butyricum***	**-0.61**	**0.0360**
**For-met-ala-OH**	***Finegoldia_magna***	**-0.60**	**0.0382**
**Phe-Thr-Pro-Thr-Glu**	***Anaerostipes_rhamnosivorans***	**-0.60**	**0.0385**
**(2E,4E)-N-(2-Methylpropyl)octadeca-2,4-dienamide**	***Pseudobutyrivibrio_xylanivorans***	**0.60**	**0.0394**
**1-Stearoyl-2-oleoyl-sn-glycero-3-phosphoethanolamine**	***Clostridium_butyricum***	**-0.60**	**0.0399**
**Palmitoyl sphingomyelin**	***Butyrivibrio_hungatei***	**0.60**	**0.0402**
**Glycocholic Acid**	***Anaerostipes_rhamnosivorans***	**0.59**	**0.0413**
**5-[1-(Phenylmethyl)-1H-indazol-3-yl]-2-furanmethanol**	***Butyrivibrio_fibrisolvens***	**-0.59**	**0.0414**
**L-Homophenylalanine**	***Streptococcus_equinus***	**0.59**	**0.0423**
**L-Homophenylalanine**	***Treponema_phagedenis***	**0.59**	**0.0423**
**1-Palmitoyl-2-docosahexaenoyl-sn-glycero-3-phosphocholine**	***Streptococcus_gallolyticus***	**0.59**	**0.0428**
**Disulfoton**	***Anaerostipes_rhamnosivorans***	**0.59**	**0.0443**
**Val-Ala-Ile-Thr-Val-Leu-Val-Lys**	***Clostridium_butyricum***	**-0.59**	**0.0449**

**SM(d18:1/16:0)**	***Lachnospiraceae_bacterium***	**0.58**	**0.0473**
**Disulfoton**	***Butyrivibrio_proteoclasticus***	**0.58**	**0.0492**
**1-(11Z-docosenoyl)-glycero-3-phosphate**	***Lachnospiraceae_bacterium***	**0.58**	**0.0493**

Supplementary Table 5a Pearson correlation between fecal metabolites and gut microbiota (genus) in cynomolgus monkeys			
**Fecal metabolites**	**Genus**	***r***	***p***
**Pc 34:2**	***Carnobacterium***	**0.952418654**	**1.77288E-06**
**Pc 34:2**	***Jeotgalibaca***	**0.941742833**	**4.79022E-06**
**Pc 34:2**	***Hathewaya***	**0.941676613**	**4.81696E-06**
**Pc 34:2**	***Buchnera***	**0.935756028**	**7.73275E-06**
**Pc 34:2**	***Leuconostoc***	**0.931733032**	**1.04049E-05**
**Pc 34:2**	***Paenisporosarcina***	**0.929804422**	**1.19207E-05**
**Pc 34:2**	***Acholeplasma***	**0.920908526**	**2.13193E-05**
**Pc 34:2**	***Halobacteriovorax***	**0.9209071**	**2.13212E-05**
**Pc 34:2**	***Virgibacillus***	**0.917330403**	**2.64351E-05**
**Pc 34:2**	***Absiella***	**0.916721611**	**2.73942E-05**
**1-palmitoyl-2-linoleoyl-sn-glycero-3-phosphocholine**	***Jeotgalibaca***	**0.916690242**	**2.74444E-05**
**Pc 34:2**	***Streptococcus***	**0.914880729**	**3.04622E-05**
**Pc 34:2**	***Spiroplasma***	**0.914257365**	**3.15601E-05**
**Pc 34:2**	***Colwellia***	**0.9133084**	**3.32912E-05**
**1-palmitoyl-2-linoleoyl-sn-glycero-3-phosphocholine**	***Carnobacterium***	**0.912658497**	**3.45193E-05**
**Pc 34:2**	***Blattabacterium***	**0.912480586**	**3.48616E-05**
**Pc 34:2**	***Polaribacter***	**0.905658174**	**5.01455E-05**
**1-palmitoyl-2-linoleoyl-sn-glycero-3-phosphocholine**	***Halobacteriovorax***	**0.905251325**	**5.12001E-05**
**Pc 34:2**	***Mycoplasma***	**0.903977142**	**5.46162E-05**
**Pc 34:2**	***Fusobacterium***	**0.901673556**	**6.12447E-05**
**Pc 34:2**	***Gemella***	**0.900925599**	**6.35275E-05**
**1-palmitoyl-2-linoleoyl-sn-glycero-3-phosphocholine**	***Hathewaya***	**0.900317029**	**6.54338E-05**
**Pc 34:2**	***Vagococcus***	**0.899146293**	**6.9227E-05**
**1-palmitoyl-2-linoleoyl-sn-glycero-3-phosphocholine**	***Leuconostoc***	**0.898848167**	**7.02199E-05**
**1-palmitoyl-2-linoleoyl-sn-glycero-3-phosphocholine**	***Paenisporosarcina***	**0.89632332**	**7.90853E-05**
**Pc 34:2**	***Borrelia***	**0.891948366**	**9.65046E-05**

**Pc 34:2**	***Crassaminicella***	**0.890679015**	**0.000102082**
**Pc 34:2**	***Mesoplasma***	**0.881168877**	**0.000152367**
**1-palmitoyl-2-linoleoyl-sn-glycero-3-phosphocholine**	***Absiella***	**0.879947136**	**0.00016002**
**Pc 34:2**	***Wolbachia***	**0.877744941**	**0.000174573**
**Pc 34:2**	***Finegoldia***	**0.874691581**	**0.000196438**
**1-palmitoyl-2-linoleoyl-sn-glycero-3-phosphocholine**	***Streptococcus***	**0.873858431**	**0.000202759**
**1-palmitoyl-2-linoleoyl-sn-glycero-3-phosphocholine**	***Buchnera***	**0.873644819**	**0.000204406**
**1-palmitoyl-2-linoleoyl-sn-glycero-3-phosphocholine**	***Virgibacillus***	**0.87165695**	**0.000220231**
**Pc 34:2**	***Gottschalkia***	**0.869996976**	**0.000234164**
**Pc 34:2**	***Lysinibacillus***	**0.865463559**	**0.000275739**
**1-palmitoyl-2-linoleoyl-sn-glycero-3-phosphocholine**	***Acholeplasma***	**0.863449753**	**0.000295954**
**1-palmitoyl-2-linoleoyl-sn-glycero-3-phosphocholine**	***Colwellia***	**0.862807923**	**0.000302634**
**Pc 34:2**	***Clostridium***	**0.862311667**	**0.000307878**
**Pc 34:2**	***Tetragenococcus***	**0.858312677**	**0.000352771**
**Pc 34:2**	***Staphylococcus***	**0.857596231**	**0.000361324**
**1-palmitoyl-2-linoleoyl-sn-glycero-3-phosphocholine**	***Wolbachia***	**0.853129309**	**0.000418355**
**1-palmitoyl-2-linoleoyl-sn-glycero-3-phosphocholine**	***Clostridium***	**0.851015587**	**0.000447665**
**Pc 34:2**	***Halocella***	**0.848992894**	**0.000477181**
**Pc 34:2**	***Oceanobacillus***	**0.845612907**	**0.000529853**
**1-palmitoyl-2-linoleoyl-sn-glycero-3-phosphocholine**	***Spiroplasma***	**0.845258194**	**0.000535631**
**Pc 34:2**	***Caloranaerobacter***	**0.843659133**	**0.00056229**
**Pc 34:2**	***Alkaliphilus***	**0.842938847**	**0.000574629**
**1-palmitoyl-2-linoleoyl-sn-glycero-3-phosphocholine**	***Fusobacterium***	**0.841706343**	**0.000596228**
**1-palmitoyl-2-linoleoyl-sn-glycero-3-phosphocholine**	***Polaribacter***	**0.840659379**	**0.000615066**
**1-palmitoyl-2-linoleoyl-sn-glycero-3-phosphocholine**	***Finegoldia***	**0.834614484**	**0.000733019**
**1-palmitoyl-2-linoleoyl-sn-glycero-3-phosphocholine**	***Mycoplasma***	**0.834110466**	**0.00074359**
**1-palmitoyl-2-linoleoyl-sn-glycero-3-phosphocholine**	***Alkaliphilus***	**0.833153942**	**0.000763976**
**1-palmitoyl-2-linoleoyl-sn-glycero-3-phosphocholine**	***Blattabacterium***	**0.832494764**	**0.000778274**
**Pc 34:2**	***Entomoplasma***	**0.832086522**	**0.000787232**
**1-palmitoyl-2-linoleoyl-sn-glycero-3-phosphocholine**	***Lysinibacillus***	**0.830182618**	**0.000830068**

**1-palmitoyl-2-linoleoyl-sn-glycero-3-phosphocholine**	***Gemella***	**0.82845521**	**0.000870471**
**1-palmitoyl-2-linoleoyl-sn-glycero-3-phosphocholine**	***Borrelia***	**0.825525212**	**0.000942456**
**Pc 34:2**	***Ezakiella***	**0.823676252**	**0.00099019**
**1-palmitoyl-2-linoleoyl-sn-glycero-3-phosphocholine**	***Vagococcus***	**0.823404392**	**0.000997363**
**5'-hydroxymethyl-5'-desmethylmeloxicam**	***Paeniclostridium***	**0.821485845**	**0.001049129**
**1-palmitoyl-2-linoleoyl-sn-glycero-3-phosphocholine**	***Gottschalkia***	**0.820457981**	**0.001077701**
**Pc 34:2**	***Lacinutrix***	**0.820082849**	**0.001088276**
**Pc 34:2**	***Ehrlichia***	**0.819453053**	**0.001106211**
**1-palmitoyl-2-linoleoyl-sn-glycero-3-phosphocholine**	***Crassaminicella***	**0.816595885**	**0.001190453**
**1-palmitoyl-2-linoleoyl-sn-glycero-3-phosphocholine**	***Halocella***	**0.813428779**	**0.001289513**
**5'-hydroxymethyl-5'-desmethylmeloxicam**	***Wolbachia***	**0.813335601**	**0.00129252**
**1-palmitoyl-2-linoleoyl-sn-glycero-3-phosphocholine**	***Mesoplasma***	**0.806857317**	**0.001515202**
**D-glucosamine 6-phosphate**	***Thermoclostridium***	**0.804842765**	**0.001590118**
**5'-hydroxymethyl-5'-desmethylmeloxicam**	***Finegoldia***	**0.804812443**	**0.001591266**
**5'-hydroxymethyl-5'-desmethylmeloxicam**	***Lysinibacillus***	**0.804634569**	**0.001598019**
**1-palmitoyl-2-linoleoyl-sn-glycero-3-phosphocholine**	***Tetragenococcus***	**0.802973166**	**0.001662155**
**5'-hydroxymethyl-5'-desmethylmeloxicam**	***Gottschalkia***	**0.802060876**	**0.001698203**
**1-palmitoyl-2-linoleoyl-sn-glycero-3-phosphocholine**	***Ezakiella***	**0.801384404**	**0.001725317**
**5'-hydroxymethyl-5'-desmethylmeloxicam**	***Hungateiclostridium***	**0.798920355**	**0.001826897**
**D-glucosamine 6-phosphate**	***Hungateiclostridium***	**0.793093288**	**0.002085343**
**1-palmitoyl-2-linoleoyl-sn-glycero-3-phosphocholine**	***Staphylococcus***	**0.78787775**	**0.002339577**
**1-palmitoyl-2-linoleoyl-sn-glycero-3-phosphocholine**	***Anaerocolumna***	**0.787437612**	**0.002362066**
**1-palmitoyl-2-linoleoyl-sn-glycero-3-phosphocholine**	***Caloranaerobacter***	**0.78371696**	**0.002558838**
**1-palmitoyl-2-linoleoyl-sn-glycero-3-phosphocholine**	***Oceanobacillus***	**0.773298412**	**0.003176703**
**D-glucosamine 6-phosphate**	***Chlamydia***	**0.77116805**	**0.003315894**
**1-palmitoyl-2-linoleoyl-sn-glycero-3-phosphocholine**	***Lacinutrix***	**0.770617541**	**0.003352605**
**5'-hydroxymethyl-5'-desmethylmeloxicam**	***Caloranaerobacter***	**0.770453897**	**0.003363577**
**5'-hydroxymethyl-5'-desmethylmeloxicam**	***Clostridium***	**0.770340998**	**0.003371162**
**5'-hydroxymethyl-5'-desmethylmeloxicam**	***Virgibacillus***	**0.768515958**	**0.003495595**
**5'-hydroxymethyl-5'-desmethylmeloxicam**	***Acholeplasma***	**0.76141043**	**0.004013577**

**5'-hydroxymethyl-5'-desmethylmeloxicam**	***Staphylococcus***	**0.760481341**	**0.004085378**
**5'-hydroxymethyl-5'-desmethylmeloxicam**	***Leuconostoc***	**0.759955441**	**0.004126449**
**1-palmitoyl-2-linoleoyl-sn-glycero-3-phosphocholine**	***Paeniclostridium***	**0.757177629**	**0.004348599**
**1-palmitoyl-2-linoleoyl-sn-glycero-3-phosphocholine**	***Ehrlichia***	**0.755268398**	**0.004506454**
**5'-hydroxymethyl-5'-desmethylmeloxicam**	***Tetragenococcus***	**0.754629577**	**0.004560227**
**5'-hydroxymethyl-5'-desmethylmeloxicam**	***Borrelia***	**0.753702225**	**0.004639149**
**5'-hydroxymethyl-5'-desmethylmeloxicam**	***Absiella***	**0.751852412**	**0.004799657**
**5'-hydroxymethyl-5'-desmethylmeloxicam**	***Mycoplasma***	**0.751835904**	**0.004801108**
**5'-hydroxymethyl-5'-desmethylmeloxicam**	***Vagococcus***	**0.748549691**	**0.005096626**
**5'-hydroxymethyl-5'-desmethylmeloxicam**	***Hathewaya***	**0.747409521**	**0.005202298**
**5'-hydroxymethyl-5'-desmethylmeloxicam**	***Spiroplasma***	**0.74601953**	**0.005333351**
**Leu-Tyr**	***Cyclobacterium***	**-0.744867808**	**0.005443812**
**5'-hydroxymethyl-5'-desmethylmeloxicam**	***Fusobacterium***	**0.744375226**	**0.005491578**
**5'-hydroxymethyl-5'-desmethylmeloxicam**	***Oceanobacillus***	**0.744311229**	**0.005497807**
**1-palmitoyl-2-linoleoyl-sn-glycero-3-phosphocholine**	***Entomoplasma***	**0.743957829**	**0.005532301**
**5'-hydroxymethyl-5'-desmethylmeloxicam**	***Crassaminicella***	**0.742130697**	**0.005713245**
**D-glucosamine 6-phosphate**	***Labilibaculum***	**0.738654299**	**0.006069788**
**Pc 34:2**	***Paeniclostridium***	**0.736488475**	**0.006300223**
**Pc 34:2**	***Hungateiclostridium***	**0.735928761**	**0.006360829**
**5'-hydroxymethyl-5'-desmethylmeloxicam**	***Lacinutrix***	**0.729620475**	**0.00707461**
**Nateglinide**	***Cyclobacterium***	**-0.725508729**	**0.007571143**
**D-glucosamine 6-phosphate**	***Cyclobacterium***	**0.723618953**	**0.007807909**
**Pc 34:2**	***Fictibacillus***	**0.722813013**	**0.007910551**
**1-palmitoyl-2-linoleoyl-sn-glycero-3-phosphocholine**	***Fictibacillus***	**0.720647547**	**0.008191343**
**5'-hydroxymethyl-5'-desmethylmeloxicam**	***Buchnera***	**0.718663737**	**0.008455061**
**5'-hydroxymethyl-5'-desmethylmeloxicam**	***Carnobacterium***	**0.718518606**	**0.0084746**
**Pc 34:2**	***Anaerocolumna***	**0.716775928**	**0.008711861**
**5'-hydroxymethyl-5'-desmethylmeloxicam**	***Ehrlichia***	**0.715456189**	**0.008894815**
**5'-hydroxymethyl-5'-desmethylmeloxicam**	***Gemella***	**0.713829801**	**0.009124207**
**1-palmitoyl-2-linoleoyl-sn-glycero-3-phosphocholine**	***Butyrivibrio***	**0.712323731**	**0.009340538**

**Pc 34:2**	***Chlamydia***	**0.712222118**	**0.00935527**
**1-palmitoyl-2-linoleoyl-sn-glycero-3-phosphocholine**	***Hungateiclostridium***	**0.707900042**	**0.009998096**
**5'-hydroxymethyl-5'-desmethylmeloxicam**	***Mesoplasma***	**0.707764004**	**0.010018848**
**5'-hydroxymethyl-5'-desmethylmeloxicam**	***Alkaliphilus***	**0.70554934**	**0.010361226**
**D-glucosamine 6-phosphate**	***Paeniclostridium***	**0.702584013**	**0.010833188**
**D-glucosamine 6-phosphate**	***Fictibacillus***	**0.701681135**	**0.010980012**
**D-glucosamine 6-phosphate**	***Alkaliphilus***	**0.700809456**	**0.01112316**
**5'-hydroxymethyl-5'-desmethylmeloxicam**	***Entomoplasma***	**0.693597882**	**0.012361208**
**5'-hydroxymethyl-5'-desmethylmeloxicam**	***Streptococcus***	**0.690388018**	**0.012943897**
**5'-hydroxymethyl-5'-desmethylmeloxicam**	***Jeotgalibaca***	**0.689084503**	**0.013186233**
**1-palmitoyl-2-linoleoyl-sn-glycero-3-phosphocholine**	***Chlamydia***	**0.685897963**	**0.013792756**
**5'-hydroxymethyl-5'-desmethylmeloxicam**	***Paenisporosarcina***	**0.683557468**	**0.01425119**
**(2e,4e)-n-[2-(4-hydroxyphenyl)ethyl]dodeca-2,4-dienamide**	***Cyclobacterium***	**-0.675827352**	**0.015845384**
**5'-hydroxymethyl-5'-desmethylmeloxicam**	***Fictibacillus***	**0.671675916**	**0.016753779**
**5'-hydroxymethyl-5'-desmethylmeloxicam**	***Polaribacter***	**0.670870367**	**0.016934381**
**1-palmitoyl-2-linoleoyl-sn-glycero-3-phosphocholine**	***Thermoclostridium***	**0.66568725**	**0.018130808**
**5'-hydroxymethyl-5'-desmethylmeloxicam**	***Chlamydia***	**0.653498014**	**0.021187254**
**5'-hydroxymethyl-5'-desmethylmeloxicam**	***Anaerocolumna***	**0.652341365**	**0.021495594**
**5'-hydroxymethyl-5'-desmethylmeloxicam**	***Halobacteriovorax***	**0.647579151**	**0.022799637**
**2-chlorobenzoic acid**	***Paeniclostridium***	**0.644185213**	**0.023763467**
**D-glucosamine 6-phosphate**	***Lacinutrix***	**0.641563362**	**0.024528034**
**D-glucosamine 6-phosphate**	***Finegoldia***	**0.641238769**	**0.024623915**
**D-glucosamine 6-phosphate**	***Lysinibacillus***	**0.633290268**	**0.027057627**
**Lupulone**	***Paeniclostridium***	**-0.631705546**	**0.027562876**
**2-chlorobenzoic acid**	***Anaerocolumna***	**0.631282126**	**0.027699018**
**5'-hydroxymethyl-5'-desmethylmeloxicam**	***Halocella***	**0.623036771**	**0.030447987**
**5'-hydroxymethyl-5'-desmethylmeloxicam**	***Ezakiella***	**0.622663753**	**0.030576812**
**Leu-Tyr**	***Thermoclostridium***	**-0.619160567**	**0.031805861**
**3-dehydrocholic acid**	***Cyclobacterium***	**-0.61664622**	**0.032709579**
**2-cis-4-trans-abscisic acid**	***Cyclobacterium***	**-0.614392616**	**0.033535099**

**D-glucosamine 6-phosphate**	***Wolbachia***	**0.614298201**	**0.033570007**
**D-glucosamine 6-phosphate**	***Caloranaerobacter***	**0.613195104**	**0.033979782**
**D-glucosamine 6-phosphate**	***Clostridium***	**0.60982647**	**0.035253314**
**2-chlorobenzoic acid**	***Butyrivibrio***	**0.609642251**	**0.035323928**
**Pc 34:2**	***Butyrivibrio***	**0.606668355**	**0.036477911**
**9s,11r,15s-trihydroxy-20a,20b-dihomo-5z,13e-prostadienoic acid**	***Cyclobacterium***	**-0.606330553**	**0.036610671**
**5'-hydroxymethyl-5'-desmethylmeloxicam**	***Colwellia***	**0.60531076**	**0.037013552**
**Pc 34:2**	***Thermoclostridium***	**0.597353549**	**0.04026642**
**5'-hydroxymethyl-5'-desmethylmeloxicam**	***Thermoclostridium***	**0.594061176**	**0.041669917**
**5'-hydroxymethyl-5'-desmethylmeloxicam**	***Blattabacterium***	**0.593605121**	**0.041867026**
**D-glucosamine 6-phosphate**	***Tetragenococcus***	**0.590348791**	**0.043293685**
**Lupulone**	***Cyclobacterium***	**-0.583868903**	**0.04623443**
**Telmisartan**	***Thermoclostridium***	**-0.605807301**	**0.048221496**
**D-glucosamine 6-phosphate**	***Ezakiella***	**0.578927674**	**0.048569584**

Supplementary Table 5b Pearson correlation between fecal metabolites and gut microbiota (species) in cynomolgus monkeys			
**Fecal metabolites**	**Species**	***r***	***p***
**Pc 34:2**	***Clostridium_saccharoperbutylacetonicum***	**0.9354349**	**7.92361E-06**
**Pc 34:2**	***Buchnera_aphidicola***	**0.934193809**	**8.69654E-06**
**Pc 34:2**	***Clostridium_argentinense***	**0.93159986**	**1.05044E-05**
**Pc 34:2**	***Acholeplasma_axanthum***	**0.931386048**	**1.06657E-05**
**1-palmitoyl-2-linoleoyl-sn-glycero-3-phosphocholine**	***Lachnospiraceae_bacterium***	**0.930396897**	**1.14376E-05**
**Pc 34:2**	***Treponema_phagedenis***	**0.926066836**	**1.5352E-05**
**1-palmitoyl-2-linoleoyl-sn-glycero-3-phosphocholine**	***Clostridium_saccharoperbutylacetonicum***	**0.918440854**	**2.4754E-05**
**Pc 34:2**	***Streptococcus_lutetiensis***	**0.914699421**	**3.07784E-05**
**Pc 34:2**	***Streptococcus_gallolyticus***	**0.913076895**	**3.37247E-05**
**Pc 34:2**	***Fusobacterium_ulcerans***	**0.912165878**	**3.54736E-05**
**Pc 34:2**	***Lachnospiraceae_bacterium***	**0.912019685**	**3.57608E-05**
**Pc 34:2**	***Clostridium_estertheticum***	**0.903039322**	**5.72429E-05**
**Pc 34:2**	***Finegoldia_magna***	**0.890071009**	**0.000104842**
**1-palmitoyl-2-linoleoyl-sn-glycero-3-phosphocholine**	***Acholeplasma_axanthum***	**0.889814332**	**0.000106024**
**1-palmitoyl-2-linoleoyl-sn-glycero-3-phosphocholine**	***Streptococcus_lutetiensis***	**0.874655306**	**0.00019671**
**Pc 34:2**	***Streptococcus_equinus***	**0.872458938**	**0.000213735**
**1-palmitoyl-2-linoleoyl-sn-glycero-3-phosphocholine**	***Buchnera_aphidicola***	**0.867978248**	**0.000252025**
**1-palmitoyl-2-linoleoyl-sn-glycero-3-phosphocholine**	***Clostridium_argentinense***	**0.867445301**	**0.000256912**
**1-palmitoyl-2-linoleoyl-sn-glycero-3-phosphocholine**	***Fusobacterium_ulcerans***	**0.858687254**	**0.000348362**
**1-palmitoyl-2-linoleoyl-sn-glycero-3-phosphocholine**	***Anaerostipes_rhamnosivorans***	**0.855995559**	**0.000381017**
**1-palmitoyl-2-linoleoyl-sn-glycero-3-phosphocholine**	***Clostridium_estertheticum***	**0.855683848**	**0.000384947**
**1-palmitoyl-2-linoleoyl-sn-glycero-3-phosphocholine**	***Treponema_phagedenis***	**0.855556188**	**0.000386565**
**1-palmitoyl-2-linoleoyl-sn-glycero-3-phosphocholine**	***Streptococcus_gallolyticus***	**0.854491652**	**0.000400269**
**Pc 34:2**	***Clostridium_butyricum***	**0.843344605**	**0.000567653**

**1-palmitoyl-2-linoleoyl-sn-glycero-3-phosphocholine**	***Finegoldia_magna***	**0.842844084**	**0.000576268**
**1-palmitoyl-2-linoleoyl-sn-glycero-3-phosphocholine**	***Butyrivibrio_proteoclasticus***	**0.829977454**	**0.00083479**
**(2e,4e)-n-[2-(4-hydroxyphenyl)ethyl]dodeca-2,4-dienamide**	***Bacteroides_fragilis***	**0.813578403**	**0.001284695**
**1-palmitoyl-2-linoleoyl-sn-glycero-3-phosphocholine**	***Streptococcus_equinus***	**0.80557568**	**0.001562541**
**1-palmitoyl-2-linoleoyl-sn-glycero-3-phosphocholine**	***Clostridium_butyricum***	**0.80552422**	**0.001564465**
**5'-hydroxymethyl-5'-desmethylmeloxicam**	***Clostridium_butyricum***	**0.790032162**	**0.002231845**
**5'-hydroxymethyl-5'-desmethylmeloxicam**	***Finegoldia_magna***	**0.786659117**	**0.002402248**
**Pc 34:2**	***Anaerostipes_rhamnosivorans***	**0.786159789**	**0.002428294**
**1-palmitoyl-2-linoleoyl-sn-glycero-3-phosphocholine**	***Butyrivibrio_fibrisolvens***	**0.782465155**	**0.002627774**
**5'-hydroxymethyl-5'-desmethylmeloxicam**	***Acholeplasma_axanthum***	**0.767529659**	**0.003564278**
**Pc 34:2**	***Butyrivibrio_proteoclasticus***	**0.766820008**	**0.003614325**
**5'-hydroxymethyl-5'-desmethylmeloxicam**	***Streptococcus_equinus***	**0.743972256**	**0.005530889**
**5'-hydroxymethyl-5'-desmethylmeloxicam**	***Clostridium_estertheticum***	**0.743346166**	**0.005592386**
**1-palmitoyl-2-linoleoyl-sn-glycero-3-phosphocholine**	***Butyrivibrio_hungatei***	**0.730193782**	**0.00700737**
**5'-hydroxymethyl-5'-desmethylmeloxicam**	***Clostridium_saccharoperbutylacetonicum***	**0.724978181**	**0.007637064**
**5'-hydroxymethyl-5'-desmethylmeloxicam**	***Buchnera_aphidicola***	**0.704294849**	**0.010558984**
**5'-hydroxymethyl-5'-desmethylmeloxicam**	***Lachnospiraceae_bacterium***	**0.703490627**	**0.01068723**
**2-chlorobenzoic acid**	***Bifidobacterium_pseudocatenulatum***	**-0.691758249**	**0.012692726**
**Pc 34:2**	***Butyrivibrio_fibrisolvens***	**0.689537916**	**0.013101562**
**5'-hydroxymethyl-5'-desmethylmeloxicam**	***Streptococcus_gallolyticus***	**0.688085598**	**0.013374198**
**2-cis-4-trans-abscisic acid**	***Bacteroides_fragilis***	**0.683935988**	**0.014176298**
**5'-hydroxymethyl-5'-desmethylmeloxicam**	***Fusobacterium_ulcerans***	**0.6825345**	**0.01445505**
**5'-hydroxymethyl-5'-desmethylmeloxicam**	***Clostridium_argentinense***	**0.677617859**	**0.015464997**
**D-glucosamine 6-phosphate**	***Clostridium_butyricum***	**0.668664724**	**0.017436197**
**2-chlorobenzoic acid**	***Butyrivibrio_fibrisolvens***	**0.64797731**	**0.02268846**
**5'-hydroxymethyl-5'-desmethylmeloxicam**	***Streptococcus_lutetiensis***	**0.646527836**	**0.023095099**
**2-chlorobenzoic acid**	***Anaerostipes_rhamnosivorans***	**0.642636941**	**0.024212839**
**5'-hydroxymethyl-5'-desmethylmeloxicam**	***Anaerostipes_rhamnosivorans***	**0.63825915**	**0.025516807**

**Pc 34:2**	***Butyrivibrio_hungatei***	**0.633794302**	**0.026898341**
**2-chlorobenzoic acid**	***Butyrivibrio_hungatei***	**0.608852939**	**0.03562763**
**Telmisartan**	***Bifidobacterium_pseudocatenulatum***	**0.633246849**	**0.036480148**
**Nateglinide**	***Bacteroides_fragilis***	**0.606643829**	**0.036487539**
**5'-hydroxymethyl-5'-desmethylmeloxicam**	***Treponema_phagedenis***	**0.604322003**	**0.037407181**
**D-glucosamine 6-phosphate**	***Finegoldia_magna***	**0.585661666**	**0.045407136**

Supplementary Table 6 Primers used for qPCR analysis.

IL1β	Forward	GATGGCTTACTACAGCGGCA
	Reverse	AAGCCCTCGTTGTAGTGCTC
IL1R2	Forward	CTGACGTTTGCCCATGAAGG
	Reverse	ATTGTCAGTCTCGACCCCAGA
IL18RAP	Forward	CGAGACGCTGGGGGATAAAA
	Reverse	AATAGGCTCAGGGCCAAGTG
ADGRG1	Forward	CTGTCCAGCAAACACACACG
	Reverse	TTGCTTCATGCACGCTTCAC
GPR68	Forward	CCCGGTGGTCTATGTTACCG
	Reverse	GGCAGCGAGCAGATGTAGAA
PDGFRB	Forward	ATGCCTTACCACATCCGCTC
	Reverse	CACGTAGCCACTCTCAACCA
SLC5A3	Forward	GATGCAAGGAGGCCAGATGT
	Reverse	GCGCTTCCAGAAAATTGCCA
ATP1A3	Forward	GGACTTCACCTCCGAGCAAA
	Reverse	AGCCCTCCACAATGATGAGC
CLDND2	Forward	TCAGGCAGGAGTACTAGGGG
	Reverse	GTGGAGAGCACCATGAGGAC
MMP10	Forward	TTGAGCCTAAGGTCGATGCG
	Reverse	TGTCACCATCCTGGCATTGG
GAPDH	Forward	TCGGAGTCAACGGATTTGGT
	Reverse	TTGCCATGGGTGGAATCATA
